# MEMO: multi-experiment mixture model analysis of censored data

**DOI:** 10.1093/bioinformatics/btw190

**Published:** 2016-04-19

**Authors:** Eva-Maria Geissen, Jan Hasenauer, Stephanie Heinrich, Silke Hauf, Fabian J. Theis, Nicole E. Radde

**Affiliations:** ^1^Institute for Systems Theory and Automatic Control, University of Stuttgart, Stuttgart 70550, Germany; ^2^Institute of Computational Biology, Helmholtz Zentrum München – German Research Center for Environmental Health, Neuherberg 85764, Germany; ^3^Department of Mathematics, Technische Universität München, Garching 85748, Germany; ^4^Friedrich Miescher Laboratory of the Max Planck Society, Tübingen 72076, Germany

## Abstract

**Motivation:** The statistical analysis of single-cell data is a challenge in cell biological studies. Tailored statistical models and computational methods are required to resolve the subpopulation structure, i.e. to correctly identify and characterize subpopulations. These approaches also support the unraveling of sources of cell-to-cell variability. Finite mixture models have shown promise, but the available approaches are ill suited to the simultaneous consideration of data from multiple experimental conditions and to censored data. The prevalence and relevance of single-cell data and the lack of suitable computational analytics make automated methods, that are able to deal with the requirements posed by these data, necessary.

**Results:** We present MEMO, a flexible mixture modeling framework that enables the simultaneous, automated analysis of censored and uncensored data acquired under multiple experimental conditions. MEMO is based on maximum-likelihood inference and allows for testing competing hypotheses. MEMO can be applied to a variety of different single-cell data types. We demonstrate the advantages of MEMO by analyzing right and interval censored single-cell microscopy data. Our results show that an examination of censoring and the simultaneous consideration of different experimental conditions are necessary to reveal biologically meaningful subpopulation structures. MEMO allows for a stringent analysis of single-cell data and enables researchers to avoid misinterpretation of censored data. Therefore, MEMO is a valuable asset for all fields that infer the characteristics of populations by looking at single individuals such as cell biology and medicine.

**Availability and Implementation:** MEMO is implemented in MATLAB and freely available via github (https://github.com/MEMO-toolbox/MEMO).

**Contacts**: eva-maria.geissen@ist.uni-stuttgart.de or nicole.radde@ist.uni-stuttgart.de

**Supplementary information:** Supplementary data are available at *Bioinformatics* online.

## 1 Introduction

Cell-to-cell variability is omnipresent in biological systems (Balázsi *et al.*, 2011). Clonal populations can show quantitative differences in gene expression and qualitatively distinct cellular phenotypes and subpopulations (Eldar and Elowitz, 2010). The magnitude and nature of variability within a population can differ significantly depending on the system under consideration (Pelkmans, 2012). Accurate quantification relies on sophisticated statistical models which have to be tailored to the characteristics of the measurement technique (Bajikara *et al.*, 2014; Brennecke *et al.*, 2013; Duffy *et al.*, 2012; Pyne *et al.*, 2009).

One characteristic of experimental data, whose importance is often underestimated, is censoring (Duffy *et al.*, 2012). Most experimental devices provide censored data due to limited resolution or experimental constraints (see Supplementary Material, Section S.1). Left and right censored data provide upper and lower bounds, respectively, while interval censored data provide an interval for a quantity of interest (Escobar and Meeker, 1998). A quantity of interest might be the time to an event. If the event occurs before the start of the observation, the time to the event is left censored. Accordingly, the time to an event is right censored if the event does not occur during the observation or if a mutually exclusive event occurs before (Duffy *et al.*, 2012). These censoring events may also be randomly distributed. If the system is observed at discrete time points, only the time interval in which the event occurs is known. In concentration measurements censoring can occur due to detection limits or limited resolution. If the quantity of interest can only be detected above a certain detection limit, this limit provides an upper bound for quantities below, leading to left censored data. Similarly, saturation effects of the detection method lead to right censoring, where the saturation threshold serves as a lower bound for quantities above. Limited resolution naturally leads to interval censoring. For example, limited time-resolution in single-cell microscopy experiments is due to phototoxicity and photobleaching, requiring long inter-observation intervals to avoid stress (Schroeder, 2011).

Statistical models accounting for censoring are well-established. A suitable framework is provided by mixture modeling. While most mixture modeling approaches do not account for censoring (Johnsson *et al.*, 2016; Pyne *et al.*, 2009, 2014) others consider selected types of censoring (Lee and Scott, 2012; McLachlan and Jones, 1988) (see Supplementary Material, Section S.2). Unfortunately, the latter do not provide a comprehensive, easily accessible framework. Therefore, such models are infrequently applied in a biological context, which entails certain risks. In the presence of mutually exclusive (competing) biological events, for example, disregarding right censoring can result in an incorrect interpretation of experimental data such as correlations between actually uncorrelated data (Duffy *et al.*, 2012). Hence, there is a need for simple-to-use computational methods to analyze censored population data.

Besides censoring, another challenge for computational analysis methods of single-cell data is the integration of data from multiple experimental conditions (e.g. different strengths of stimuli or multiple sampling times after an intervention on the biological system at hand) or multiple technical and biological replicates. Established approaches use a two-step procedure for this purpose. First, individual samples are described independently with finite mixture models. Thereafter, matching-based methods are applied to link the different samples (Pyne *et al.*, 2009), e.g. to decide upon the appearance of identical subpopulations. These methods rely on similarity between distributions under different conditions. In the case of large changes in the corresponding distribution between experimental conditions, matching methods are not able to map the populations. To address this shortcoming, a Joint Clustering and Matching (JCM) approach (Pyne *et al.*, 2014) has been introduced. JCM allows for a more rigorous matching across samples and the consideration of inter-sample variability. For that purpose, a template model is fitted to the pooled samples and the individual samples are modeled as instances of this template by adding random effect terms to the template parameters. This approach is well-suited for analyzing the size of different distinct subpopulations in different samples. However, in case of small changes due to altered experimental conditions the subpopulation structure remains difficult to quantify. Furthermore, the template is constructed without considering inter-sample variability and a rigorous comparison of different biological hypotheses is not straight forward.

In order to cope with these challenges, we introduce MEMO, a Multi-Experiment mixture MOdeling framework which is able to analyze samples from different experimental conditions simultaneously, can account for censoring and compares competing model hypotheses. MEMO uses maximum-likelihood inference to determine the subpopulation structure and properties of heterogeneous cell populations. MEMO is implemented in MATLAB and freely available via github (https://github.com/MEMO-toolbox/MEMO). We expect that MEMO can be used for a broad spectrum of univariate, censored single-cell data, e.g. FACS, CyTOF, qPCR and time-lapse microscopy data. The data should - as for other statistical analysis methods - be appropriately preprocessed and the interesting dimension can be determined using dimension reduction methods (see, e.g. Angerer *et al.*, 2016).

In this study, we evaluate our method using simulated and experimental data. In particular, we analyze time-to-event single-cell microscopy data from multiple yeast strains. These cells were observed at discrete time points and only for a certain duration. Hence the data are interval and right censored. We found that, in contrast to naïve approaches, MEMO inferred the correct subpopulation structure for cases in which it was known. In addition, for more complex datasets containing multiple experimental conditions, MEMO revealed the existence of subpopulations for certain strains, some of which inherit the phenotypic properties of wild type cells. We demonstrate that MEMO even enables testing of competing hypotheses about underlying molecular mechanisms of the phenotype. In a second application on single-cell protein level data (Andres *et al.*, 2010) we demonstrate how mechanistic information can be integrated in MEMO.

Overall, our results demonstrate the additional benefit of a multi-experiment modeling framework and the importance of accounting for censoring, independent of how little it might be.

## 2 Approach

*Multi-experiment mixture modeling.* In the following, we introduce MEMO’s basics and the workflow. For this purpose we denote the quantity of interest by *X*. The distribution of *X* is described by a finite mixture model,

$$p(x|\theta ,u)=\sum_{s=1}^{S}w_{s}(u)\Phi (x|\varphi_{s}(u)),\;\sum_{s=1}^{S}w_{s}(u)=1$$

with subpopulation index s=1,…,S, subpopulation weight $w_{s}(u)$ and subpopulation mixture parameters $\varphi_{s}(u)$ for experimental condition *u*. The distributions Φ can be normal, log-normal, gamma or Johnson SU, with the latter being extremely flexible. Left, right and interval censoring of the distribution p(x|θ,u) yields distributions for observed genuine values (realizations within detection range), and observed censoring values (realizations out of detection range) (see the MEMO Documentation, Section D.2). Given a set of data, MEMO infers the mixture weights $w_{s}(u)$ and mixture parameters $\varphi_{s}(u)$. Hypotheses about the dependence of $w_{s}(u)$ and $\varphi_{s}(u)$ on the experimental condition *u* can be incorporated into functional dependencies $w_{s}=\text{fun}(\theta ,u)$ and $\varphi_{s}=\text{fun}(\theta ,u)$, parametrized with meta-parameters θ.

*Step 1. Formulation of competing model hypotheses.* For all competing hypotheses about subpopulation structures and condition dependence, the number of subpopulations *S* is selected and parametrization of $w_{s}(u)$ and $\varphi_{s}(u)$ is defined. As MEMO exploits symbolic calculations, any combinations of rudimentary functions are supported. In case of a normal distribution with mixture parameters $\varphi_{s}(u)=(\mu (u),\sigma (u))$, a stimulus induced shift towards lower values might for instance be described by a Hill-type function $\mu (u,\theta )=\mu_{0}u_{0}^{n}/(u_{0}^{n}+u^{n})$ with meta-parameters $\theta =(\mu_{0},u_{0})$. In a similar way positive dependencies and other dependencies on the condition *u* can be modeled. Furthermore, MEMO also allows for the independent modeling of subpopulations in different conditions, e.g. $\mu (u_{i},\theta )=\mu_{i}$ for condition index *i*. For details we refer to Sections 4.2 and 4.3 as well as to the MEMO Documentation, Section D.2.2. In addition to the subpopulation structure and properties, left or right censoring distributions can be parametrized. This is not strictly necessary, since MEMO can also use the tail probabilities. It however allows for additional linking of data collected under different experimental conditions and for a more detailed model-data comparison in the presence of distributed censoring (Supplementary Material, Figs S7 and S8) and straight-forward resampling of data, e.g. for a bootstrap based goodness of fit analysis (see Supplementary Material, Section S.4.2.2).

*Step 2. Parametrization of multi-experiment mixture models.* For the model hypotheses, maximum-likelihood estimation is employed to infer the unknown parameters θ (see Section 3 and the MEMO Documentation, Section D.2.3). MEMO uses an efficient global optimization method based on multi-start local optimization with analytically derived gradients (see the MEMO Documentation, Section D.2.6). This method outperformed other global optimization methods in a variety of test runs and provided accurate estimates (Raue *et al.*, 2013). The uncertainty of the estimated parameters can be assessed by using profile likelihood methods (Murphy and van der Vaart, 2000) or Bayesian statistics (Haario *et al.*, 2006), both supported by MEMO. The output of Step 2 is a set of parametrized models that correspond to different hypotheses, and the corresponding sets of parameters characterizing the subpopulations as well as model and prediction uncertainties.

*Step 3. Testing of hypotheses via model selection.* The competing model hypotheses can be compared in MEMO using model selection criteria, such as the Akaike information criterion (AIC), the Bayesian information criterion (BIC) or the likelihood ratio test (see the MEMO Documentation, Section D.2.4). For an automated analysis of the subpopulation structure a backward model selection algorithm is implemented, which enables unsupervised exploration.

*Step 4. Interpretation and further analysis.* Steps 1–3 provide a multi-experiment model or a set of mixture models capturing the data. MEMO supports visualization of these mixture models, as well as a model-data comparison (see the MEMO Documentation, Section D.2.5). The mixture model and its parameters can be used for subsequent analysis, e.g. to identify dependencies on input signals. Furthermore, the parameters may inform mechanistic modeling approaches (Heinrich *et al.*, 2013).

## 3 Materials and methods

### 3.1 Experimental data

The functionality of the spindle assembly checkpoint (SAC) was assessed using live-cell imaging on a DeltaVision Core system (Applied Precision/GE Healthcare), see Heinrich *et al.* (2013).

The NGF-induced Erk1/2 phosphorylation snapshot data were derived by quantitative automated microscopy (QuAM) as described in Hasenauer *et al.* (2014b).

### 3.2 Modeling of genuine value and censoring value generating processes

MEMO models genuine values and censoring values to be outcomes of different stochastic processes, in the sense of distributions, that compete for realization. Therefore, it discriminates between the distributions generating the data and the observed distributions of data, which differ if the supports of the outcomes of the data generating distributions overlap. MEMO implements normal, log-normal, gamma and Johnson-SU distributions to model the data generating distributions. For interval censored data, the probability of a data point lying within an inter-observation interval is computed by integrating over the respective part of the distributions.

The observed distributions are used to compute the likelihood of the data given the parameters. In the case of right censoring, the likelihood of data D with parameters θ is given by

$$p(D|\theta )\propto \prod_{i}\left(\prod_{j}p(x_{i}^{j},x_{i}^{j}\leq X_{i,\bar{c}}|\theta )\right)\left(\prod_{k}p({\bar{x}}_{i}^{k},{\bar{x}}_{i}^{k}\leq X_{i}|\theta )\right),\;$$

in which *i* indexes the experimental conditions while *j* and *k* index the measured cells. Since in this case right censoring is considered as competing process, the censoring quantity is denoted by $X_{i,\bar{c}}$. The uncensored and right censored data in experiment *i* are denoted by $x_{i}^{j}$ and ${\bar{x}}_{i}^{k}$, respectively. The likelihood consists of the probabilities of observing a value $x_{i}^{j}$ or a censoring value ${\bar{x}}_{i}^{k}$. These are given by the joint probability of a certain value together with the probability that this value is smaller than or equal to a realization of the competing process. For more details and different censoring types see the MEMO Documentation, Section D.2.1.

### 3.3 Model calibration

MEMO exploits multi-start local optimization with analytical derivatives to optimize the likelihood. These analytical derivatives are automatically derived from the model equations by using the Matlab Symbolic Math Toolbox. A space filling Latin hypercube initialization of the optimization with at least 100 starting points is used. Convergence is assessed visually using methods illustrated in Raue *et al.* (2013). MEMO provides Markov chain Monte Carlo methods (e.g. via the MATLAB toolbox DRAM (Haario *et al.*, 2006)) and profile likelihood methods (Murphy and van der Vaart, 2000) for uncertainty analysis. These methods enable an assessment of the model reliability and its predictive power in a rigorous statistical manner. For more details see the MEMO Documentation, Section D.2.3.

### 3.4 Model selection

To select the most likely subpopulation structure and parametrization, MEMO implements different model selection criteria. In this manuscript, the AIC, the BIC and the likelihood ratio test are used (and always provide the same results). As MEMO also implements Markov chain Monte Carlo methods, Bayes factors can also be calculated. In addition to the comparison of predefined models, MEMO also provides a backward model selection algorithm for the subpopulation structure. This enables a simple, user-friendly exploration of many model alternatives. For more details see the MEMO Documentation, Section D.2.4.

## 4 Results

### 4.1 Evaluation of MEMO for censored data

**Fig. 1.**
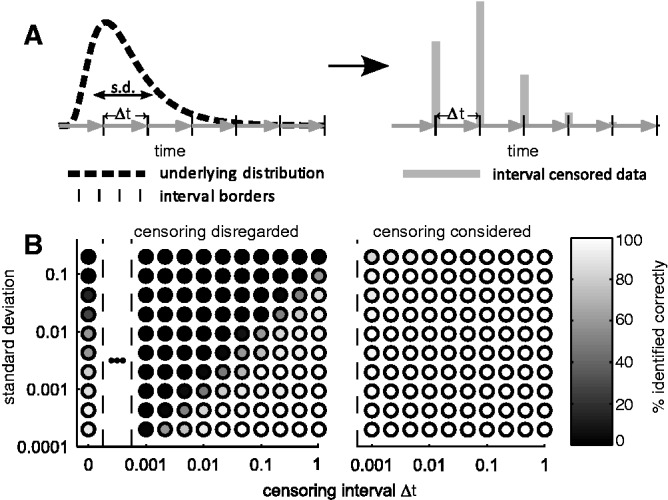
Inference of a log-normal distribution using interval censored data. (**A**) Illustration of interval censoring (left) and resulting observed distribution of 100 interval censored data points (right). (**B**) For distributions with different log-standard deviations and different inter-observation intervals Δt, the number of subpopulations is inferred using: (left) A model that does not account for interval censoring (= naïve approach); and (right) MEMO, which accounts for censoring. The color of the circles encodes the frequency with which the correct number of subpopulations, here one, is selected

Initially we evaluated the performance of MEMO using a variety of simulated test scenarios. In the following the quantities of interest are event times, such as time to division, differentiation or death. An event lying outside of the measurement range will be called a censoring time. We simulated interval censored data from a single log-normal distribution with known distribution parameters (Fig. 1A). Using these simulated data, we assessed how well the subpopulation structure, i.e. the number of subpopulations and their parameters, can be inferred. We used the BIC for model selection and found that naïve approaches that disregard censoring overestimated the number of subpopulations, in particular for shorter inter-observation intervals (Fig. 1B, left). The overestimation was worse for shorter censoring intervals, since the optimization frequently placed subpopulations at the end of individual censoring intervals. A single distribution was correctly identified only in case that all data lie within one inter-observation interval, which is the case when the standard deviation of the log-normal distribution is small compared to the censoring interval. In contrast, MEMO accurately reconstructed the underlying distribution and determined the correct subpopulation structure independently of the censoring interval. For simple distributions already small samples were sufficient to achieve an accurate reconstruction from interval censored data (Fig. 1B, right and Supplementary Material, Section S.3, Fig. S1).

**Fig. 2.**
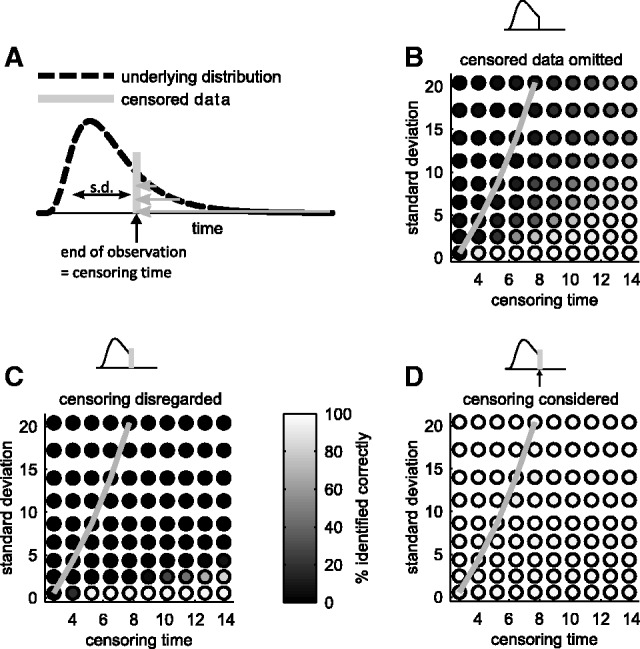
Inference of a log-normal distribution using right censored data. (**A**) Illustration of right censoring and resulting observed distribution of data points after right censoring. Three different approaches were used to fit a model with one to five subpopulations: Models that do not account for censoring and (**B**) censored data are omitted, or (**C**) the values of censored data are set to censoring times, and (**D**) MEMO, which accounts for censoring. The color of the circles encodes the frequency with which the correct number of subpopulations, here one, is selected. As a reference, the grey curve indicates points for which the censoring time equals the mean of the underlying distribution

A similar evaluation for right censored data yielded comparable results (Fig. 2A): Simply omitting the right censored data points as well as disregarding censoring results in an overestimation of the number of subpopulations (Fig. 2B and C). In both cases the number of subpopulations was correctly inferred only when the censoring time is much greater than the distribution mean. By contrast, MEMO was able to reproduce the correct results in any case (Fig. 2D and Supplementary Material, Section S.3, Fig. S2). Thus, statistical models implemented in MEMO, which account for censoring, are more reliable than naïve analysis approaches and provide an accurate estimate for the number of subpopulations.

### 4.2 Analysis of SAC time-to-event microscopy data

**Fig. 3.**
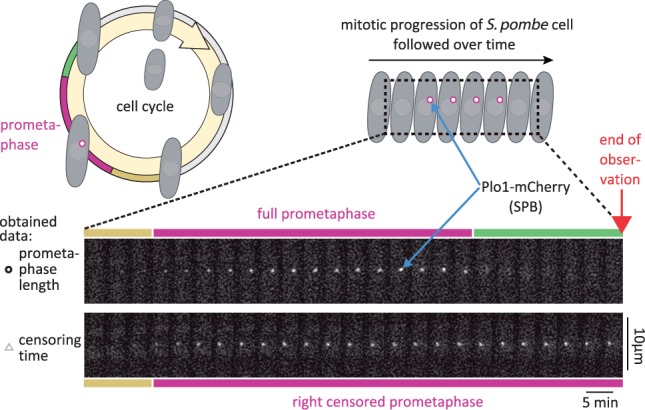
Fluorescence microscopy live-cell imaging of different *S. pombe* strains to assess mitosis times. Localization of Plo1-mCherry to spindle pole bodies (SPB) was used to determine the prometaphase lengths. Cells are imaged every 5 min for 17 h causing interval and right censoring (Color version of this figure is available at *Bioinformatics* online.)

For a realistic evaluation, we considered data from a study of the functionality of the SAC in *Schizosaccharomyces pombe* (*S.* *pombe*) (Heinrich *et al.*, 2013). The SAC is a signaling pathway that protects genome integrity by detecting and responding to errors in chromosome attachment during mitosis (London and Biggins, 2014). In Heinrich *et al.* (2013), the functionality of the SAC was assessed by measuring the time that cells spend in prometaphase (an early phase in mitosis), after proper chromosome attachment to the mitotic spindle had been experimentally prevented. Prometaphase lengths were determined using fluorescence live-cell microscopy with Plo1-mCherry, whose localization to spindle pole bodies marks prometaphase (Fig. 3). Images were collected every 5 min for at most 17 h, leading to interval and right censoring. Furthermore, cells entered prometaphase at different time points after the start of observation, leading to a distribution of right censoring times.

#### 4.2.1 Inference of SAC subpopulation structure from censored data

We used MEMO in a hypothesis-driven approach to assess the properties of the distributions of prometaphase lengths in strains differing in the expressed amounts of two proteins (Mad2 and Mad3), which are essential for SAC functionality. We modeled interval censored prometaphase lengths as weighted mixtures of two log-normal distributions. Censoring times were described by a Johnson SU distribution.

**Fig. 4.**
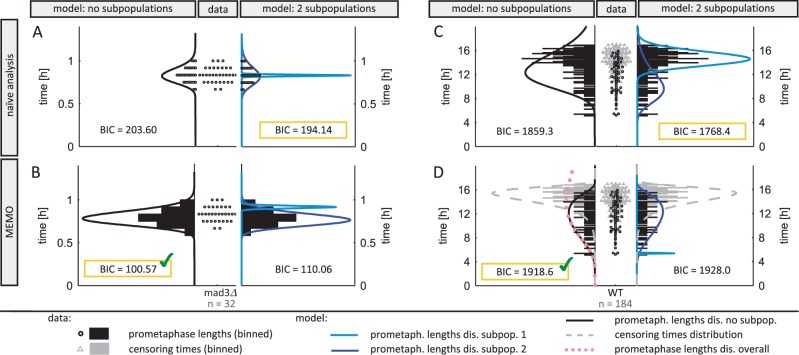
Interval and right censoring has to be considered for accurate reconstruction of SAC functionality from fluorescence live-cell microscopy imaging. Circles and black bars indicate cells in which the entire prometaphase was recorded (interval censored data). Triangles and gray bars indicate cells that were still in prometaphase when recording stopped (right censored data). Left panel: For the *mad3Δ* strain (0% Mad3, dysfunctional SAC) unimodal prometaphase lengths are observed. We used the BIC to decide upon the number of subpopulations for different settings. (**A**) A naïve analysis, disregarding interval censoring, selects a statistical model with two subpopulations, while (**B**) MEMO selects a model with a single population. Right panel: For the wild type (WT) strain a large portion of right censored data are observed. (**C**) The commonly used approach to set prometaphase length of censored data to censoring time selects a statistical model with two subpopulations, while (D) MEMO selects a model with a single subpopulation and Johnson SU distributed censoring times. The pink dotted line depicts the reconstructed overall distribution of prometaphase lengths (i.e. the distribution that would be observed if the observation time was infinite) obtained using MEMO (Color version of this figure is available at *Bioinformatics* online.)

Motivated by the results of the simulation study, we initially compared the performance of MEMO with classical mixture modeling approaches that disregard censoring using data of two different strains. One strain has a completely dysfunctional checkpoint. This is reflected in the dataset by tight unimodal short prometaphase lengths (Fig. 4A and B). The other strain has a functional checkpoint and therefore exhibits much longer prometaphase times, including a large portion of right censored data (Fig. 4C and D). Using the BIC as model selection criterion to determine the number of subpopulations, naïve approaches select a statistical model with a mixture of two log-normal subpopulations for both datasets, while MEMO was able to identify the biologically plausible result of single log-normal populations (Fig. 4).

**Fig. 5.**
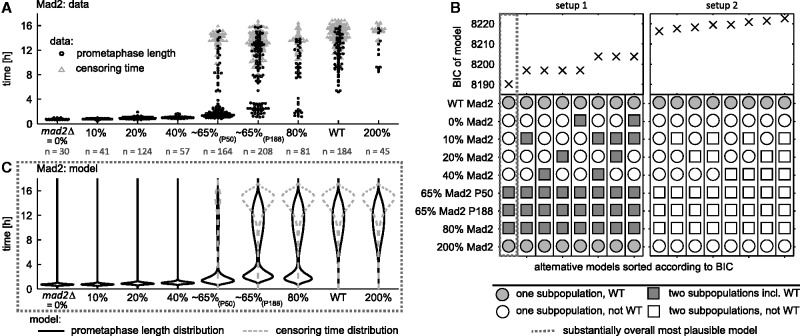
Analysis of subpopulation structure of SAC functionality in different strains using MEMO. (**A**) Measured prometaphase lengths distributions for *S. pombe* strains with different Mad2 abundances. Circles indicate cells in which the entire prometaphase was recorded (prometaphase lengths, interval censored). Triangles indicate cells that were still in prometaphase when recording stopped (censoring times). Since cells enter prometaphase asynchronously, the times at which data are censored are distributed. (**B**) We considered two initial models for the prometaphase lengths distributions: setup 1 - weighted mixture of wild type and strain specific distribution; and setup 2 - weighted mixture of two strain-specific distributions. Starting from these initial models, backward model selection was performed. In each step all possible individual simplifications were performed and the best model was selected. For each setup, the structures of the eight most plausible models are recorded, ranked according to their BIC. According to the most plausible model, the 200% Mad2 strain is indistinguishable from the wild type. 0% Mad2, 10% Mad2, 20% Mad2 and 40% Mad2 strains consist of unimodal distributions significantly different from wild type. Both 65% Mad2 strains and the 80% Mad2 strain consist of two subpopulations. (**C**) Model fit of distributions for prometaphase lengths (black lines) and censoring times (dashed gray lines) for the overall most plausible model selected by MEMO. To mimic the bee swarm plots in subfigure A, probability densities are vertically mirrored

In a second step, we used MEMO to assess the qualitative and quantitative properties of the distributions of prometaphase lengths in the different strains. Experimental data for all strains in which Mad2 abundance was altered are shown in Figure 5A (Mad3 in Supplementary Material, Section S.4, Fig. S12). The recorded prometaphase lengths indicate the presence of cellular subpopulations with functional and dysfunctional SAC for certain strains. Cells with a functional SAC, e.g. wild type cells, have a minimum prometaphase time of at least 5 h, while cells with dysfunctional SAC have shorter prometaphase lengths (Heinrich *et al.*, 2013). For strains with 65% and 80% Mad2 expression, subpopulations with either property seem to be present (Fig. 5A). A single parametrization of the right censoring distribution was used for all strains, as censoring was statistically identical. The parameters of the distributions of prometaphase lengths are estimated along with the subpopulation sizes, i.e. the weights $w_{s}(u)$ of the distributions, and the parameters of the censoring distribution.

The full statistical model possesses many parameters. In order to find the minimal description of the data, we used MEMO to perform backward model selection (Fig. 5B, setup 2). The successive simplification of the initial model resulted in a 33% reduction of the number of parameters. As the subpopulations with functional SAC seemed to possess similar parameters, we considered in the next step a weighted mixture of the wild type prometaphase lengths distribution and a strain-specific distribution, and again performed backward model selection (Fig. 5B, setup 1). By comparing the BIC values for setups 1 and 2, we confirmed that the subpopulations with functional SAC have the statistical properties of the wild type. Furthermore, the existence of two subpopulations could be statistically substantiated for the three suspected strains (Fig. 5B, setup 1, leftmost column). These results were confirmed for different distribution assumptions (Supplementary Material, Section S.4, Fig. S5). Moreover, the parameter uncertainties are small (Supplementary Material, Fig. S10). The selected model (leftmost column in setup 1 of Fig. 5B) quantitatively agrees with the observed experimental data (Fig. 5A and C).

Altogether, MEMO provides results that are consistent with the available knowledge and the apparent structure of the data without being provided with this information. Together with the outcome observed for simulated data, these results indicate that MEMO can robustly extract the subpopulation structure and allows for rigorous hypothesis testing.

#### 4.2.2 Study of SAC regulatory mechanisms using multi-experiment modeling

Its feature to use functional dependencies of the model parameters on the experimental conditions, e.g. $w_{s}=\text{fun}(\theta ,u)$, enables MEMO to compare alternative regulatory mechanisms in addition to the inference of the subpopulation structure. We exploited this feature to study whether the fraction of wild type-like cells is independently affected by Mad2 and Mad3 perturbations or whether these two proteins act synergistically on this fraction.

**Fig. 6.**
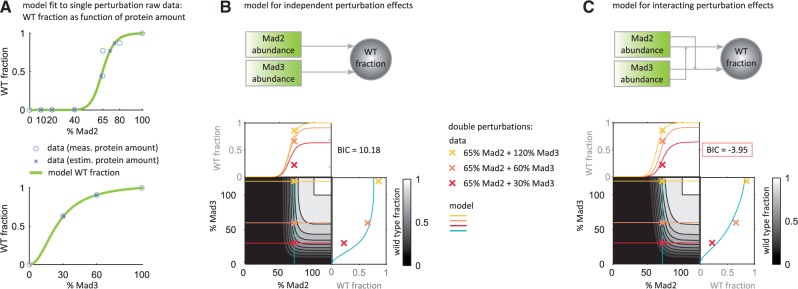
Data-driven hypotheses testing of SAC perturbation response using MEMO. (**A**) A multi-experiment mixture model using a Hill-type description for the fraction of cells with functional SAC was independently fitted to Mad2 and Mad3 perturbations. Errors in the quantification of Mad2 and Mad3 were modeled using normally distributed measurement noise. Circles indicate the data for measured abundances, crosses for the protein abundances that result from estimation. (**B**, **C**) Comparison of model agreement for independent and synergistic effects of Mad2 and Mad3 with the measured fraction of wild type-like cells in strains with double perturbations. Crosses indicate data from three double perturbation strains. Curves show the wild type fraction in cut planes through the Mad2-Mad3 plane as computed from the respective model. The model in (B) reflects independent effects of Mad2 and Mad3 perturbations by just multiplying the models for the Mad2 and Mad3 perturbations from (A). The model in (C) considers interacting effects of perturbations in Mad2 and Mad3 by modeling the threshold parameters in each of the two Hill-type functions to be inversely proportional to the amount of the other protein. As indicated by the lower BIC of the model in C, a synergistic influence of both proteins on the wild type fraction is more likely (Color version of this figure is available at *Bioinformatics* online.)

To assess these competing hypotheses, we compared two models encoding these hypotheses. First we modeled the fraction of cells with a functional (= wild type-like) SAC for different Mad2 and Mad3 abundances using a product of two Hill-type functions (Fig. 6A). The variables of the Hill-type functions were relative Mad2 and relative Mad3 abundance, respectively. To take synergistic effects into account, as proposed by the second hypothesis, in a second model the threshold parameters of these functions were described to be inversely proportional to the amount of the other protein (Supplementary Material, Section S.4.4). To account for uncertainties in protein quantification, deviations from the measured amounts were included as unknown parameters in the parametrization of the model.

Comparing the agreement of the two models with data from three double perturbation strains, 65% Mad2 & 120% Mad3, 65% Mad2 & 60% Mad3 and 65% Mad2 & 30% Mad3 (Fig. 6B and C, respectively; data in Supplementary Material, Fig. S18), the results indicate that a synergistic influence of Mad2 and Mad3 on the fraction of wild type-like cells is more likely. This is consistent with Mad2 and Mad3 acting in the same complex to inhibit Cdc20/Slp1 (Heinrich *et al.*, 2013), thereby inhibiting cell cycle progression. In cases when the underlying signaling network is elusive such data-driven hypothesis testing can give new insights into the signaling mechanism. The use of functional dependencies furthermore allows for predictions on yet unobserved experimental conditions.

### 4.3 Mechanistic parametrization of subpopulation location in NGF-induced Erk phosphorylation

The ability of MEMO to incorporate functional dependencies of the mixture parameters on the experimental conditions facilitates the use of prior knowledge on the population and pathways structure. In combination with the possibility to study multiple experimental conditions - time points *t* and stimulations *u* - simultaneously, MEMO enables mechanistic modeling of single-cell data. For a realistic assessment of these features, we considered NFG-induced Erk phosphorylation in primary sensory neurons. Quantitative single-cell data for Erk phosphorylation and models for the population dynamics have been presented by Hasenauer *et al.* (2014b).

**Fig. 7.**
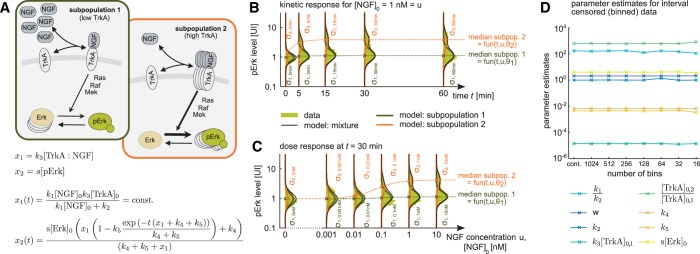
Mechanistic parametrization of the median of each subpopulation emerging in NGF induced Erk phosphorylation. (**A**) Schematic of the model for NGF-induced Erk1/2 signaling as presented in Hasenauer *et al.* (2014b). Arrows represent conversion reactions and regulatory interactions. The abundance of the proteins is illustrated by its frequency in the schematic. By defining *x*_1_ and *x*_2_ and assuming $x_{1}(t)$ to be in quasi steady state, $x_{2}(t)$ can be used to describe the dependency of the subpopulation medians on time and stimulus strength. (**B**) Model fit of kinetic response data. Data are shown as histograms. (**C**) Model fit of dose response data. Data are shown as histograms. Vectors θ_1_ and θ_2_ contain all unknown parameters and only differ in containing $k_{3}{\left[\text{TrkA}\right]}_{0,1}$ and $k_{3}{\left[\text{TrkA}\right]}_{0,2}$, respectively. (**D**) Parameter estimates based on original data (cont.) and artificially interval censored (binned) data for different numbers of bins in data range (Color version of this figure is available at *Bioinformatics* online.)

We employed MEMO to reevaluate the best model found by Hasenauer *et al.* (2014b). This model accounts for two subpopulations differing in the total TrkA concentration, ${\left[\text{TrkA}\right]}_{\text{0}}$ (Fig. 7A). The phospho-Erk concentrations in the subpopulations are assumed to be log-normally distributed. The medians of these log-normal distributions are modeled by ordinary differential equations describing the biochemical reaction network, i.e. binding of NGF to its receptor TrkA and subsequent Erk phosphorylation. The state variables are the activity of the receptor TrkA, $x_{1}=k_{3}[\text{TrkA}:\text{NGF}]$, and the scaled abundance of phospho-Erk, $x_{2}=s[\text{pErk}]$, and the system input is the NGF concentration, $u={\left[\text{NGF}\right]}_{\text{0}}$. Employing that the receptor dynamics are fast, we derived analytical solutions for $x_{1}(t,u)$ and $x_{2}(t,u)$ (Fig. 7A). The analytical solution of $x_{2}(t,u)$ is used as parametric description of the subpopulation medians. The parameters of the population model are the kinetic parameters of the biochemical reaction network, effective subpopulation specific receptor concentrations, the scale parameters of the log-normal distributions and the subpopulation size (see Supplementary Material, Section S.5).

The multi-experiment modeling of the Erk phosphorylation data using MEMO enabled the quantitative description of the population dynamics as well as the tracking of the subpopulations across time points (Fig. 7B) and NGF concentration (Fig. 7C). To assess the robustness of parameter estimates with respect to censoring, the uncensored data as well as (artificially) interval censored data were considered. We found that the appropriate consideration of intervals censoring allows for the robust estimation of kinetic parameters for a wide-range of bin numbers (Fig. 7D). Hence, censored data allow for the quantitative assessment of kinetic parameters.

## 5 Discussion

Mixture modeling of single-cell data is receiving increasing attention due to a rising number of single-cell technologies (Buettner *et al.*, 2015; Crane *et al.*, 2014; Grün *et al.*, 2015; Hoppe *et al.*, 2014). Standard approaches are well established (Pyne *et al.*, 2009; Wang and Huang, 2007), and several software packages are available. FLAME (Pyne *et al.*, 2009) and flowClust (Lo *et al.*, 2008) enable Gaussian, t and skew t mixture modeling of flow cytometry data and can also be used for other data types. In these implementations, multiple samples are handled using metaclustering or distribution matching. JCM (Pyne *et al.*, 2014) improves upon that by constructing template models and matching the individual samples to the template models. This approach even allows for the consideration of inter-sample variability, at least in the matching step. However, JCM does not facilitate an automatic matching of subpopulations across different experimental conditions, and - like all other methods - does currently not incorporate hypothesis testing methods. Furthermore, these packages do not account for censoring, which might lead to misinterpretations (Duffy *et al.*, 2012; Kuchina *et al.*, 2011). In this study we show that disregarding censoring generally results in an overestimation of the number of subpopulations. In case of interval censoring the severity depends on the ratio of censoring interval and inter-cell variability (Fig. 1B).

Multi-experiment mixture modeling using MEMO enables accurate reconstruction of subpopulation structures and properties from interval, left and right censored data. Inter-sample variability can be modeled using sample-specific scaling and offset parameters. By simultaneously analyzing multiple experiments, MEMO facilitates the comparison of different regulatory mechanisms. Thus, mixture modeling is no longer restricted to data analysis, but also allows to formulate and compare hypotheses how subpopulations are linked across different experimental conditions. As demonstrated, MEMO can also be combined with mechanistic modeling approaches. Furthermore, the characterization of experiment-dependent subpopulations is improved, since variability quantification is enriched by the entire dataset from multiple experiments.

The results of MEMO can be used for subsequent modeling (Heinrich *et al.*, 2013; Song *et al.*, 2010). Applications to a broad range of data types, e.g. single-cell time-lapse (Section 4.2), and single-cell protein level snapshot data (Section 4.3) are possible. The approach can also be used in medical studies, where patients are not observed continuously or may drop out from the study.

The current implementation of MEMO supports analytical functions to link experimental conditions. These functions encode hypotheses and can be derived from measurement data or mechanistic models, such as for example ordinary differential equation (ODE) constrained mixture models (ODE-MM) as described in Hasenauer *et al.* (2014b). ODE-MMs use mechanistic models of single cell behavior and subpopulation structure to integrate data collected under different experimental conditions (Hasenauer *et al.*, 2014a; Thomas *et al.*, 2014), and could be used to reconstruct differences between subpopulations. MEMO provides an extension to ODE-MM as censored data can be studied and knowledge about the signaling pathway is not required. This renders MEMO more flexible and easier to use for explorative data analysis. The mechanistic modeling of single-cell snapshot data using MEMO demonstrated its flexibility concerning the data types and established that causal relations can be extracted. Furthermore, it revealed that parameter estimates using different data resolutions are consistent.

For the inference of model parameters, MEMO uses a maximum-likelihood method along with efficient gradient-based optimization. Expectation maximization (EM) algorithms suitable for multi-experiment mixture models with censored data could reduce the computation time further and add to the robustness. Existing EM methods for censored data (Lee and Scott, 2012) will have to be extended to a multi-experiment setting. Model selection criteria implemented in MEMO could be complemented by deviance information criteria and Bayesian model selection (Steele and Raftery, 2010).

MEMO is currently restricted to the analysis of censored and uncensored univariate data. An extension of MEMO to truncated and multivariate data is possible, the latter poses however several challenges. Among other, the evaluation of the likelihood for censored data requires the calculation of multivariate integrals (McLachlan and Jones, 1988), which is already computationally intensive for the bivariate case (Cadez *et al.*, 2002). Potential solutions might be provided by sparse grids (see (Burkardt, 2014) and references therein). As the analysis of multivariate data is currently not possible with MEMO, MEMO needs to be combined with preprocessing and dimension reduction approaches (Angerer *et al.*, 2016). Moreover, depending on the experimental setup, prior to analysis the data may have to be corrected for experimental biases that mask the biological population structure (Buettner *et al.*, 2015). To facilitate the biological interpretation of the results, a hierarchical view on cell populations should be incorporated in MEMO (Usoskin *et al.*, 2015).

We also note that a full exploitation of the possibilities of MEMO requires some expertise in the biological system. This concerns the choice of the number of subpopulations to start with and in particular the generation of hypotheses on the relations between different experimental conditions. Furthermore, the user must provide reasonable parameter bounds for the optimization procedure. In this sense MEMO is an advanced tool that is not fully automatic. Nevertheless, its modular concept and the symbolic programming make MEMO’s basic functionality intuitively accessible.

In summary, we introduced a computational method for the efficient and integrated analysis of censored data that allows for rigorous hypothesis testing. The implementation of this method, MEMO, can facilitate the coherent and reliable analysis of single-cell data across experimental platforms. Such standardized analysis pipelines are essential in the age of single-cell data (Pelkmans, 2012).

## Supplementary Material

Supplementary Data
