# Supplementary material for: MEMO: multi-experiment mixture model analysis of censored data
Source: Bioinformatics. 2016 Apr 19;32(16):2464–72. doi: 10.1093/bioinformatics/btw190 (PMC4978932; doi:10.1093/bioinformatics/btw190)
Supplement: Supplementary Data [file supp_32_16_2464__index.html]

MEMO - Multi-experiment mixture model analysis of censored data — MEMO: multi-experiment mixture model analysis of censored data — MEMO: multi-experiment mixture model analysis of censored data — Supplementary Data 

# MEMO: multi-experiment mixture model analysis of censored data

## Supplementary Data

files

- Supplementary Data - pdf file
- Supplementary Data - pdf file
