# Supplementary material for: MEMO: multi-experiment mixture model analysis of censored data
Source: Bioinformatics. 2016 Apr 19;32(16):2464–72. doi: 10.1093/bioinformatics/btw190 (PMC4978932; doi:10.1093/bioinformatics/btw190)
Supplement: Supplementary Data [file supp_btw190_MEMO_Documentation.pdf]

Documentation:
MEMO - Multi-experiment mixture model analysis of censored data

E.-M. Geissen, J. Hasenauer, S. Heinrich, S. Hauf, F.J. Theis and N. Radde

Contents

D.1 Introduction **2**

D.2 Methods **3**

D.2.1 Statistical modeling of uncensored and censored data . . . 3
D.2.1.1 Distribution of data in the absence of censoring . . . 3
D.2.1.2 Distribution of data in the presence of interval censoring . . . 3
D.2.1.3 Distribution of data in the presence of right censoring . . . 4
D.2.1.4 Distribution of data in the presence of left censoring . . . 5
D.2.1.5 Distribution of data in the presence of interval and right censoring . . . 6
D.2.2 Step 1: Formulation of hypotheses . . . 7
D.2.3 Step 2: Parameterization of multi-experiment mixture models . . . 9
D.2.3.1 Frequentist parameter estimation . . . 9
D.2.3.2 Bayesian parameter estimation . . . 10
D.2.4 Step 3: Testing of hypotheses via model selection . . . 11
D.2.4.1 Model selection methods . . . 11
D.2.4.2 Automated unraveling of subpopulation structure . . . 12
D.2.5 Step 4: Interpretation and further analysis . . . 12
D.2.6 Gradients of objective function . . . 13
D.2.6.1 Gradient of the log-likelihood function . . . 13
D.2.6.2 Gradient of the mixture distribution . . . 14
D.2.6.3 Gradient of the probability densities . . . 15

D.3 Implementation of MEMO **16**

D.3.1 Requirements . . . 16
D.3.2 Model formulation and data format . . . 16
D.3.3 Likelihood calculation . . . 16
D.3.4 Optimization . . . 16
D.3.5 Automated backward model selection . . . 18
D.3.6 Goodness of fit analysis . . . 19
D.3.7 Uncertainty analysis . . . 19
D.3.8 Visualization . . . 19

D.1 Introduction

MEMO is an easy to use software toolbox for the statistical analysis of uncensored and censored single-cell data. It can deal with a variety of data types. The subpopulation structure and statistical properties of heterogeneous cell populations are assessed by the application of maximum likelihood inference and hypothesis testing to mixture models. MEMO 's key features are

1. support of uncensored, interval, left and right censored data,
2. symbolic definition and compilation of the mixture models,
3. support of normal, log-normal, gamma and Johnson-SU distributions,
4. integration and simultaneous analysis of multiple experimental conditions,
5. provision of gradient information for multi-start local optimization,
6. automated backward model selection,
7. integration of identifiability and uncertainty analysis (profile likelihoods and Markov chain Monte Carlo methods) using PESTO.

Requirements

MEMO is a software package for MATLAB. To exploit its functionality the following toolboxes are required:

- Optimization Toolbox (http://www.mathworks.com/products/optimization/)
- Symbolic Math Toolbox (http://www.mathworks.com/products/symbolic/)
- Statistics and Machine Learning Toolbox (http://www.mathworks.com/products/statistics/)

In addition, for parameter estimation and MCMC sampling the toolboxes

- PESTO (http://www.helmholtz-muenchen.de/icb/research/groups/data-driven-computational- modelling/staff/ma/2519/index.html)
- DRAM (http://helios.fmi.fi/ lainema/mcmc/)

are required.

D.2 Methods

D.2.1 Statistical modeling of uncensored and censored data

We consider the inference of multi-experiment mixture models $\mathcal{M}$,

$$p(x|\theta, u) = \sum_{s=1}^{S} w_s(u)\phi(x|\varphi_s(u)), \tag{1}$$

from uncensored and censored data $\mathcal{D}$. The mixture model describes the distribution of the quantity of interest X in a heterogeneous population consisting of subpopulations $s = 1, \dots, S$ with relative subpopulation sizes $w_s(u)$ and subpopulation mixture parameters $\varphi_s(u)$ for experimental condition u. The properties of the individual subpopulations are described by the probability densities $\phi(x|\varphi_s(u))$. The sum of the relative subpopulation sizes is one, $\sum_{s=1}^{S} w_s(u) = 1$. Subpopulation sizes and mixture parameters can depend on the experimental conditions u. To infer these functional dependencies multiple experimental conditions u_i, $i = 1, \dots, I$, are considered.

For the inference of the mixture model, uncensored data x, right or left censored data $\overline{x}$ or $\underline{x}$, and interval censored data $\underline{\overline{x}}$ are considered. The overall dataset $\mathcal{D}$ is a collection of the datasets $\mathcal{D}_i$ for individual experimental conditions u_i,

$$\mathcal{D} = \{\mathcal{D}_i\}_{i=1}^{I} = \left\{ \left(\left\{ x_i^j \right\}_{j=1}^{n_{x,i}}, \{\overline{x}_i^k\}_{k=1}^{n_{\overline{x},i}}, \{\underline{\overline{x}}_i^l\}_{l=1}^{n_{\underline{\overline{x}},i}}, \{\underline{x}_i^m\}_{m=1}^{n_{\underline{x},i}} \right) \right\}_{i=1}^{I}.$$

We denote the j-th uncensored, the k-th right censored, the l-th interval censored, and the m-th left-censored measurement under experimental condition u_i by x_i^j, $\overline{x}_i^k$, $\underline{\overline{x}}_i^l$, and $\underline{x}_i^m$, respectively. The number of data points in experimental condition u_i are $n_{x,i}$, $n_{\overline{x},i}$, $n_{\underline{\overline{x}},i}$ and $n_{\underline{x},i}$. In practice, not all four data types are available at the same time but there are four common combinations: (i) uncensored data; (ii) uncensored and right or left censored data; (iii) interval censored data; and (iv) interval and right or left censored data. In the following, we will introduce statistical models for the process of data collection in the presence of censoring. In this process data is generated from a data generating distribution, the distribution we are actually interested in. Due to censoring this distribution differs from distribution of observed data. By modeling these changes we will derive the likelihood function for a single experimental condition u_i.

D.2.1.1 Distribution of data in the absence of censoring

For independent identically distributed (i.i.d.) measurements, uncensored data $\left\{ x_i^j \right\}$ provide samples from $p(x_i|\theta, u_i)$. Accordingly, for experimental condition u_i the dataset consists of samples

$$X_i \sim p(x_i|\theta, u_i).$$

Uncensored measurements provide information about the full distribution, enabling reliable reconstruction of the distribution $p(x_i|\theta, u_i)$ for sufficiently large sample numbers $n_{x,i}$. The reconstruction of the distribution does not ensure that the parameters θ are identifiable. For mixture models, for instance, the problem of symmetry is well-known.

In the absence of censoring, the likelihood function for data $\mathcal{D}_i$ is given by

$$\mathbb{P}(\mathcal{D}_i|\theta) = \prod_{j=1}^{n_{x,i}} p(x_i^j|\theta, u_i).$$

D.2.1.2 Distribution of data in the presence of interval censoring

Interval censored data $\{\underline{\overline{x}}_i^l\}$ provide the information that the corresponding uncensored data point x_i lies in the interval $(\underline{\overline{x}}_i^l - \Delta x, \underline{\overline{x}}_i^l]$. The interval length is denoted by Δx. Accordingly, for experimental condition u_i the dataset consists of samples

$$\begin{aligned}\underline{\overline{X}}_i \sim \underline{\overline{p}}(\underline{\overline{x}}_i|\theta, u_i) \quad \text{with} \quad \underline{\overline{p}}(\underline{\overline{x}}_i|\theta, u_i) &= \int_{\underline{\overline{x}}_i - \Delta x}^{\underline{\overline{x}}_i} p(x_i|\theta, u_i) dx_i \\ &= P(\underline{\overline{x}}_i|\theta, u_i) - P(\underline{\overline{x}}_i - \Delta x|\theta, u_i)\end{aligned}$$

with cumulative distribution $P(x_i|\theta, u_i) := \int_{-\infty}^{x_i} p(x_i'|\theta, u_i)dx_i'$. Interval censored data provide information about the probability mass between two observation points. The precise shape of the probability density between observation points cannot be reconstructed but is merely restricted by the distribution assumption.

In the presence of interval censoring, the likelihood function for data $\mathcal{D}_i$ is

$$\mathbb{P}(\mathcal{D}_i|\theta) = \prod_{l=1}^{n_{\overline{\underline{x}},i}} \underline{\overline{p}}(\overline{\underline{x}}_i^l|\theta, u_i).$$

Note that we assume that the length of all intervals is identical. This can easily be generalized. Furthermore, instead of the endpoint $\overline{\underline{x}}_i^j$ of an interval also a description in terms of an interval index, e.g. k for an interval $((k-1)\Delta x, k\Delta x]$, might be used. By rewriting the likelihood in terms of observations per interval, the link to the multinomial distribution becomes apparent.

D.2.1.3 Distribution of data in the presence of right censoring

In the presence of right censoring, uncensored data $\left\{x_i^j\right\}$ and right censored data $\left\{\overline{x}_i^k\right\}$ are observed. In order to model the data collection process, we define the indices j, k, and z. These indices enumerate the uncensored and right censored datapoints and the sum of both, respectively, and are initially set to 0. For each data point z, sample pairs x_i and $x_{i,\overline{c}}$ are drawn from their respective distributions,

$$X_i \sim p(x_i|\theta, u_i), X_{i,\overline{c}} \sim p_c(x_{i,\overline{c}}|\theta, u_i),$$

where $p_c(x_{i,\overline{c}}|\theta, u_i)$ denotes the right censoring distribution. Then we set

$$\begin{aligned} x_i^z &= x_i \\ x_{i,\overline{c}}^z &= x_{i,\overline{c}} \\ z &= z + 1. \end{aligned}$$

The minimum of these two random variables is recorded and added to the dataset, i.e.

$$\begin{aligned} &\text{If } x_i^z \le x_{i,\overline{c}}^z && \Rightarrow \text{ set } x_i^j = x_i^z \text{ and } j = j+1 \\ &\text{If } x_{i,\overline{c}}^z < x_i^z && \Rightarrow \text{ set } \overline{x}_i^k = x_{i,\overline{c}}^z \text{ and } k = k+1. \end{aligned}$$

The distributions of observed uncensored and right censored data for experimental condition u_i are the conditional distributions

$$\begin{aligned} p(x_i|x_i \le X_{i,\overline{c}}, \theta, u_i) &= \frac{p(x_i, x_i \le X_{i,\overline{c}}|\theta, u_i)}{p(X_i \le X_{i,\overline{c}}|\theta, u_i)}, \\ p(x_{i,\overline{c}}|x_{i,\overline{c}} < X_i, \theta, u_i) &= \frac{p(x_{i,\overline{c}}, x_{i,\overline{c}} \le X_i|\theta, u_i)}{p(X_{i,\overline{c}} \le X_i|\theta, u_i)}, \end{aligned}$$

with joint distributions

$$\begin{aligned} p(x_i, x_i \le X_{i,\overline{c}}|\theta, u_i) &= p(x_i|\theta, u_i)(1 - P_c(x_i|\theta, u_i)), \\ p(x_{i,\overline{c}}, x_{i,\overline{c}} \le X_i|\theta, u_i) &= p_c(x_{i,\overline{c}}|\theta, u_i)(1 - P(x_{i,\overline{c}}|\theta, u_i)), \end{aligned}$$

and marginal probabilities for observing uncensored or censored data,

$$\begin{aligned} p(X_i \le X_{i,\overline{c}}|\theta, u_i) &= \int_{-\infty}^{\infty} p(x_i|\theta, u_i)(1 - P_c(x_i|\theta, u_i))dx_i, \\ p(X_{i,\overline{c}} \le X_i|\theta, u_i) &= \int_{-\infty}^{\infty} p_c(x_{i,\overline{c}}|\theta, u_i)(1 - P(x_{i,\overline{c}}|\theta, u_i))dx_{i,\overline{c}}. \end{aligned}$$

The cumulative distributions of X_i and $X_{i,\overline{c}}$ are denoted by $P(x_i|\theta, u_i)$ and $P_c(X_{i,\overline{c}}|\theta, u_i)$, respectively. As analytical solutions of $p(X_i \le X_{i,\overline{c}}|\theta, u_i)$ and $p(x_{i,\overline{c}} \le X_i|\theta, u_i)$ are often not available, numerical integration might be necessary.

The right censoring distribution can have different shapes. In the case of distributed censoring, meaning that $p_c(x_{i,\overline{c}}|\theta, u_i)$ is a smooth distribution, the likelihood function for data $\mathcal{D}_i$ is proportional to

$$\begin{aligned}\mathbb{P}(\mathcal{D}_i|\theta) &\propto \left(\prod_{j=1}^{n_{x,i}} p(x_i^j, x_i^j \leq X_{i,\overline{c}}|\theta, u_i)\right)\left(\prod_{k=1}^{n_{\overline{x},i}} p(\overline{x}_i^k, \overline{x}_i^k \leq X_i|\theta, u_i)\right) \\ &\propto \left(\prod_{j=1}^{n_{x,i}} p(x_i^j|\theta, u_i)(1 - P_c(x_i^j|\theta, u_i))\right)\left(\prod_{k=1}^{n_{\overline{x},i}} p_c(\overline{x}_i^k|\theta, u_i)(1 - P(\overline{x}_i^k|\theta, u_i))\right).\end{aligned}$$

In the case of fixed censoring at a single value $\tilde{x}_{i,\overline{c}}$, translating into a probability density which is a Dirac delta, $p_c(x_{i,\overline{c}}|\theta, u_i) = \delta(x_{i,\overline{c}} - \tilde{x}_{i,\overline{c}})$, the likelihood function simplifies and we obtain

$$\mathbb{P}(\mathcal{D}_i|\theta) \propto \left(\prod_{j=1}^{n_{x,i}} p(x_i^j|\theta, u_i)\right)\left(\prod_{k=1}^{n_{\overline{x},i}} (1 - P(\overline{x}_i^k|\theta, u_i))\right).$$

This formulation exploits the tail probabilities $1 - P(\overline{x}_i^k|\theta, u_i)$, capturing the censoring. Sometimes this likelihood function is also used to avoid modeling of the censoring. While this still allows for inference, a visual comparison of model and data requires an estimate of the censoring distribution (see Section S.4.2.1, Model-data comparison in the Supplementary Material), since the conditional distributions above have to be evaluated for this purpose. Furthermore, this estimate is required for goodness-of-fit analysis based on bootstrapping the likelihood distribution of the objective function (see Section S.4.2.1, Goodness of fit in the Supplementary Material). Without an estimate of the censoring distribution, the expected event and censoring distribution – $p(x_i, x_i \leq X_{i,\overline{c}}|\theta, u_i)$ and $p(x_{i,\overline{c}}, x_{i,\overline{c}} \leq X_i|\theta, u_i)$ – cannot be predicted, and therefore it is not possible to resample data for a bootstrapping.

D.2.1.4 Distribution of data in the presence of left censoring

In the presence of left censoring, uncensored data $\left\{x_i^j\right\}$ and left censored data $\{\underline{x}_i^m\}$ are observed. In order to model the data collection process in the presence of left censoring, we proceed similarly to the case of right censoring, i. e. sample pairs x_i and $x_{i,\underline{c}}$ are drawn from their respective distributions,

$$X_i \sim p(x_i|\theta, u_i), X_{i,\underline{c}} \sim p_l(x_{i,\underline{c}}|\theta, u_i),$$

where $p_l(x_{i,\underline{c}}|\theta, u_i)$ denotes the left censoring distribution. Counters are updated acccordingly. The maximum of these two random variables is recorded and added to the dataset,

$$\begin{aligned}&\text{If } x_i^z \geq x_{i,\underline{c}}^z &&\Rightarrow \text{ set } x_i^j = x_i^z \text{ and } j = j+1 \\ &\text{If } x_{i,\underline{c}}^z > x_i^z &&\Rightarrow \text{ set } \underline{x}_i^m = x_{i,\underline{c}}^z \text{ and } m = m+1.\end{aligned}$$

The distributions of observed uncensored and left censored data are

$$\begin{aligned}p(x_i|x_i \geq X_{i,\underline{c}}, \theta, u_i) &= \frac{p(x_i, x_i \geq X_{i,\underline{c}}|\theta, u_i)}{p(X_i \geq X_{i,\underline{c}}|\theta, u_i)}, \\ p(x_{i,\underline{c}}|x_{i,\underline{c}} > X_i, \theta, u_i) &= \frac{p(x_{i,\underline{c}}, x_{i,\underline{c}} > X_i|\theta, u_i)}{p(X_{i,\underline{c}} > X_i|\theta, u_i)},\end{aligned}$$

with joint distributions

$$\begin{aligned}p(x_i, x_i \geq X_{i,\underline{c}}|\theta, u_i) &= p(x_i|\theta, u_i)P_l(x_i|\theta, u_i), \\ p(x_{i,\underline{c}}, x_{i,\underline{c}} > X_i|\theta, u_i) &= p_l(x_{i,\underline{c}}|\theta, u_i)P(x_{i,\underline{c}}|\theta, u_i),\end{aligned}$$

and marginal probabilities for observing uncensored or censored data,

$$\begin{aligned}p(X_i \geq X_{i,\underline{c}}|\theta, u_i) &= \int_{-\infty}^{\infty} p(x_i|\theta, u_i)P_l(x_i|\theta, u_i)dx_i, \\ p(X_{i,\underline{c}} > X_i|\theta, u_i) &= \int_{-\infty}^{\infty} p_l(x_{i,\underline{c}}|\theta, u_i)P(x_{i,\underline{c}}|\theta, u_i)dx_{i,\underline{c}}.\end{aligned}$$

The cumulative distributions of X_i and $X_{i,\underline{c}}$ are denoted by $P(x_i|\theta, u_i)$ and $P_l(x_{i,\underline{c}}|\theta, u_i)$, respectively.

The likelihood function for data $\mathcal{D}_i$ is proportional to

$$\begin{aligned}\mathbb{P}(\mathcal{D}_i|\theta) &\propto \left(\prod_{j=1}^{n_{x,i}} p(x_i^j, x_i^j \geq X_{i,\underline{c}}|\theta, u_i)\right)\left(\prod_{m=1}^{n_{\underline{x},i}} p(\underline{x}_i^m, \underline{x}_i^m > X_i|\theta, u_i)\right)\\ &\propto \left(\prod_{j=1}^{n_{x,i}} p(x_i^j|\theta, u_i)P_l(x_i^j|\theta, u_i)\right)\left(\prod_{m=1}^{n_{\underline{x},i}} p_l(\underline{x}_i^m|\theta, u_i)P(\underline{x}_i^m|\theta, u_i)\right)\end{aligned}$$

and can be simplified acccordingly in case of a delta distribution for the left censoring times.

D.2.1.5 Distribution of data in the presence of interval and right censoring

In the presence of interval and right censoring, interval censored data $\{\overline{\underline{x}}_i^l\}$ and right censored data $\{\overline{x}_i^k\}$ are observed. In order to model the data collection process in the presence of interval and right censoring, all counters are initially set to 0, i. e. $k, l, z = 0$, and sample pairs x_i and $x_{i,\bar{c}}$ are drawn from their respective distributions,

$$X_i \sim p(x_i|\theta, u_i), X_{i,\bar{c}} \sim p_c(x_{i,\bar{c}}|\theta, u_i).$$

Then we set

$$\begin{aligned}x_i^z &= x_i\\ x_{i,\bar{c}}^z &= x_{i,\bar{c}}\\ z &= z+1.\end{aligned}$$

Furthermore,

$$x_i^{+,z} := \mathrm{ceil}_{\Delta x}(x_i^z),\ x_{i,\bar{c}}^{-,z} := \mathrm{floor}_{\Delta x}(x_{i,\bar{c}}^z).$$

The expressions $\mathrm{floor}_{\Delta x}(x)$ and $\mathrm{ceil}_{\Delta x}(x)$ indicate round up and round down of x to the next multiple of Δx. Accordingly, $\mathrm{ceil}_{\Delta x}(x)$ yields the smallest multiple of Δx, x^+, which is larger than x, and $\mathrm{floor}_{\Delta x}(x_{\bar{c}})$ yields the largest multiple of Δx, $x_{\bar{c}}^-$, which is smaller than $x_{\bar{c}}$. Without loss of generality we assume that measured time points are multiples of Δx, such that $\forall i, j \exists k', k''$ such that $\overline{\underline{x}}_i^j = k'\Delta x$ and $\overline{x}_i^j = k''\Delta x$.

The dataset is filled as follows

$$\begin{aligned}&\text{If } x_i^{+,z} \leq x_{i,\bar{c}}^z \Rightarrow \text{ set } \overline{\underline{x}}_i^l = x_i^{+,z} \text{ and } l = l+1\\ &\text{If } x_{i,\bar{c}}^z < x_i^{+,z} \Rightarrow \text{ set } \overline{x}_i^k = x_{i,\bar{c}}^{-,z} \text{ and } k = k+1.\end{aligned}$$

The distributions of interval and right censored data for experimental condition u_i are

$$\begin{aligned}p(x_i^+|x_i^+ \leq X_{i,\bar{c}}, \theta, u_i) &= \frac{p(x_i^+, x_i^+ \leq X_{i,\bar{c}}|\theta, u_i)}{p(X_i^+ \leq x_{i,\bar{c}}|\theta, u_i)},\\ p(x_{i,\bar{c}}^-|x_{i,\bar{c}} < X_i^+, \theta, u_i) &= \frac{p(x_{i,\bar{c}}^-, x_{i,\bar{c}} \leq X_i^+|\theta, u_i)}{p(x_{i,\bar{c}} \leq X_i^+|\theta, u_i)},\end{aligned}$$

with joint distributions

$$\begin{aligned}p(x_i^+, x_i^+ \leq X_{i,\bar{c}}|\theta, u_i) &= \left(P(x_i^+|\theta, u_i) - P(x_i^+ - \Delta x|\theta, u_i)\right)(1 - P_c(x_i^+|\theta, u_i)),\\ p(x_{i,\bar{c}}^-, x_{i,\bar{c}} \leq X_i^+|\theta, u_i) &= \left(P_c(x_{i,\bar{c}}^-|\theta, u_i) - P_c(x_{i,\bar{c}}^- - \Delta x|\theta, u_i)\right)(1 - P(x_{i,\bar{c}}^-|\theta, u_i)),\end{aligned}$$

and marginal probabilities for observing uncensored or censored data,

$$\begin{aligned}p(X_i^+ \leq X_{i,\bar{c}}|\theta, u_i) &= \sum_{k' \in \mathbb{Z}} \left(P(k'\Delta x|\theta, u_i) - P((k'-1)\Delta x|\theta, u_i)\right)(1 - P_c(k'\Delta x|\theta, u_i)),\\ p(X_{i,\bar{c}} \leq X_i^+|\theta, u_i) &= \sum_{k' \in \mathbb{Z}} \left(P_c(k''\Delta x|\theta, u_i) - P_c((k'-1)\Delta x|\theta, u_i)\right)(1 - P(k''\Delta x|\theta, u_i)).\end{aligned}$$

The cumulative distributions of X_i and $X_{i,\bar{c}}$ are denoted by $P(x_i|\theta, u_i)$ and $P_c(x_{i,\bar{c}}|\theta, u_i)$, respectively.

This statistical model of the data in the presence of interval and right censoring models (time-lapse) microscopy experiments as well as flow cytometry experiments (with finite acquisition accuracy). The aspect of censoring is however neglected in standard analysis tools (O'Neill et al., 2013, Pyne et al., 2009).

In the case of distributed censoring the likelihood function for data $\mathcal{D}_i$ is

$$\mathbb{P}(\mathcal{D}_i|\theta) \propto \left(\prod_{l=1}^{n_{\underline{x},i}} p(\underline{x}_i^l, \underline{x}_i^l \leq X_{i,\bar{c}}, \theta, u_i)\right)\left(\prod_{k=1}^{n_{\overline{x},i}} p(\overline{x}_i^k, \overline{x}_i^k \leq X_i^+, \theta, u_i)\right).$$

The joint distribution is used, as we know the value of the respective measurement as well as the order of the occurence of event and censoring. In the case of fixed censoring at a point $\tilde{x}_c$, the likelihood function simplifies and we obtain

$$\mathbb{P}(\mathcal{D}_i|\theta) \propto \left(\prod_{l=1}^{n_{\underline{x},i}} \left(P(\underline{x}_i^l|\theta, u_i) - P(\underline{x}_i^l - \Delta x|\theta, u_i)\right)\right)\left(\prod_{k=1}^{n_{\overline{x},i}} (1 - P(\overline{x}_i^k|\theta, u_i))\right).$$

The case of combining interval and left censoring can be derived accordingly.

In summary, statistical models and likelihood functions for the different types of experimental data have been introduced in Section D.2.1.1 - D.2.1.5. Based on these preliminaries, Multi-Experiment Mixture Modelling using the MATLAB toolbox MEMO will be introduced. MEMO provides a framework for the simultaneous analysis of multiple experiments for which censored and uncensored data are available.

D.2.2 Step 1: Formulation of hypotheses

Standard mixture modeling is commonly used for the quantification of experimental data as well as for the analysis of the subpopulation structures and properties (Pyne et al., 2009). These are inference and hypothesis testing problems. As MEMO allows for the simultaneous study of multiple experimental datasets, more general hypotheses can be assessed.

Hypotheses regarding the condition-dependent population structures can be encoded in the functional dependency of the subpopulation sizes $w_s(u)$ and distribution parameters $\varphi_s(u)$ on the experimental condition u and the parameters θ. These dependencies are generally unknown and can be inferred by MEMO. Parametrization of $w_s(u)$ and $\varphi_s(u)$ with meta-parameters θ is possible. MEMO allows for a flexible symbolic definition of these parameter dependent functions, $w_s(u) = \mathrm{fun}(u, \theta)$ and $\varphi_s(u) = \mathrm{fun}(u, \theta)$, and the inference of the corresponding meta-parameters θ.

The interpretation of the distribution parameters $\varphi_s(u)$ depends on the distribution assumption. In the current version of MEMO four distributions for the subpopulations are supported:

- **Normal distribution:** With distribution parameters $\varphi = (\mu, \sigma)$ the probability density function (pdf) and cumulative distribution function (cdf) are

  $$\phi(x|\varphi) = \frac{1}{\sqrt{2\pi}\sigma} \exp\left(-\frac{1}{2}\left(\frac{x-\mu}{\sigma}\right)^2\right),$$

  and

  $$\Phi(x|\varphi) = \frac{1}{\sqrt{2\pi}\sigma} \int_{-\infty}^{x} \exp\left(-\frac{1}{2}\left(\frac{\tau-\mu}{\sigma}\right)^2\right) d\tau = \Lambda\left(\frac{x-\mu}{\sigma}\right).$$

  With $\Lambda(y)$ we denote the cdf of the standard normal distribution $\mathcal{N}(0,1)$ throughout this documentation.

- **Log-normal distribution:** The pdf and cdf are

  $$\phi(x|\varphi) = \begin{cases} \frac{1}{\sqrt{2\pi}\sigma x} \exp\left(-\frac{1}{2}\left(\frac{\log(x)-\mu}{\sigma}\right)^2\right) & \text{for } x > 0 \\ 0 & \text{otherwise,} \end{cases}$$

and

$$\Phi(x|\varphi) = \frac{1}{\sqrt{2\pi}\sigma} \int_0^x \frac{1}{\tau} \exp\left(-\frac{1}{2}\left(\frac{\log(\tau)-\mu}{\sigma}\right)^2\right) d\tau = \Lambda\left(\frac{\log(x)-\mu}{\sigma}\right),$$

with distribution parameters $\varphi = (\mu, \sigma)$.

- **Johnson-SU distribution:** The pdf and cdf are

$$\phi(x|\varphi) = \frac{\sigma}{\lambda\sqrt{2\pi}\sqrt{z^2+1}} \exp\left(-\frac{1}{2}\left(\gamma + \sigma \sinh^{-1} z\right)^2\right) \quad \text{with} \quad z = \frac{x-\xi}{\lambda} \text{and} \quad y = \gamma + \sigma \sinh^{-1} z$$

and

$$\Phi(x|\varphi) = \int_0^x \phi(\tau|\varphi) d\tau = \Lambda(y),$$

with distribution parameters $\varphi = (\gamma, \sigma, \lambda, \xi)$.

- **Gamma distribution:** The pdf and cdf are

$$\phi(x|\varphi) = \frac{\beta^\alpha}{\Gamma(\alpha)} x^{\alpha-1} \exp(-\beta x),$$
$$\Phi(x|\varphi) = \frac{\gamma(\alpha, \beta x)}{\Gamma(\alpha)} = \frac{1}{\Gamma(\alpha)} \int_0^{\beta x} \tau^{\alpha-1} \exp(-\tau) d\tau,$$

with distribution parameters $\varphi = (\alpha, \beta)$. $\Gamma(\alpha)$ denotes the gamma function evaluated at α and $\gamma(\alpha, \beta x)$ the lower incomplete gamma function.

Mechanistic models. Appropriate functions $w_s(u)$ and $\varphi_s(u)$ can often be derived from the properties of the biological system, e.g., the underlying signalling pathway. This has been demonstrated for uncensored data (Hasenauer et al., 2014) and can be generalised to the case of censored data. In this case, the mixture parameters $w_s(u)$ and $\varphi_s(u)$ relate to solutions of the model describing the signalling pathway. Possible models include reaction rate equations (Hasenauer et al., 2014), the linear noise approximations (Elf & Ehrenbarg, 2003, van Kampen, 2007), effective mesoscopic rate equations (Grima, 2010, Ramaswamy et al., 2012) or moment equations (Engblom, 2006, Lee et al., 2009). These ODE models allow for a mechanistic description of the single-cell and population dynamics, in particular the explicit consideration of intrinsic and/or extrinsic noise (Swain et al., 2002). MEMO can use functions $w_s(u)$ and $\varphi_s(u)$ derived from such mechanistic models for hypothesis testing using censored and uncensored data. An example for the use of mechanistic models is provided in Section 4.3 in the main manuscript and Section S.5 in the Supplementary Material.

Semi-mechanistic models. For many processes no mechanistic models are available. In this case, flexible parametric expressions can be used to model $w_s(u)$ and $\varphi_s(u)$. These expressions depend on the mixture component and the expected condition dependence. In case of a normal distribution with mixture parameters $\varphi(u) = (\mu(u), \sigma(u))$, exemplary hypotheses regarding the condition-dependence of the mean are for example:

- The mean is constant for all u: $\mu(u, \theta) = b$ with $\theta = b$.
- The mean changes linearly with u: $\mu(u, \theta) = m \cdot u + b$ with $\theta = (m, b)^T$.
- The mean increases with u in a Hill-type manner: $\mu(u, \theta) = \frac{\mu_{\max} u^n}{K^n + u^n}$ with $\theta = (\mu_{\max}, K, n)^T$.
- The mean decreases with u in a Hill-type manner: $\mu(u, \theta) = \frac{\mu_{\max} K^n}{K^n + u^n}$ with $\theta = (\mu_{\max}, K, n)^T$.

Similar or more general dependencies might be assumed for any function $w_s(u)$ and $\varphi_s(u)$. In the presence of multiple inputs also products of univariate functions and/or multi-variate functions might be used. An example for the use of semi-mechanistic models is provided below in Section S.4.4 in the Supplementary Material.

We note that in addition to the subpopulation structure and properties, also the right censoring distributions can be parametrized. MEMO even allows for condition-dependent censoring.

D.2.3 Step 2: Parameterization of multi-experiment mixture models

The formulated multi-experiment mixture model in general posseses several unknown parameters. These unknown parameters are collected in the parameter vector $\theta \in \mathbb{R}^{n_\theta}$. MEMO implements state-of-the-art Frequentist and Bayesian methods to estimate the unknown parameters and to evaluate their uncertainties. The concepts and methods are introduced in the following.

D.2.3.1 Frequentist parameter estimation

Frequentist parameter estimation assesses the quality of the model using the likelihood of the data given the model parameters, $\mathbb{P}(\mathcal{D}|\theta)$. Assuming that measurements are i.i.d., the likelihood function for multiple experimental datasets is given as the product of the likelihood functions for the individual datasets,

$$\mathbb{P}(\mathcal{D}|\theta) = \prod_{i=1}^{I} \mathbb{P}(\mathcal{D}_i|\theta).$$

This likelihood function encodes the information about the optimal parameter values and parameter uncertainties present in the experimental data.

Maximum likelihood (ML) estimate. A maximum likelihood estimate (MLE) θ^{ML} is a parameter vector for which the likelihood takes its maximal value, hence $\forall \theta \in \Omega : \mathbb{P}(\mathcal{D}|\theta) \leq \mathbb{P}(\mathcal{D}|\theta^{\mathrm{ML}})$. Accordingly, θ^{ML} is a solution to the optimization problem

$$\begin{aligned} \theta^{\mathrm{ML}} &= \arg\max_{\theta \in \Omega} \mathbb{P}(\mathcal{D}|\theta) \\ \text{s.t.} \quad & \sum_{s=1}^{S} w_s(u_i,\theta) = 1, \quad i = 1,\ldots,I \\ & w_s(u_i,\theta) \geq 0, \quad i = 1,\ldots,I, \quad s = 1,\ldots,S. \end{aligned} \tag{2}$$

The numerics of the optimization problem (2) are improved by using the negative logarithm of the objective function,

$$J(\theta) = -\log \mathbb{P}(\mathcal{D}|\theta) = -\sum_{i=1}^{I} \log \mathbb{P}(\mathcal{D}_i|\theta).$$

This transformation conserves the optimum and the shape of the level sets. The reformulation yields the minimization problem

$$\begin{aligned} \theta^{\mathrm{ML}} &= \arg\min_{\theta \in \Omega} J(\theta) \\ \text{s.t.} \quad & \sum_{s=1}^{S} w_s(u_i,\theta) = 1, \quad i = 1,\ldots,I \\ & w_s(u_i,\theta) \geq 0, \quad i = 1,\ldots,I, \quad s = 1,\ldots,S. \end{aligned} \tag{3}$$

For more information concerning the optimization problem and the implementation in MEMO please visit MEMO's github page (https://github.com/MEMO-toolbox/MEMO).

Confidence intervals. As the measurement data are limited, the parameters can often not be determined uniquely and the corresponding estimation problem is ill-posed (Hadamard, 1902). Confidence intervals can be computed via local sensitivity-based methods, e.g., the Wald approximation (Meeker & Escobar, 1995) or the Fisher information matrix (FIM) (Murphy & van der Vaart, 2000). Alternatively, bootstrapping (Joshi et al., 2006), profile likelihoods (Murphy & van der Vaart, 2000, Raue et al., 2009) and Markov chain Monte-Carlo methods (Girolami & Calderhead, 2011, Hug et al., 2013, Wilkinson, 2007) can be used. Nowadays, Bayesian methods and profile likelihoods (Murphy & van der Vaart, 2000, Raue et al., 2013) become increasingly common as they yield very reliable results (Raue et al., 2013).

MEMO implements profile likelihood methods for a Frequentist uncertainty analysis. Profile likelihoods allow for a global uncertainty analysis of individual parameters by means of repeated optimization. Therefore, for each estimated parameter θ_i a profile likelihood is computed by fixing the parameter θ_i to a set of

values and optimizing over all remaining parameters,

$$\begin{aligned}
\mathrm{PL}(\theta_i) = &\max_{\theta_{j\neq i}} \mathbb{P}(\mathcal{D}|\theta) \\
\text{s.t.} \quad &\sum_{s=1}^{S} w_s(u_i,\theta) = 1, \quad i = 1,\dots,I \\
&w_s(u_i,\theta) \geq 0, \quad i = 1,\dots,I, \quad s = 1,\dots,S \\
&\theta \in \Omega.
\end{aligned}$$

The profile likelihood $\mathrm{PL}(\theta_i)$ is the maximal likelihood value for a given value θ_i. A particular value of θ_i can be rejected, if the profile likelihood $\mathrm{PL}(\theta_i)$ is low compared the likelihood $\mathbb{P}(\mathcal{D}|\theta^{\mathrm{ML}})$ at the globally optimal parameter point θ^{ML}. Cut-off values for the likelihood ratio for a particular significance level can be derived from the χ^2 distribution (Meeker & Escobar, 1995). For more information on the method and the implementation in MEMO please visit MEMO's github page.

D.2.3.2 Bayesian parameter estimation

Bayesian parameter estimation employes Bayes' theorem,

$$\pi(\theta|\mathcal{D}) = \frac{\mathbb{P}(\mathcal{D}|\theta)\pi(\theta)}{\mathbb{P}(\mathcal{D})} \propto \mathbb{P}(\mathcal{D}|\theta)\pi(\theta) \text{ with } \mathbb{P}(\mathcal{D}) = \int_\Omega \mathbb{P}(\mathcal{D}|\theta)\pi(\theta)d\theta. \tag{4}$$

The posterior distribution $\pi(\theta|\mathcal{D})$ of the parameters θ given the data $\mathcal{D}$ is determined by the likelihood $\mathbb{P}(\mathcal{D}|\theta)$, the prior distribution $\pi(\theta)$ and the marginal probability $\mathbb{P}(\mathcal{D})$. The marginal probability is independent of the parameters. Therefore, the posterior probability is proportional to the product of likelihood and prior.

MEMO implements optimization- and sampling-based approaches for the analysis of the posterior distribution.

Maximum a posteriori (MAP) estimate. A maximum a posteriori estimate θ^{MAP} is a parameter vector for which the posterior probability takes its maximal value, hence $\forall\theta \in \Omega : \pi(\theta|\mathcal{D}) \leq \pi(\theta^{\mathrm{MAP}}|\mathcal{D})$. Thus, MAP estimates provide the best agreement of model and data taking the prior knowledge into account. As the posterior probability is proportional to the product of likelihood and prior probability, θ^{MAP} is a solution to the optimization problem

$$\begin{aligned}
\theta^{\mathrm{MAP}} = &\arg\max_{\theta\in\Omega} \mathbb{P}(\mathcal{D}|\theta)\pi(\theta) \\
\text{s.t.} \quad &\sum_{s=1}^{S} w_s(u_i,\theta) = 1, \quad i = 1,\dots,I \\
&w_s(u_i,\theta) \geq 0, \quad i = 1,\dots,I, \quad s = 1,\dots,S.
\end{aligned}$$

This optimization problem can be reformulated similar to the corresponding ML problem (2). In MEMO, multi-start local optimization is used to solve the reformulated optimization problems. The same optimization settings are used as for the maximum likelihood problem (see Section D.2.3.1), and similar visualizations are available.

Credibility intervals. In Bayesian statistics, the a posteriori uncertainty of model parameters depends on the information content of the data – encoded in the likelihood – and the prior information. Bayesian credibility intervals for the parameters (Chen & Shao, 1999) can, for instance, be computed using Laplace approximations at the MAP estimate, profile posteriors (Hug et al., 2013) and Markov chain Monte-Carlo (MCMC) methods (Girolami & Calderhead, 2011, Hug et al., 2013, Wilkinson, 2007). The profile posterior calculation is similar to the profile likelihood calculation. The same ideas and methods are exploited but the function $\mathbb{P}(\mathcal{D}|\theta)\pi(\theta)$ is optimized repeatedly to determine the uncertainty of each individual parameter.

MCMC methods generate a chain of parameters, $\theta^1, \theta^2, \dots, \theta^{ns}$, by exploring the posterior distribution $\pi(\theta|\mathcal{D})$. After convergence of the chain, the set $\mathcal{S} = \{\theta^j\}_{j=1}^{ns}$ provides a representative sample from the posterior distribution. This sample $\mathcal{S}$ reveals parameter uncertainties as well as correlations of parameters. The most common choice for the $100(1-\alpha)\%$ credibility interval for a parameter θ_j is the $100(1-\alpha)$-th percentiles of the sample $\mathcal{S}$ (DiCiccio & Efron, 1996).

D.2.4 Step 3: Testing of hypotheses via model selection

To compare competing model hypotheses, MEMO allows for the comparison of user-defined models as well as for an automated analysis of the population structure.

D.2.4.1 Model selection methods

For model selection, three different types of criteria are implemented in MEMO: Likelihood ratio test; Akaike information criterion (AIC); and Bayesian information criterion (BIC). The likelihood ratio test requires models to be nested, which is not required by AIC and BIC. In the following, the different criteria are introduced, and the index k is used to denote a particular model:

Likelihood ratio test. The likelihood ratio test is a statistical test used to compare a null model $\mathcal{M}_k$ and an alternative model $\mathcal{M}_{k'}$ (Wilks, 1938). The null model has to represent a special case of the alternative model, thus the models have to be nested. The likelihood ratio $\mathbb{P}(\mathcal{D}|\theta_{k'}^{\mathrm{ML}}))/\mathbb{P}(\mathcal{D}|\theta_k^{\mathrm{ML}}))$ indicates how much more likely the data are under the alternative model compared to the null model. The logarithm of this test statistic

$$\Xi = -\log(\mathbb{P}(\mathcal{D}|\theta_k^{\mathrm{ML}})) + \log(\mathbb{P}(\mathcal{D}|\theta_{k'}^{\mathrm{ML}}))$$

is approximately χ^2 distributed with degrees of freedom equal to $n_{\theta,k'} - n_{\theta,k}$. Using the χ^2 distribution, the significance level for a rejection of the null model can be computed. In this manuscript we considered a p-value of 0.05 as substantial.

Akaike information criterion. The AIC is an information theoretical measure of the quality of a statistical model (Akaike, 1973). It accounts for fit and complexity of a model $\mathcal{M}_k$,

$$\mathrm{AIC}_k = -2\log(\mathbb{P}(\mathcal{D}|\theta_k^{\mathrm{ML}})) + 2n_{\theta,k}.$$

The number of parameters of model $\mathcal{M}_k$ is denoted by $n_{\theta,k}$. Models with lower AICs are preferable and the best model $\mathcal{M}_{k^*}$ (according to AIC) possesses the minimal AIC, $\mathrm{AIC}_{\min} = \mathrm{AIC}_{k^*}$ with $k^* = \arg\min_k \mathrm{AIC}_k$. In practice, given the best model $\mathcal{M}_{k^*}$, the level of empirical support for model $\mathcal{M}_k$ is considered to be

- *substantial* for $0 < \mathrm{AIC}_k - \mathrm{AIC}_{\min} < 2$,
- *considerably less* for $4 < \mathrm{AIC}_k - \mathrm{AIC}_{\min} < 7$, and
- *essentially none* for $10 < \mathrm{AIC}_k - \mathrm{AIC}_{\min}$.

For details we refer to (Burnham & Anderson, 2002). In this manuscript we considered a difference in the AIC values of 4 to be substantial.

The application of AIC is well justified if the sample-size $n_{\mathcal{D}}$ is large. In the case of small sample-sizes, the corrected AIC provides a bias adjustment (Hurvich & Tsia, 1989),

$$\mathrm{AIC}_{c,k} = -2\log(\mathbb{P}(\mathcal{D}|\theta_k^{\mathrm{ML}})) + \frac{2n_{\theta,k}n_{\mathcal{D}}}{n_{\mathcal{D}} - n_{\theta,k} - 1}.$$

In accordance with this formula, AIC and corrected AIC should only be applied for $n_{\mathcal{D}} > n_{\theta,k} + 1$.

Bayesian information criterion. The BIC is a probabilistic model selection criterion, derived using Bayesian arguments. It accounts for fit and model complexity,

$$\mathrm{BIC}_k = -2\log(\mathbb{P}(\mathcal{D}|\theta_k^{\mathrm{ML}})) + n_{\theta,k}\log n_{\mathcal{D}}.$$

The weight of the model complexity depends on the sample-size $n_{\mathcal{D}}$. Similar to the AIC, models with lower BICs are preferable. The best model $\mathcal{M}_{k^*}$ (according to BIC) possesses the minimal BIC, $\mathrm{BIC}_{\min} = \mathrm{BIC}_{k^*}$ with $k^* = \arg\min_k \mathrm{BIC}_k$. In practice, given the best model $\mathcal{M}_{k^*}$, the evidence against model $\mathcal{M}_k$ is considered to be

- *not worth more than a bare mention* for $0 < \mathrm{BIC}_k - \mathrm{BIC}_{\min} < 2$,

- *positive* for $2 < \text{BIC}_k - \text{BIC}_{\text{min}} < 6$,
- *strong* for $6 < \text{BIC}_k - \text{BIC}_{\text{min}} < 10$, and
- *very strong* for $10 < \text{BIC}_k - \text{BIC}_{\text{min}}$.

For details we refer to (Kass & Raftery, 1995). In this manuscript we considered a difference in the BIC values of 6 to be substantial.

The three model selection criteria implemented in MEMO enable the selection of appropriate models based on optimization results. Parameter uncertainties as well as prior knowledge is not taken into account. To consider this, Bayes factors can be used for model selection (Kass & Raftery, 1995). As the robust and efficient computation of Bayes factors can be challenging and might require problem-specific implementations, MEMO does not provide this functionality. Any methods for Bayes factor calculation, including bridge sampling (Meng & Wong, 1996), importance sampling (Neal, 2001), nested sampling (Skilling, 2006), Laplace Metropolis (Lewis & Raftery, 1997), density estimation-based approaches (Kramer et al., 2010) and thermodynamic integration (Vyshemirsky & Girolami, 2008), can however be easily coupled with MEMO. For this purpose, MEMO provides an easy-to-use objective function interface.

D.2.4.2 Automated unraveling of subpopulation structure

The comparison of many alternative hypotheses can become cumbersome as it requires the definition of many models. Therefore, in addition to the comparison of user-defined models, MEMO provides backward model selection capabilities. This backward model selection supports the automated unraveling of the subpopulation structure. For single- and multi-experiment single-cell data, the implemented backward model selection iteratively simplifies the model by eliminating subpopulation which are not supported by the experimental data. Therefor all possible models which possess in the experimental conditions one subpopulation less than the current model are generated, compiled and fitted. Among the fitted model the best one is selected using the specified selection criteria (likelihood ratio, AIC or BIC) along with a cut-off threshold. This process is repeated until no further improvement of the model is achieved.

The backward model selection method implemented in MEMO is computationally relatively efficient. In comparison to a brute-force approach not all model alternatives are analyzed, which decreases the computational complexity significantly. Furthermore, the automatic generation reduces the work load for the user and enables the analysis of thousands of model alternatives.

D.2.5 Step 4: Interpretation and further analysis

Parameter estimation and model selection provide a set of mixture models capturing the data. MEMO provides different routines to visualize these models along with the measurement data and to compare them. Beyond the sole analysis of available data, multi-experiment mixture models enable predictions. For example, mixture models with relative subpopulation sizes $w_s(u)$ and subpopulation mixture parameters $\varphi_s(u)$ that functionally depend on the experimental condition can be used to predict the outcomes of future experiments with different experimental conditions. Therefore, the mixture properties just have to be evaluated for the respective experimental setting u. MEMO also allows for the assessment of the prediction uncertainty, e.g. by using the parameter samples obtained via MCMC sampling (Section D.2.3.2). This enables a thorough analysis of the predictive power of models and model validation.

The mixture model and its parameters can furthermore be used for subsequent analysis of the data generating process. The calculated subpopulation sizes $w_s(u)$ and subpopulation mixture parameters $\varphi_s(u)$ can, for instance, be used to unravel key properties of the underlying signaling pathways such as cooperative interactions between proteins. The processed data can even be used to inform mechanistic models. This was demonstrated in several recent publications (Duffy et al., 2012, Heinrich et al., 2013, Song et al., 2010). For the use in subsequent analysis, MEMO provides estimates and confidence or credibility intervals for all essential model quantities. Also model averaging (Ando, 2008, Burnham & Anderson, 2004) can easily be realized to account for multiple model alternatives.

D.2.6 Gradients of objective function

To facilitate the convergence and reduce the computational cost of the optimization, MEMO provides the optimization routine with the gradient of the objective function with respect to the parameter vector θ. In the following subsections we provide all gradients needed for the case of uncensored data (x_i^j), interval censored data ($\underline{\overline{x}}_i^j$) and right censored data ($\overline{x}_i^j$), assuming a model for the censoring. For maximum generality the distribution parameters φ are assumed to be functions of θ.

D.2.6.1 Gradient of the log-likelihood function

Since we optimize the logarithm of the objective function, the gradient of this log-likelihood function is provided.

For the case of uncensored data the gradient reads

$$\frac{d\log(\mathbb{P}(\mathcal{D}_i|\theta))}{d\theta} \propto \frac{d}{d\theta}\log\left(\prod_{j=1}^{n_{x,i}} p(x_i^j|\theta,u_i)\right) = \sum_{j=1}^{n_{x,i}} \frac{d}{d\theta}\log\left(p(x_i^j|\theta,u_i)\right) = \sum_{j=1}^{n_{x,i}} \frac{1}{A}\frac{dp(x_i^j|\theta,u_i)}{d\theta},$$

with $A = p(x_i^j|\theta,u_i)$.

For the case of interval censored data the gradient reads

$$\begin{aligned}\frac{d\log(\mathbb{P}(\mathcal{D}_i|\theta))}{d\theta} &\propto \frac{d}{d\theta}\log\prod_{l=1}^{n_{\underline{\overline{x}},i}} \underline{\overline{p}}(\underline{\overline{x}}_i^l|\theta,u_i)\\ &= \sum_{l=1}^{n_{\underline{\overline{x}},i}} \frac{d}{d\theta}\log\left(\underline{\overline{p}}(\underline{\overline{x}}_i^l|\theta,u_i)\right)\\ &= \sum_{l=1}^{n_{\underline{\overline{x}},i}} \frac{1}{B}\frac{d}{d\theta}\left(P(\underline{\overline{x}}_i^l|\theta,u_i) - P(\underline{\overline{x}}_i^l - \Delta x|\theta,u_i)\right)\end{aligned}$$

with $B = P(\underline{\overline{x}}_i^l|\theta,u_i) - P(\underline{\overline{x}}_i^l - \Delta x|\theta,u_i)$.

For the case of right censored data the gradient reads

$$\begin{aligned}\frac{d\log(\mathbb{P}(\mathcal{D}_i|\theta))}{d\theta} &\propto \frac{d}{d\theta}\log\left(\prod_{j=1}^{n_{x,i}} p(x_i^j|\theta,u_i)(1-P_c(x_i^j|\theta,u_i))\right)\left(\prod_{k=1}^{n_{\overline{x},i}} p_c(\overline{x}_i^k|\theta,u_i)(1-P(\overline{x}_i^k|\theta,u_i))\right)\\ &= \sum_{j=1}^{n_{x,i}} \frac{d}{d\theta}\log p(x_i^j|\theta,u_i)(1-P_c(x_i^j|\theta,u_i)) + \sum_{k=1}^{n_{\overline{x},i}} \frac{d}{d\theta}\log p_c(\overline{x}_i^k|\theta,u_i)(1-P(\overline{x}_i^k|\theta,u_i))\\ &= \sum_{j=1}^{n_{x,i}} \frac{1}{C}\frac{d}{d\theta}(p(x_i^j|\theta,u_i)(1-P_c(x_i^j|\theta,u_i))) + \sum_{k=1}^{n_{\overline{x},i}} \frac{1}{D}\frac{d}{d\theta}(p_c(\overline{x}_i^k|\theta,u_i)(1-P(\overline{x}_i^k|\theta,u_i)))\\ &= \sum_{j=1}^{n_{x,i}} \frac{1}{C}\left(\frac{dp(x_i^j|\theta,u_i)}{d\theta}(1-P_c(x_i^j|\theta,u_i)) - p(x_i^j|\theta,u_i)\frac{dP_c(x_i^j|\theta,u_i)}{d\theta}\right)\\ &+ \sum_{k=1}^{n_{\overline{x},i}} \frac{1}{D}\left(\frac{dp_c(\overline{x}_i^k|\theta,u_i)}{d\theta}(1-P(\overline{x}_i^k|\theta,u_i)) - p_c(\overline{x}_i^k|\theta,u_i)\frac{dP(\overline{x}_i^k|\theta,u_i)}{d\theta}\right)\end{aligned}$$

with $C = p(x_i^j|\theta,u_i)(1-P_c(x_i^j|\theta,u_i))$ and $D = p_c(\overline{x}_i^k|\theta,u_i)(1-P(\overline{x}_i^k|\theta,u_i))$.

For the case of left censored data the gradient reads

$$\begin{aligned}
\frac{d\log(\mathbb{P}(\mathcal{D}_i|\theta))}{d\theta} &\propto \frac{d}{d\theta}\log\left(\prod_{j=1}^{n_{x,i}} p(x_i^j|\theta,u_i)(P_l(x_i^j|\theta,u_i))\right)\left(\prod_{m=1}^{n_{\underline{x},i}} p_l(\underline{x}_i^m|\theta,u_i)(P(\underline{x}_i^m|\theta,u_i))\right)\\
&= \sum_{j=1}^{n_{x,i}} \frac{d}{d\theta}\log p(x_i^j|\theta,u_i)(P_l(x_i^j|\theta,u_i)) + \sum_{m=1}^{n_{\underline{x},i}} \frac{d}{d\theta}\log p_l(\underline{x}_i^m|\theta,u_i)(P(\underline{x}_i^m|\theta,u_i))\\
&= \sum_{j=1}^{n_{x,i}} \frac{1}{E}\frac{d}{d\theta}\left(p(x_i^j|\theta,u_i)(P_l(x_i^j|\theta,u_i))\right) + \sum_{m=1}^{n_{\underline{x},i}} \frac{1}{F}\frac{d}{d\theta}\left(p_l(\underline{x}_i^m|\theta,u_i)(P(\underline{x}_i^m|\theta,u_i))\right)\\
&\sum_{j=1}^{n_{x,i}} \frac{1}{E}\left(\frac{dp(x_i^j|\theta,u_i)}{d\theta}P_l(x_i^j|\theta,u_i) + p(x_i^j|\theta,u_i)\frac{dP_l(x_i^j|\theta,u_i)}{d\theta}\right)\\
&+ \sum_{m=1}^{n_{\underline{x},i}} \frac{1}{F}\left(\frac{dp_l(\underline{x}_i^m|\theta,u_i)}{d\theta}P(\underline{x}_i^m|\theta,u_i) + p_l(\underline{x}_i^m|\theta,u_i)\frac{dP(\underline{x}_i^m|\theta,u_i)}{d\theta}\right)
\end{aligned}$$

with $E = p(x_i^j|\theta,u_i)(P_l(x_i^j|\theta,u_i))$ and $F = p_l(\underline{x}_i^m|\theta,u_i)(P(\underline{x}_i^m|\theta,u_i))$.

For the case of interval and right censored data the gradient reads

$$\begin{aligned}
\frac{d\log(\mathbb{P}(\mathcal{D}_i|\theta))}{d\theta} &= \frac{d}{d\theta}\log\left(\prod_{l=1}^{n_{\overline{\underline{x}},i}} p(\overline{\underline{x}}_i^l, \overline{\underline{x}}_i^l \leq X_{i,c},\theta,u_i)\prod_{k=1}^{n_{\overline{x},i}} p(\overline{x}_i^k, \overline{x}_i^k < X_i^+,\theta,u_i)\right)\\
&= \sum_{l=1}^{n_{\overline{\underline{x}},i}} \frac{d}{d\theta}\left(\log\left(\left(P(\overline{\underline{x}}_i^l|\theta,u_i) - P(\overline{\underline{x}}_i^l - \Delta x|\theta,u_i)\right)\left(1 - P_c(\overline{\underline{x}}_i^l|\theta,u_i)\right)\right)\right)\\
&+ \sum_{k=1}^{n_{\overline{x},i}} \frac{d}{d\theta}\left(\log\left(\left(P_c(\overline{x}_i^k|\theta,u_i) - P_c(\overline{x}_i^k - \Delta x|\theta,u_i)\right)\left((1 - P\overline{x}_i^k|\theta,u_i)\right)\right)\right)\\
&= \sum_{l=1}^{n_{\overline{\underline{x}},i}} \frac{1}{G}\left(\int_{\overline{\underline{x}}_i^l-\Delta x}^{\overline{\underline{x}}_i^l} \frac{dp(x|\theta,u_i)}{d\theta}(1 - P_c(x|\theta,u_i)) - p(x|\theta,u_i)\frac{dP_c(x|\theta,u_i)}{d\theta}dx\right)\\
&+ \sum_{k=1}^{n_{\overline{x},i}} \frac{1}{H}\left(\int_{\overline{x}_i^k-\Delta x}^{\overline{x}_i^k} \frac{dp_c(x|\theta,u_i)}{d\theta}(1 - P(x|\theta,u_i)) - p_c(x|\theta,u_i)\frac{dP(x|\theta,u_i)}{d\theta}dx\right)
\end{aligned}$$

with $G = \int_{\overline{\underline{x}}_i^l-\Delta x}^{\overline{\underline{x}}_i^l} p(x|\theta,u_i)(1 - P_c(x|\theta,u_i))dx$ and $H = \int_{\overline{x}_i^k-\Delta x}^{\overline{x}_i^k} p_c(x|\theta,u_i)(1 - P(x|\theta,u_i))dx$.

The case of left and interval censored data can be derived accordingly.

The gradients of the mixture distributions and probability densities that are part of this gradient can be found in the subsequent sections.

D.2.6.2 Gradient of the mixture distribution

The gradient of the mixture distribution

$$p(x|\theta) = \sum_{s=1}^{S} w_s(\theta)\phi(x|\theta),$$

with respect to the parameter vector θ is

$$\frac{dp(x|\theta)}{d\theta} = \sum_{s=1}^{S} \frac{dw_s(\theta)}{d\theta}\phi(x|\theta) + w_s(\theta)\frac{d\phi(x|\theta)}{d\theta}.$$

The gradient of the cumulative mixture distribution

$$P(x|\theta) = \sum_{s=1}^{S} w_s(\theta)\Phi(x|\theta),$$

with respect to the parameter vector θ is

$$\frac{dP(x|\theta)}{d\theta} = \sum_{s=1}^{S} \frac{dw_s(\theta)}{d\theta} \Phi\left(x|\theta\right) + w_s(\theta) \frac{d\Phi(x|\theta)}{d\theta}.$$

D.2.6.3 Gradient of the probability densities

In the following the gradients of the probability density functions ϕ (see D.2.2) and cumulative probability densities (cdf) Φ with respect to the parameter vector θ are provided for different distribution types.

- **Normal distribution:**
  With
  $$y = \frac{x-\mu}{\sigma}$$
  the gradients of the pdf and cdf are
  $$\begin{aligned}\frac{d\phi(x|\theta)}{d\theta} &= \frac{1}{\sqrt{2\pi\sigma^2}} \exp\left(-\frac{1}{2}y^2\right)\left(y\frac{\partial\mu}{\partial\theta} + (y^2-1)\frac{\partial\sigma}{\partial\theta}\right),\\ \frac{d\Phi(x|\theta)}{d\theta}(x) &= -\frac{1}{\sqrt{2\pi}\sigma} \exp\left(-\frac{1}{2}y^2\right)\left(\frac{d\mu}{d\theta} + y\frac{d\sigma}{d\theta}\right).\end{aligned}$$

- **Log-normal distribution:**
  With
  $$y = \frac{\log(x)-\mu}{\sigma}$$
  the gradients of the pdf and cdf are
  $$\begin{aligned}\frac{d\phi(x|\theta)}{d\theta} &= \frac{1}{\sqrt{2\pi\sigma^2}x} \exp\left(-\frac{1}{2}y^2\right)\left(y\frac{\partial\mu}{\partial\theta} + (y^2-1)\frac{\partial\sigma}{\partial\theta}\right),\\ \frac{d\Phi(x|\theta)}{d\theta} &= -\frac{1}{\sqrt{2\pi}\sigma} \exp\left(-\frac{1}{2}y^2\right)\left(\frac{d\mu}{d\theta} + y\frac{d\sigma}{d\theta}\right).\end{aligned}$$

- **Johnson-SU distribution:**
  With
  $$z = \frac{x-\xi}{\lambda} \quad \text{and} \quad y = \gamma + \sigma \mathrm{sinh}^{-1} z$$
  the gradients of the pdf and cdf are
  $$\begin{aligned}\frac{d\phi(x|\theta)}{d\theta} &= \frac{\sigma z^2 + \sigma^2 zy\sqrt{z^2+1} - \sigma(z^2+1)}{\lambda^2(z^2+1)^{\frac{3}{2}}}\frac{\partial\lambda}{\partial\theta} + \frac{1-\sigma y \mathrm{sinh}^{-1} z}{\lambda\sqrt{z^2+1}}\frac{\partial\sigma}{\partial\theta}\\ &+ \frac{\sigma z + \sigma^2 y\sqrt{z^2+1}}{\lambda^2(z^2+1)^{\frac{3}{2}}}\frac{\partial\xi}{\partial\theta} - \frac{\sigma y}{\lambda\sqrt{z^2+1}}\frac{\partial\gamma}{\partial\theta},\\ \frac{d\Phi(x|\theta)}{d\theta} &= \frac{1}{\sqrt{2\pi}} \exp\left(-\frac{1}{2}y^2\right)\left(-\frac{\sigma}{\lambda\sqrt{1+z^2}}\left(z\frac{\partial\lambda}{\partial\theta} + \frac{\partial\xi}{\partial\theta}\right) + \frac{\partial\gamma}{\partial\theta} + \mathrm{sinh}^{-1} z\frac{\partial\sigma}{\partial\theta}\right).\end{aligned}$$

- **Gamma distribution:** The gradients of the pdf and cdf are
  $$\begin{aligned}\frac{d\phi(x|\theta)}{d\theta} &= \phi(x|\theta)\left(\log(\beta) - \underbrace{\frac{\Gamma'(\alpha)}{\Gamma(\alpha)}}_{=\Psi(\alpha)} + \log(x)\right)\frac{\partial\alpha}{\partial\theta} + \phi(x|\theta)(\frac{\alpha}{\beta} - x)\frac{\partial\beta}{\partial\theta},\\ \frac{d\Phi(x|\theta)}{d\theta} &= \left(\frac{\frac{\partial\gamma(\alpha,\beta x)}{\partial\alpha}}{\Gamma(\alpha)} - \Phi(x|\varphi)\Psi(\alpha)\right)\frac{\partial\alpha}{\partial\theta} + \frac{(\beta x)^{\alpha-1}\exp(-\beta x)x}{\Gamma(\alpha)}\frac{\partial\beta}{\partial\theta},\end{aligned}$$

  in which $\Gamma(\alpha)$ denotes the gamma function evaluated at α, $\Gamma'(\alpha) = \frac{d\Gamma(\alpha)}{d\alpha} = \int_0^\infty x^{\alpha-1}\log(x)\exp(-x)dx$ and $\Psi(\alpha)$ denotes the digamma function.

D.3 Implementation of MEMO

MEMO is implemented in Matlab and has been tested under Version R2014a under Mac OS, Linux and Windows 7.

D.3.1 Requirements

In addition to the provided Matlab functions, the Parameter EStimation Toolbox (PESTO) developed by Jan Hasenauer (Institute of Computational Biology, Helmholtz Zentrum München, Germany) is required. PESTO is included in the MEMO download. Furthermore the Symbolic Math Toolbox and the Statistics and Machine Learning Toolbox of Matlab are required. For MCMC sampling the MATLAB toolbox DRAM (Haario et al., 2006) is required.

D.3.2 Model formulation and data format

MEMO uses a single definition file, an m-file, for each finite mixture model. This definition file is provided by the user and contains the specification of the model $\mathcal{M}$ and the data $\mathcal{D}$. An example is shown in Figure D1. The model setup starts by defining the struct `parameters` that specifies the parameters and their range for optimization, sampling and profile likelihood estimation. Since MEMO uses symbolic computation, the parameters have to be defined symbolically and then stacked in a parameter vector. Furthermore, for each parameter a name has to be specified that is used for plots and other visualizing routines and should therefore comply to a LaTeX interpretable format (Figure D1, lines 3 - 21).
In addition, the definition file provides the structure of the finite mixture model for the quantity of interest. The type of distribution can be chosen and the unit of the modeled data can be specified. The latter will be used as label in visualization routines. Furthermore, `M` contains the specification of the different experimental conditions that are subject to analysis. For every experimental condition the subpopulation structure, namely the number of subpopulations and their distribution type, can be specified. The symbolic parameters are assigned to their respective experimental condition and subpopulations. If modeling of the distribution of the censoring quantity is chosen, the same is performed for the censoring quantity in a second struct `Mc` (Figure D1, lines 23 - 52).
The definition file also links the data to the model. All information on the data is stored in a cell array `D`, whose length is the number of different experimental conditions. For every dataset a title specifying the experimental condition and the vectors containing the data, grouped by the type of data (uncensored / interval censored (`Tm`), right / left censored (`Tc`)), has to be provided. Furthermore, the type of censoring in `Tc` has to be specified. Possible options are 'left' or 'right'. Data for the different experimental conditions can be loaded from separate data files (Figure D1, lines 34, 54-60) or the data and the associated information can be specified in the definition file itself. An exemplary data file is shown in Figure D2. Subsequent to the definition, model and data structure are compiled by the MEMO function `getMixtureModel.m` that creates functional expressions of parameters and derivatives (Figure D1, lines 70 + 71).

D.3.3 Likelihood calculation

To calculate the likelihood for a certain model given the data, MEMO provides the functions `logLikelihoodMM.m` and `logLikelihoodMMc.m` for model setups without and with model of the censoring distribution, respectively. These functions take the parameters struct, the model struct(s) and the data struct as an input. If two outputs are specified also the gradient of the likelihood is evaluated. Examples for the function call are shown in Figure D3.

D.3.4 Optimization

To find the optimal parameters of the finite mixture model, given the data, the function `optimizeMultiStart.m` is called (Figure D4). This function performs a multi-start local optimization. The first input argument is the `parameters` struct. The second argument is a function handle to the respective likelihood function (`logLikelihoodMM.m` or `logLikelihoodMMc.m`) and the last argument is a struct with options, e.g. for `fmincon` (see m-file of `optimizeMultiStart.m` for further information). The default options are shown

```
%% model illustration MEMO model setup for a model with log-normally distributed events ...
    of interest and Johnson SU distributed censoring events.
%%
%% SPECIFY MODEL PARAMETERS
syms    mu_exp1_sub1 mu_exp1_sub2 log_sigma_exp1_sub1 log_sigma_exp1_sub2 w_exp1_sub1  ...
        gamma_cen  esigma_cen  elambda_cen  exi_cen;

parameters.sym  = ...
                    [mu_exp1_sub1; mu_exp1_sub2; log_sigma_exp1_sub1; ...
                        log_sigma_exp1_sub2; w_exp1_sub1;  ...
                                        gamma_cen; esigma_cen; elambda_cen; exi_cen];

% parameter names for plotting and other vizualization
parameters.name = {'\mu_{exp1,sub1}'; 'log(\sigma_{exp1,sub1})'; '\mu_{exp1,sub2}'; ...
    'log(\sigma_{exp1,sub2})'; 'w_{exp1,sub1}'; ...
                                    '\gamma_{cen}'; 'log(\sigma_{cen})'; ...
                                        'log(\lambda_{cen})'; 'log(\xi_{cen})' };

parameters.number = length(parameters.sym);

parameters.min  = [ log(5); log(1e-1); 0; ...
                                        -20   ; log(1e-4); log(5); log(5) ];

parameters.max  = [  log(2e3); log(1e1); 1; ...
                                        20; log(1e4); log(2e3); log(2e3)];

%% SPECIFY MODEL AND DATA
M.mixture.type = 'log-normal'; % specification of distribution type
M.label.x = 'time [min]'; % x-label for model-data-comparison plots, in units of ...
    measurement data
M.label.y = 'probability density'; % y-label for model-data-comparison plots

Mc.mixture.type = 'Johnson SU';
Mc.label.x = 'time [min]';
Mc.label.y = 'probability density';

%% Experimental condition No. 1

data_file_exp_1; % load file were data for Exp.1 is stored

i = 1 ; % set counter for experimental condition

% model for censoring times of exp.1
M.experiment(i).name  = tit; % title defined in data file data_file_exp_1.m
M.experiment(i).size  = 2;  % number of mixture components=subpopulations
M.experiment(i).w     = {1-w_exp1_sub1,w_exp1_sub1}; % weights
M.experiment(i).mu    = {mu_exp1_sub2,mu_exp1_sub1};  % one μ per subpopulations
M.experiment(i).sigma = {exp(log_sigma_exp1_sub2),exp(log_sigma_exp1_sub1)}; % one σ per ...
    subpopulation, exp(sigma) for parmeter estimation in logarithmic space

% model for censoring times of exp.1
Mc.experiment(i).name   = tit;
Mc.experiment(i).size   = 1;
Mc.experiment(i).w      = {          1 };
Mc.experiment(i).gamma  = {     gamma_cen };
Mc.experiment(i).sigma  = { exp(esigma_cen)};
Mc.experiment(i).lambda = {exp(elambda_cen)};
Mc.experiment(i).xi     = {    exp(exi_cen)};

% asign data to Data struct
D{i}.name = tit;  % title defined in data file data_file_exp_1.m
D{i}.description = []; % optional
D{i}.data.uncensored = Tm;
D{i}.data.censored = Tc;
D{i}.data.cen_type = 'right';
D{i}.observation_interval = 5; % in this example data is interval censored and inter ...
    observation time is 5 min

%% Experimental condition No. 2
i = i+1 ;

% and so on


% Compile model
% (This generates the functional expression of parameters and derivatives.)
[M,parameters.constraints] = getMixtureModel(M,parameters.sym);
Mc = getMixtureModel(Mc,parameters.sym);
```

Figure D1: Example model definition in MEMO.

```
% data measured in Mad3 knockout strain
tit=('Delta Mad3'); % title of experimental condition

% experimental condition: normal amount of Mad1 and Mad2, no expression of Mad3
Mad1 = 1;
Mad2 = 1;
Mad3 = 0;

% data of single cells for event of iterest
% here interval censored but not right censord data points
Tm=[
55
55
55
45
60
50
];

% data of censoring event. here right censored data points, in this example there is no ...
    right censored data
Tc=[];
```

Figure D2: Example MEMO data file.

```
% without model for censoring event
[logL,grad] = logLikelihoodMM(theta,M,D,options)

% with model for censoring event Mc
[logL,grad] = logLikelihoodMMc(theta,M,Mc,D,options)
```

Figure D3: Function calls to obtain the likelihood and its gradient.

in Figure D5. The optimization time scales linearly with the number of data points. The number of experimental conditions, which contain these data points, has almost no effect. However with increasing number of experimental conditions, the number of parameters potential increases, which may require a higher number of starts in the multi-start optimization and result in an increased computational complexity.

D.3.5 Automated backward model selection

The automated model backward selection is performed by calling the function `performModelSelection.m` which takes the model with the maximum number of components per experimental condition `M`, the parameters, the data struct `D` and options and returns the most likely model consisting of `M_red` and `Mc_red`, and the reduced parameter struct `parameters_red`. For details on the method please see Section D.2.4.2.

```
%% OPTIMIZATION
% Options
options_fit.n_starts = 200;
options_fit.plot = plot_opt;
options_fit.proposal = 'latin hypercube';
options_fit.fmincon = optimset('algorithm','interior-point',...
                               'display','off',...
                               'GradObj','on',...
                               'MaxIter',4000,...
                               'MaxFunEvals',4000*parameters.number);
% Run estimation
[parameters,M.fh.fit] = optimizeMultiStart(parameters,@(theta,opt)
logLikelihoodMMc(theta,M,Mc,D,opt),options_fit);
```

Figure D4: Exemplary call of function `optimizeMultiStart.m`.

```
options.fmincon = optimset('algorithm','interior-point',...
                           'display','off',...
                           'GradObj','on',...
                           'MaxIter',3000,...
                           'MaxFunEvals',3000*parameters.number);
options_MS.linprog = optimset('algorithm','active-set');
options.n_starts = 20;
options.proposal = 'latin hypercube';
options.plot = 'true';
options.mode = 'normal'; % 'silent';
options.logPost_options.sign = 'negative';
options.logPost_options.grad_ind = [1:parameters.number]';
options.fh = [];
```

Figure D5: Default values for the options of multi-start optimization.

Command Window

```
>>  printModel(M);


Type of mixture: log-normal

 ------------------------------------------------------------------------------------------------------------
| Dataset       |                          component 1                          |                      component 2                          |
|---------------|---------------------------------------------------------------|-----------------------------------------------------------|
|               |        w         |      mu      |             sigma           |      w        |      mu      |            sigma           |
|---------------|---------------------------------------------------------------|-----------------------------------------------------------|
| dataset_1     | 1 - w_exp1_sub1 | mu_exp1_sub2 | exp(log_sigma_exp1_sub2) | w_exp1_sub1 | mu_exp1_sub1 | exp(log_sigma_exp1_sub1) |
 ------------------------------------------------------------------------------------------------------------

>>
```

Figure D6: Output of function `printModel.m` to matlab command window for the example in Figure D1.

D.3.6 Goodness of fit analysis

The Goodness of fit of a model can be assessed via the function `analyzeLogPostDistribution.m`. The function estimates the expected distribution of the likelihood function values, assuming that the model `M` parametrized with `parameters` describes the data generating distribution. To estimate the distribution of the likelihood function values, artificial measurement data are generated from the mixture model, and the log-posterior function is evaluated.

D.3.7 Uncertainty analysis

MEMO provides two different approaches to assess the uncertainty of the estimated parameters. There are inbuilt functions for profile likelihood estimation and MCMC sampling. For details we refer to Section D.2.3. These function can be called by the functions `computeProfiles.m` and `computeMCMCsample_DRAM.m` included in PESTO.

D.3.8 Visualization

MEMO provides the user with a variety of visualization functions. The function `printModel.m` prints the specified model in the Matlab command window either with symbolic names of the parameters or, if the `parameters` struct with optimization results is provided as input, the parameter values of the best fit (see Figure D6 for example output). Parameter values shown by `printModel.m` are the parameters θ (not $\xi = \log(\theta)$, see Section D.2.3).
The function `plotMixtureModel.m` visualizes the model data fit graphically by creating a Matlab figure with subplots for every experimental condition. There are several possible options, including visualization of parameter uncertainties obtained by MCMC sampling.

References

Akaike, H. (1973). Information theory and an extension of the maximum likelihood principle. In 2nd International Symposium on Information Theory, Tsahkadsor, Armenian SSR. Akademiai Kiado, vol. 1, 267–281.

Ando, T. (2008). Bayesian model averaging and bayesian predictive information criterion for model selection. J. Japan Statist. Soc., 38(2), 243–257.

Burnham, K. P. & Anderson, D. R. (2002). Model selection and multimodel inference: A practical information-theoretic approach. Springer-Verlag New York, 2nd ed.

Burnham, K. P. & Anderson, D. R. (2004). Multimodel inference: Understanding AIC and BIC in model selection. Sociological Methods & Research, 33(2), 261–304.

Chen, M.-H. & Shao, Q.-M. (1999). Monte Carlo estimation of Bayesian credible and HPD intervals. J. Comput. Graphical Statist., 8(1), 69–92.

DiCiccio, T. J. & Efron, B. (1996). Bootstrap confidence intervals. Statist. Sci., 11(3), 189–228.

Duffy, K. R., Wellard, C. J., Markham, J. F., Zhou, J. H. S., Holmberg, R., Hawkins, E. D., Hasbold, J., Dowling, M. R., & Hodgkin, P. D. (2012). Activation-induced B cell fates are selected by intracellular stochastic competition. Science, 335(6066), 338–341.

Elf, J. & Ehrenbarg, M. (2003). Fast evaluation of fluctuations in biochemical networks with the linear noise approximation. Genome Res., 13, 2475–2484.

Engblom, S. (2006). Computing the moments of high dimensional solutions of the master equation. Appl. Math. Comp., 180, 498–515.

Girolami, M. & Calderhead, B. (2011). Riemann manifold Langevin and Hamiltonian Monte Carlo methods. J. R. Statist. Soc. B, 73(2), 123–214.

Grima, R. (2010). An effective rate equation approach to reaction kinetics in small volumes: Theory and application to biochemical reactions in nonequilibrium steady-state conditions. J. Chem. Phys., 133(035101).

Haario, H., Laine, M., Mira, A., & Saksman, E. (2006). DRAM: Efficient adaptive MCMC. Stat. Comp., 16(4), 339–354.

Hadamard, J. (1902). Sur les problèmes aux dérivées partielles et leur signification physique. In Princeton University Bulletin. 49–52.

Hasenauer, J., Hasenauer, C., Hucho, T., & Theis, F. J. (2014). ODE constrained mixture modelling: A method for unraveling subpopulation structures and dynamics. PLoS Comput. Biol., 10(7), e1003686.

Heinrich, S., Geissen, E., Kamenz, J., Trautmann, S., Widmer, C., Drewe, P., Knop, M., Radde, N., Hasenauer, J., & Hauf, S. (2013). Determinants for robustness in spindle assembly checkpoint signalling. Nature Cell Biology, 15(11), 1328–1339.

Hug, S., Raue, A., Hasenauer, J., Bachmann, J., Klingmüller, U., Timmer, J., & Theis, F. J. (2013). High-dimensional Bayesian parameter estimation: Case study for a model of JAK2/STAT5 signaling. Mathematical Biosciences, 246(2), 293–304.

Hurvich, C. & Tsia, C.-L. (1989). Regression and time series model selection in small samples. Biometrika, 76(2), 297–307.

Joshi, M., Seidel-Morgenstern, A., & Kremling, A. (2006). Exploiting the bootstrap method for quantifying parameter confidence intervals in dynamical systems. Metabolic Eng., 8, 447–455.

Kass, R. E. & Raftery, A. E. (1995). Bayes factors. J. Am. Stat. Assoc., 90(430), 773–795.

Kramer, A., Hasenauer, J., Allgöwer, F., & Radde, N. (2010). Computation of the posterior entropy in a Bayesian framework for parameter estimation in biological networks. In Proc. of the IEEE Multi-Conf. on Syst. and Contr.. Yokohama, Japan, 493–498.

Lee, C. H., Kim, K. H., & Kim, P. (2009). A moment closure method for stochastic reaction networks. J. Chem. Phys., 130(13), 134107.

Lewis, S. M. & Raftery, A. E. (1997). Estimating Bayes factors via posterior simulation with the Laplace-Metropolis estimator. J. Am. Stat. Assoc., 92(438), 648—655.

Meeker, W. Q. & Escobar, L. A. (1995). Teaching about approximate confidence regions based on maximum likelihood estimation. Am. Stat., 49(1), 48–53.

Meng, X. L. & Wong, W. H. (1996). Simulating ratios of normalizing constants via a simple identity: a theoretical exploration. Stat. Sin., 6(4), 831–860.

Murphy, S. A. & van der Vaart, A. W. (2000). On profile likelihood. J. Am. Stat. Assoc., 95(450), 449–485.

Neal, R. M. (2001). Annealed importance sampling. Stat. Comput., 11(2), 125–139.

O'Neill, K., Aghaeepour, N., Špidlen, J., & Brinkman, R. (2013). Flow cytometry bioinformatics. PLoS Comput. Biol., 9(12), e1003365.

Pyne, S., Hu, X., Wang, K., Rossin, E., Lin, T., Maier, L., Baecher-Allan, C., McLachlan, G., Tamayo, P., Hafler, D., De Jager, P., & Mesirov, J. (2009). Automated high-dimensional flow cytometric data analysis. Proc. Natl. Acad. Sci. U S A, 106(21), 8519–8124.

Ramaswamy, R., González-Segredo, N., Sbalzarini, I., & Grima, R. (2012). Discreteness-induced concentration inversion in mesoscopic chemical systems. Nat. Comm., 3(779).

Raue, A., Kreutz, C., Maiwald, T., Bachmann, J., Schilling, M., Klingmüller, U., & Timmer, J. (2009). Structural and practical identifiability analysis of partially observed dynamical models by exploiting the profile likelihood. Bioinf., 25(25), 1923–1929.

Raue, A., Kreutz, C., Theis, F. J., & Timmer, J. (2013). Joining forces of Bayesian and frequentist methodology: A study for inference in the presence of non-identifiability. Phil. Trans. Royal Soc. A, 371(1984).

Skilling, J. (2006). Nested sampling for general Bayesian computation. Bayesian Anal., 1(4), 833–359.

Song, C., Phenix, H., Abedi, V., Scott, M., Ingalls, B. P., Kærn, M., & Perkins, T. J. (2010). Estimating the stochastic bifurcation structure of cellular networks. PLoS Comput. Biol., 6(3), e1000699.

Swain, P. S., Elowitz, M. B., & Siggia, E. D. (2002). Intrinsic and extrinsic contributions to stochasticity in gene expression. Proc. Natl. Acad. Sci. U S A, 99(20), 12795–12800.

van Kampen, N. G. (2007). Stochastic processes in physics and chemistry. Amsterdam: North-Holland, 3rd ed.

Vyshemirsky, V. & Girolami, M. A. (2008). Bayesian ranking of biochemical system models. Bioinf., 24(6), 833–839.

Wilkinson, D. J. (2007). Bayesian methods in bioinformatics and computational systems biology. Briefings in Bioinf., 8(2), 109–116.

Wilks, S. S. (1938). The large-sample distribution of the likelihood ratio for testing composite hypotheses. Ann. Math. Statist., 9(1), 60–62.
