# Supplementary material for: MEMO: multi-experiment mixture model analysis of censored data
Source: Bioinformatics. 2016 Apr 19;32(16):2464–72. doi: 10.1093/bioinformatics/btw190 (PMC4978932; doi:10.1093/bioinformatics/btw190)
Supplement: Supplementary Data [file supp_btw190_MEMO_Supplementary_Material.pdf]

Supplementary Material:
MEMO - Multi-experiment mixture model analysis of censored data

E.-M. Geissen, J. Hasenauer, S. Heinrich, S. Hauf, F.J. Theis and N. Radde

Contents

S.1 Sources of censoring and truncation in single-cell data **2**

S.2 Comparison of selected mixture modeling packages **3**

S.3 Evaluation of MEMO for censored data **4**

S.3.1 Interval censoring . . . 4

S.3.1.1 Inference of population structure . . . 4

S.3.1.2 Inference of population mean . . . 4

S.3.2 Right censoring . . . 4

S.3.2.1 Inference of population structure . . . 5

S.3.2.2 Inference of population mean . . . 5

S.4 Analysis of SAC data **6**

S.4.1 Analysis of wild type data . . . 6

S.4.2 Analysis of microscopy datasets of strains with altered Mad2 abundance . . . 7

S.4.2.1 Analysis of *mad2*Δ data . . . 7

S.4.2.2 Multi-experiment analysis of all Mad2 datasets . . . 7

S.4.3 Analysis of microscopy datasets of strains with altered Mad3 abundance . . . 14

S.4.3.1 Analysis of *mad3*Δ data . . . 14

S.4.3.2 Multi-experiment analysis of all Mad3 datasets . . . 14

S.4.4 Parametrization of distribution parameters . . . 18

S.4.4.1 Parametrization of $w(u)$ to study SAC regulatory mechanisms . . . 19

S.4.4.2 Parametrization of $\mu(u)$ of a log-normally distributed subpopulation with dysfunctional SAC . . . 21

S.5 Analysis of NGF-induced Erk1/2 phosphorylation data **22**

S.5.1 Mechanistic model of cell population . . . 22

S.5.2 Parameter estimation and uncertainty analysis . . . 23

S.6 Case study for right censored and right truncated data with algorithms published in Lee & Scott (2012) **24**

S.1 Sources of censoring and truncation in single-cell data

In the following we provide a list of potential sources of censoring and truncation for different data types. Depending on the experimental setting, these sources of censoring and truncation might not be present or there might be additional sources.

Time-lapse microscopy

- Left and right censoring: Limited time horizon of the experiment.
- Interval censoring: Limited image acquisition rate (technical or to reduce cell stress) and digital signal representation.
- Truncation: Tracking errors or movement of cells out of frame.

Fluorescence microscopy

- Left and right censoring: Background fluorescence and fluorescence saturation. (Visscher et al., 1994)
- Interval censoring: Limited sensitivity and digital signal representation.
- Truncation: Segmentation errors and misclassification of cells.

Flow cytometry

- Left and right censoring: Background fluorescence and fluorescence saturation. (Visscher et al., 1994)
- Interval censoring: Limited sensitivity, automated binning and digital signal representation.
- Truncation: Gating.

Mass cytometry

- Right censoring: Detector saturation. (Nanita, 2013)
- Interval censoring: Discrete ion counts and digital signal representation.
- Truncation: Gating.

Single-cell qPCR

- Left censoring: Detection limit due to limited number of amplification cycles. (Buettner et al., 2014)
- Interval censoring: Digital signal representation.
- Truncation: Gating.

Single-cell RNA-sequencing

- Left and right censoring: Several detection limits. (Shalek et al., 2014)
- Interval censoring: Discrete read counts and digital signal representation.
- Truncation: Gating.

S.2 Comparison of selected mixture modeling packages

In this section we provide Table S1 for a comparison between the provided features of MEMO, FLAME (Pyne et al. (2009)), JCM (Pyne et al. (2014)), BayesFlow (Johnsson et al. (2016)), and the algorithms presented in Lee & Scott (2012).

Table S1: [1]The methods used in FLAME allows for the analysis of univariate data, the implementation does however yield an error with the version available on January 20, 2016. [2]The methods used by Lee & Scott (2012) allows for the analysis of multi-variate data, the comments in the code state however that it is only correctly implemented for uni-directional sampling in each coordinate. In the README it is furthermore stated, that the current implementation considers that the truncation is only on the first coordinate. [3]JCM exploit prior knowledge of the subpopulation structure to perform the inter-condition matching, In general this will not be available. Furthermore, JCM does not allow for a description of the underlying mechanisms and hypothesis testing. [4]Skewed and/or heavy tailed distributions are handled by merging of Gaussian components into super components.

Properties		FLAME	JCM	Lee & Scott (2012)	BayesFlow	MEMO
Censoring	left	-	-	✓	-	✓
	right	-	-	✓	-	✓
	interval	-	-	-	-	✓
	distributed	-	-	-	-	✓
	fitted	-	-	-	-	✓
Truncation	left	-	-	✓	-	-
	right	-	-	✓	-	-
	fitted	-	-	-	-	-
Distributions	normal	-	-	✓	✓[4]	✓
	log-normal	-	-	-	-	✓
	gamma	-	-	-	-	✓
	skew-normal	✓	-	-	-	-
	t	✓	✓	-	-	-
	skew-t	✓	✓	-	-	-
	Johnson SU	-	-	-	-	✓
Data dimension	uni-variate	–[1]	✓	✓	✓	✓
	multi-variate	✓	✓	∼[2]	✓	-
Multi-sample fitting		✓	✓	-	✓	✓
Multi-experiment fitting		-	–[3]	-	-	✓
Simultaneous analysis of all data		-	-	-	-	✓
Automated model selection		-	-	-	-	✓

S.3 Evaluation of MEMO for censored data

To evaluate MEMO and to illustrate the impact of censoring we used MEMO to infer the population structure and distribution parameters from simulated data with known structure and parameters. The evaluation was performed for interval censoring scenarios and right censoring scenarios.

S.3.1 Interval censoring

100 samples were drawn from a single log-normal distribution with parameters $\mu = 1$ and different values for σ. Data were binned according to different censoring intervals Δt to simulate interval censoring, yielding the artificial data. The parameters σ were chosen such that on the main diagonal of the plots in Figure S1 (and of Figure 1B of the manuscript) the ratio between the log-normal standard deviation (calculated from $\mu = 1$ and σ) and the censoring interval Δt equals 0.2. Multi-start local optimization with 30 starts was used to determine the maximum likelihood estimates for models of different complexity.

S.3.1.1 Inference of population structure

Mixture models with 1 to 5 mixture components were fitted to the artificial dataset. The BIC was used to determine the best model. Results are shown in Figure 1B of the manuscript. Color encodes the number of times out of 100 realizations a single log-normal distribution was rated to fit the data best.
We found that, when fitting continuous distributions to interval binned data (Figure 1B, left panel, censoring disregarded) a single distribution can only be identified correctly (regarding the number of subpopulations) as long as all data lie within one bin after applying interval censoring to the data (Figure 1B, right panel, censoring considered), which is likely to be the case if the censoring interval Δt is much larger than the standard deviation of the log-normal distribution. When data are distributed across different bins, single bin values are fitted by a narrow distribution, which leads to overestimation of the number of modes. As we in general do not know a lower limit for the measure of distribution width (σ in our case) of subpopulations, there is no way to prevent this effect. Even when fitting a continuous distribution to continuous data (Δt=0) this effect emerges with increasing width of the distribution (increasing σ). By contrast, taking interval censoring into account by fitting probability mass functions as we propose (Figure 1B, right panel), the single mode of the source distribution can be identified correctly for a wide range of distribution widths and censoring intervals.

S.3.1.2 Inference of population mean

In addition to the population structure the quantification of the properties of the individual subpopulations, e.g., the subpopulation means, can be of interest. To assess the effect of interval censoring on the quality of the estimated mean we compared the results for the two approaches described in Section S.3.1.1. We calculated the means from the estimated parameters of models with a single mode for both approaches. We assessed the relative deviation from the true mean (Figure S1). We find that disregarding interval censoring results in an overestimation of the population mean with increasing censoring interval. This is reflected by lager errors in the estimates for larger censoring intervals Δt (Figure S1, left panel). Taken together with the results in Figure 1B of the manuscript, left panel, these results show that larger Δt are favorable for the correct estimation in terms of the number of subpopulations, but the overall estimate, including the subpopulation parameters, gets worse.

S.3.2 Right censoring

To evaluate the performance of MEMO in the presence of right censoring we used it to infer the distribution structure and parameters from simulated data with known structure and parameters. 200 data points were drawn from a single log-normal distribution with parameters $\mu = 1$ and σ. Data were censored at different times. Parameter σ are chosen such that the censoring value equals the mean at points on the gray curve in Figure 2 in the manuscript. Multi-start local optimization with 30 starts was used to determine the maximum likelihood estimates for models of different complexity. Mixture models with 1 to 5 mixture components were fitted to the dataset and compared by their BIC.

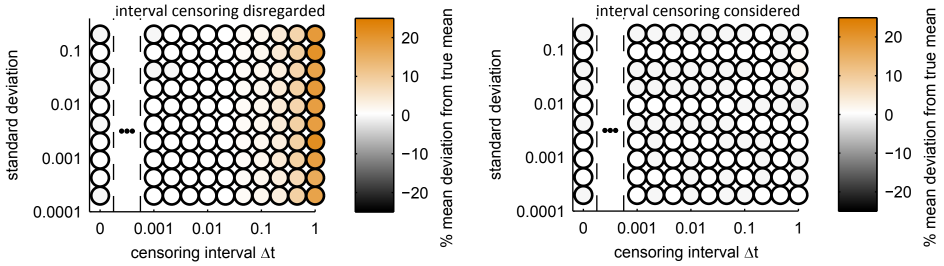

Figure S1: Inference of population structure from interval censored data with and without consideration of interval censoring. Assessment of the accuracy of the estimated subpopulation means. For distributions with different log-standard deviations and different inter-observation intervals Δt, the number of subpopulations is inferred using: (left) A model that does not account for censoring (= standard approach); and (right) a model that accounts for censoring (= MEMO). The mean percentage deviation from the true mean is calculated from the different fits of the 'true' model, namely the model with a single component.

S.3.2.1 Inference of population structure

The results of the model comparison using the BIC are shown in Figure 2 of the manuscript. Color encodes the number of times out of 100 realizations a single log-normal distribution fitted the data best. We considered 3 different scenarios to deal with right censored data. In the first scenario data points beyond the censoring time are omitted and the mixture models are fitted only to the remaining data (manuscript Figure 2B). For distributions with small standard deviations, identification of the single source distribution is possible when the censoring time is greater than the distribution mean. The higher the standard deviation the higher the censoring time has to be to allow correct identification.
In the second scenario data points beyond the censoring value are set to the censoring value (manuscript Figure 2C). This introduces an artificial second mode in the data that leads to the overestimation of the number of mixture components for almost all cases. In scenario number three data points beyond the censoring value are set to the censoring value but modeled separately using MEMO. This approach identifies the single log-normal distribution reliably over the whole range of settings (manuscript Figure 2D).

S.3.2.2 Inference of population mean

Accuracy of estimated means for models with only one mixture component was calculated as percent deviation from the true mean (Figure S2). Discarding censored data or using the value of censoring leads to an underestimation of the population mean, while the approach implemented in MEMO is able to accurately determine the true mean.

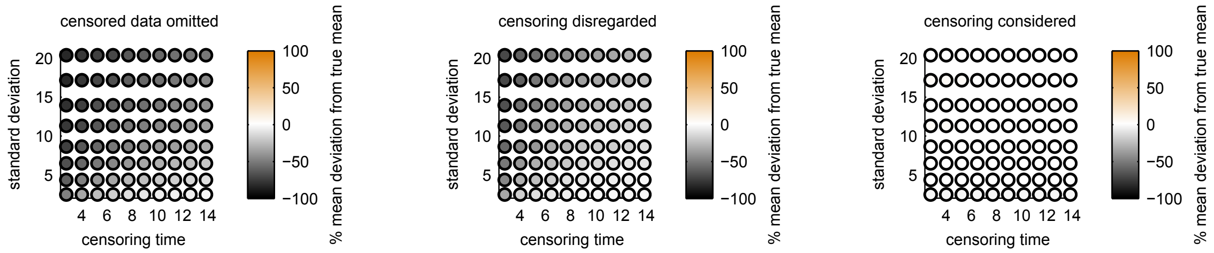

Figure S2: Accuracy of estimated means in the presence of right censoring. Three different approaches were used to fit a model with one to five subpopulations: (Left) A model that does not account for censoring and censored data are disregarded, (center) a model that does not account for censoring and censored data are used, (right) a model that accounts for censoring (= MEMO). The color of the circles encodes the mean percentage deviation from the true mean, calculated from the different fits of the true model, namely the model with a single component.

S.4 Analysis of SAC data

The applicability of MEMO to real experimental data was assessed by studying data collected by (Heinrich et al., 2013). These data provide information about the spindle assembly checkpoint functionality in several yeast strains. The strains differ in the expression of the amount of certain proteins that have a crucial role in SAC functionality. SAC activation in these strains was triggered by preventing microtubule formation with a conditional tubulin mutation (nda3-KM311), and SAC functionality was assessed by measuring the time individual cells stayed in prometaphase, an early phase in mitosis. Measurement was performed by time lapse video microscopy. The individual cells were recorded every 5 minutes for 17 hours, leading to datasets containing interval and right-censored data. We denote the interval censored data with "prometaphase lengths" and the right censored data with "censoring times". Since cells enter prometaphase asynchronously, the censoring times are distributed.
Our aim was to analyze the data obtained for each strain with respect to the number and identity of subpopulations differing in their SAC activity and to quantify the cell-to-cell variability in terms of the distribution of the data points. For the sake of simplicity we analyzed the datasets of strains with altered abundance of Mad2 and Mad3 separately, although it would be possible to analyze all datasets simultaneously with MEMO. General conclusions drawn from the analysis for Mad2 and Mad3 datasets are very much alike, but we provide the results for both datasets for the sake of completeness.

S.4.1 Analysis of wild type data

To demonstrate the relevance of considering right censoring in the analysis of biological data, we analyzed the population structure of a dataset for a wild type strain. The dataset is shown in Figure S3 and in Figure 5A in the manuscript together with all other Mad2 datasets. We analyzed the dataset with respect to the number of subpopulations in three different scenarios of treating the right censored data. Interval censoring resulting from 5 min inter-observation time was considered in all three cases. Since yeast strains are clonal populations of cells, i.e. genetically identical, and since the SAC is a crucial surveillance mechanism, we do not expect subpopulations with qualitatively different SAC functionality within this wild type dataset.
The distribution of prometaphase lengths was modeled by a log-normal distribution, or a mixture of two log-normal distributions. Censoring times were modeled by a Johnson-SU distribution. By comparing a model with one mixture component with a model with two mixture components via their BICs, we show for each scenario how many subpopulations are predicted. In the first scenario censored data were completely omitted and only data of cells with observed end of prometaphase were used. As shown in Figure S3 it is not possible to decide whether the dataset contains one or two subpopulations, since the BIC does not differ substantially between the two hypotheses. In the second scenario censoring times are treated as prometaphase lengths. Figure 4C of the manuscript shows that this leads to the spurious identification of two subpopulations. In the third scenario, when using MEMO to model prometaphase lengths and censoring times to be generated by competing processes, the single subpopulation is correctly identified. Moreover, the unobserved overall distribution of prometaphase lengths can be reconstructed (manuscript Figure 4D).

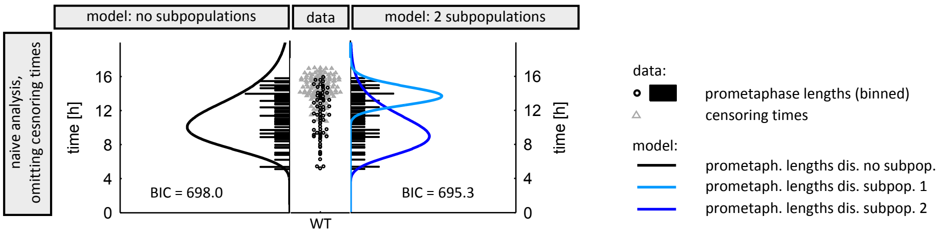

Figure S3: Scenario 1: Analysis of the population structure of a wild type (WT) dataset when omitting right censored data points and exclusively using prometaphase lengths. Circles and black bars indicate cells in which the entire prometaphase was recorded (prometaphase lengths, interval censored data). Triangles indicate cells that were still in prometaphase when recording stopped (censoring times, right censored data). Lines depict models with one (left) or two (right) subpopulations fitted to the data.

S.4.2 Analysis of microscopy datasets of strains with altered Mad2 abundance

The distribution of prometaphase lengths was modeled by a log-normal distribution or mixtures of those, whereas the distribution of censoring times was modeled by a Johnson SU distribution. The log-normal distribution was chosen over the Johnson SU distribution because it yielded a smaller BIC (as shown below in Figure S5). The analysed dataset includes in total nine different experimental conditions (nine different Mad2 protein levels) and 934 quantified single cells. The optimization for a single model hypothesis in this example is relatively fast (< 10 s on average per start in the multi-start optimization).

S.4.2.1 Analysis of *mad2*Δ data

To assess the relevance of considering interval censoring we analyzed the population structure of the *mad2*Δ strain. This strain is lacking the gene encoding for Mad2. The lack of Mad2 results in a dysfunctional SAC (Li & Murray, 1991), hence, subpopulations with different SAC functionality are not expected in this dataset. The dataset is shown in Figure S4 and in Figure 5A of the manuscript together with all datasets with altered Mad2 abundance. The dataset solely consists of cells with observed ends of prometaphase. The data are interval censored as microscopy images are only taken every 5 min. We analyzed the dataset with respect to the number of subpopulations in two different scenarios. In the first scenario the fact that the data is interval censored is neglected. In the second case the observation interval of 5 minutes is considered using MEMO. We compared a model with one mixture component with a model with two mixture components by the BIC of their MLEs. We find that the naive analysis selects a statistical model with two subpopulations (see manuscript, Fig.4A), while MEMO selects a model with a single population (see manuscript, Fig.4B). Biologically no subpopulation structure is expected as all *mad2*Δ cells have a non-functional SAC, therefore we conclude that consideration of interval censoring can be important to gain meaningful insights in the population structure.

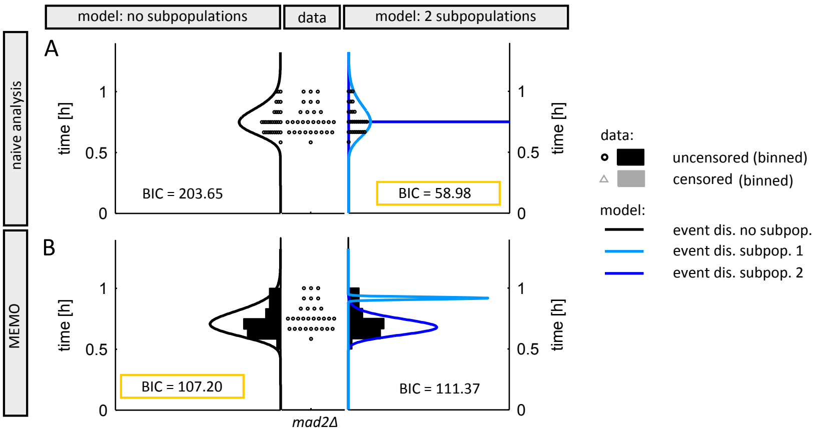

Figure S4: Analysis of the population structure of the *mad2*Δ dataset: Interval censoring has to be considered for accurate reconstruction of SAC functionality from fluorescence live-cell microscopy imaging. For the *mad2*Δ strain (dysfunctional SAC) unimodal prometaphase lengths are observed. Circles and black bars indicate cells in which the entire prometaphase was recorded (prometaphase lengths). We used the BIC to decide upon the number of subpopulations for different settings. (A) Naive analysis selects a statistical model with two subpopulations, while (B) MEMO selects a model with a single population.

S.4.2.2 Multi-experiment analysis of all Mad2 datasets

MEMO was used to analyze the qualitative and quantitative properties of the population structure for all datasets of strains with altered abundances of the protein Mad2. All datasets are shown in Figure 5A of the manuscript. In the following we present the results of the simultaneous analysis of the subpopulation structure in all datasets. We visualize the agreement between model and data and the model uncertainties.

Hypothesis testing reveals the existence and nature of subpopulations. As described in the manuscript we assessed the population structure of the different strains with altered abundance of Mad2 in

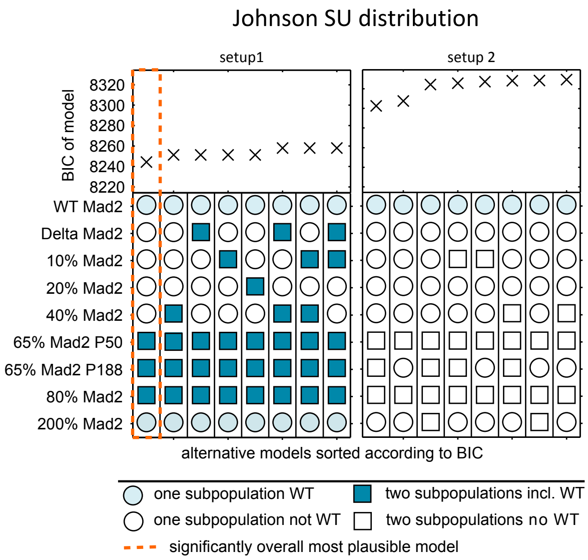

Figure S5: Hypothesis testing via automated backward model selection for strains with altered Mad2 abundance. Comparison of the results of two initial model setups for the prometaphase length distributions: setup 1 – weighted mixture of one wild-type (WT) and one strain specific distribution; and setup 2 – weighted mixture of two strain-specific distributions. Each column illustrates an alternative model characterized by its subpopulation structure. For each setup, the structures of the eight most plausible models were recorded, ranked according to their BIC. Prometaphase lengths were modeled by mixtures of Johnson SU distributions. Censoring times were modeled by a single Johnson SU distribution.

a hypothesis-driven approach. Therefore, two setups were compared by their BICs:

- setup 1: the initial model assumes two mixture components for the distribution of prometaphase lengths of each strain (except wild type), in which one component, e.g. subpopulation is equal to the wild type distribution while the other component/subpopulation is strain-specific.
- setup 2: the initial model assumes two subpopulations in which both mixture components/subpopulations are strain-specific (and unlike the wild type distribution).

Prometaphase lengths were modeled by log-normal distributions. Censoring times were modeled by a Johnson SU distribution and considered to be equal in all strains. The overall most plausible model was determined via automated backward model selection as described in Section D.2.4.2 in the MEMO Documentation. The full optimization problem for this particular model selection study comprises testing of $3^8 = 6561$ model variants, when considering the identity of the two possible subpopulations. Via the implemented backward selection procedure the number of tested model variants was reduced to 42. In this setting this corresponded to a runtime of 85 min.
We could confirm the existence of two subpopulations in three strains (both 65% Mad2 strains and the 80% Mad2 strain) which consist of one strain specific subpopulation and a wild type subpopulation. The 200% Mad2 strain was identified to be statistically identical to the wild type, and the *mad2*Δ strain, the 10%, the 20% and the 40% Mad2 strains were identified to be a single subpopulation substantially different from the wild type (Figure 5B in the manuscript). These findings were confirmed assuming the prometaphase lengths to be Johnson SU distributed (Figure S5). Since the BIC of the most plausible model assuming log-normal distributions is lower, we conclude that the log-normal distribution is suitable to describe prometaphase lengths in the Mad2 SAC datasets. This model is referenced as optimal model in the following, and all further analyses and visualization in this section refers to this model. Figure 5C of the manuscript shows the estimated distributions for the optimal model. The parameters of the optimal model for the subpopulation structure of the Mad2 datasets are provided in Figure S6 using the output function `printmodel.m` of MEMO.

Type of mixture: log-normal

Dataset	component 1			component 2		
	w	mu	sigma	w	mu	sigma
60% Mad2 P50	0.44	6.85	0.43	0.56	4.51	0.34
60% Mad2 P188	0.80	6.85	0.43	0.20	4.90	0.32
80% Mad2	0.82	6.85	0.43	0.18	4.74	0.35
200% Mad2 P(259bp)	1.00	6.85	0.43			
Delta Mad2				1.00	3.77	0.14
10% Mad2				1.00	3.81	0.15
20% Mad2				1.00	3.97	0.15
40% Mad2				1.00	4.09	0.16
WT Mad2	1.00	6.85	0.43			

Number of parameters: 23
log-posterior: -4.0164e+03
AIC: 8.0788e+03
BIC: 8.1901e+03
Likelihood ration: Lambda = 3.9484e-02, p-value = 4.8676e-01

Type of mixture: Johnson SU

Dataset	component 1				
	w	gamma	sigma	lambda	xi
60% Mad2 P50	1.00	11.57	2.61	5.27	1100.96
60% Mad2 P188	1.00	11.57	2.61	5.27	1100.96
80% Mad2	1.00	11.57	2.61	5.27	1100.96
200% Mad2 P(259bp)	1.00	11.57	2.61	5.27	1100.96
Delta Mad2	1.00	11.57	2.61	5.27	1100.96
10% Mad2	1.00	11.57	2.61	5.27	1100.96
20% Mad2	1.00	11.57	2.61	5.27	1100.96
40% Mad2	1.00	11.57	2.61	5.27	1100.96
WT Mad2	1.00	11.57	2.61	5.27	1100.96

Number of parameters: 23
log-posterior: -4.0164e+03
AIC: 8.0788e+03
BIC: 8.1901e+03
Likelihood ration: Lambda = 3.9484e-02, p-value = 4.8676e-01

Figure S6: Parameters of the overall most plausible population structure from the analysis of the Mad2 datasets. Model for prometaphase lengths (top), model for censoring times (bottom).

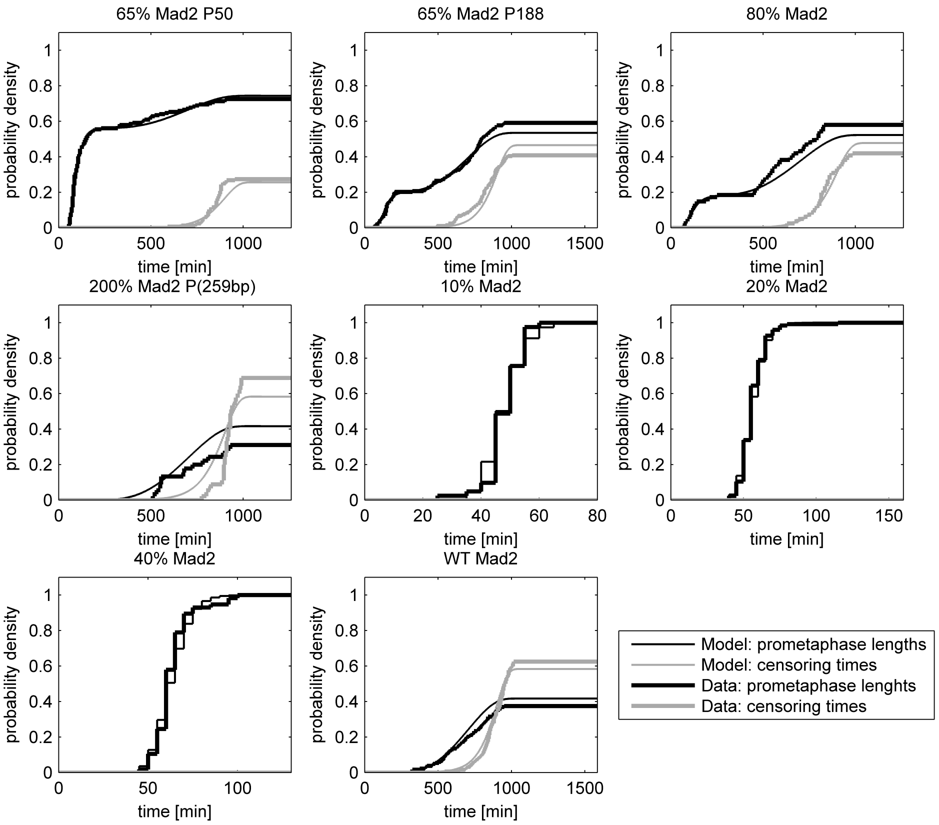

Figure S7: Model data comparison for a model which explicitly models the distribution of censoring times. Thick lines show data, thin lines show the cumulative distributions of the conditional probability densities of prometaphase lengths and censoring times resulting from the model.

Model-data comparison. Figure S7 shows the comparison of model and data for the optimal model which explicitly models the distribution of censoring times. To facilitate a direct comparison of mixture model and data (without further preprocessing of the data) we plot the cumulative distribution of prometaphase lengths and censoring times.
In many research projects no explicit model for the censoring distribution is considered. Instead, the likelihood of the censored data is set to the tail probability (see Section D.2.1.3 in the Documentation). The corresponding results are depicted in Figure S8. While comparable parameter estimates are obtained, the model-data comparison is not straight forward. If the predicted distribution is plotted it diverges from the measured one for late time points since the distribution including censoring is not accessible.

Goodness of fit. In addition to the visual model-data comparison the goodness of the optimal model was assessed via bootstrapping the likelihood function (Figure S9). Therefor different datasets were generated via simulation from the optimal model, and the likelihood value of these datasets was evaluated. The log-likelihood for the experimental data (red circle) is within the 90 % confidence interval, showing that the model is plausible.

Uncertainty/Identifiability analysis. To assess the identifiability of the model parameters and the uncertainty of the parameter estimates, we performed a profile likelihood analysis and MCMC sampling. For the MCMC sampling we assumed a prior which is constant between the lower and upper bounds of the parameters and zero outside. Accordingly MLE and MAP agree. The results of the uncertainty analysis of the optimal model for both approaches are shown in Figure S10. Almost all parameters are identifiable and uncertainties are small. The means, standard deviations and the 95% percentile interval resulting from MCMC sampling are shown in Table S2. Figure S11 shows the comparison of measurement data and model, including the uncertainty of the model predictions. We find that the data are contained in the computed confidence intervals, supporting the validity of the model.

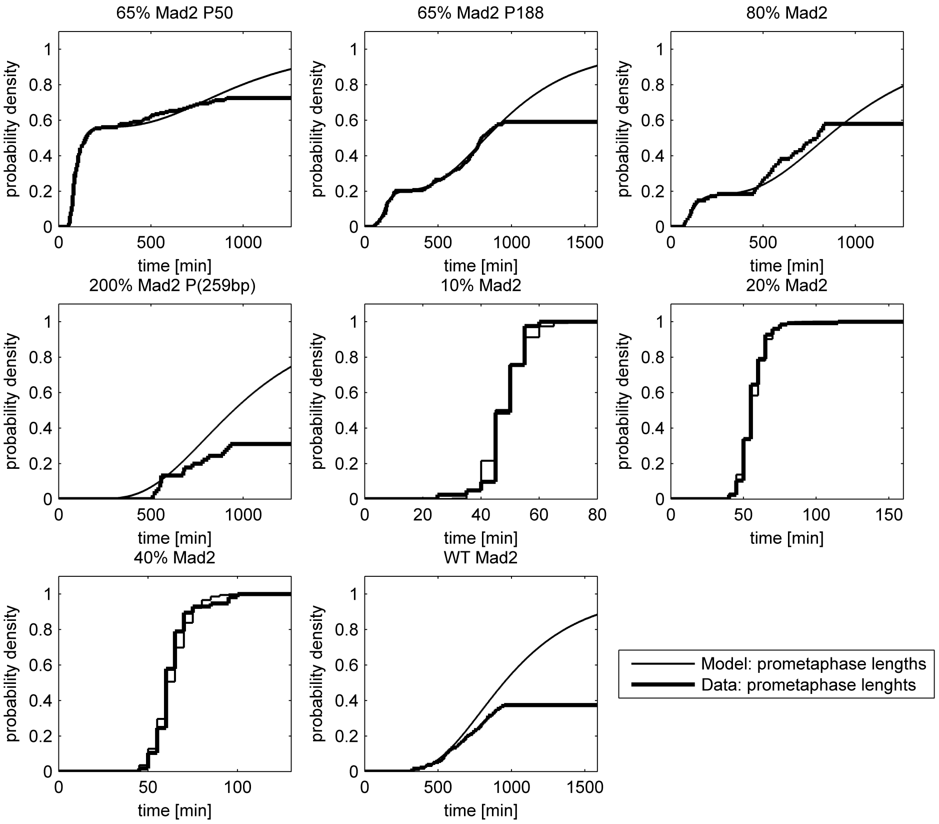

Figure S8: Model data comparison for a model without an explicit model for the censoring times. Thick lines show data, thin lines show the model. Since this model does not describe the censoring its cumulative distribution does not resemble the data for later time points where the data is dominated by censoring times.

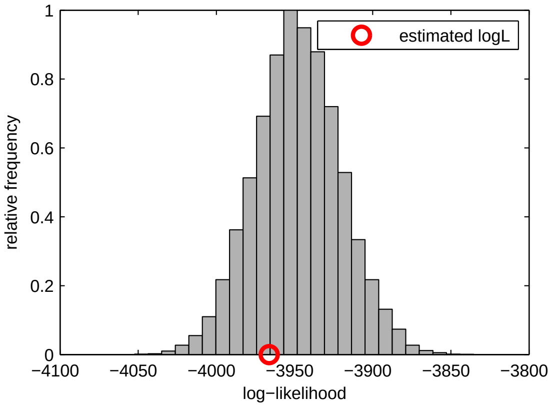

Figure S9: Comparison of log-likelihood of the experimental data (red circle) with bootstraps from the distribution.

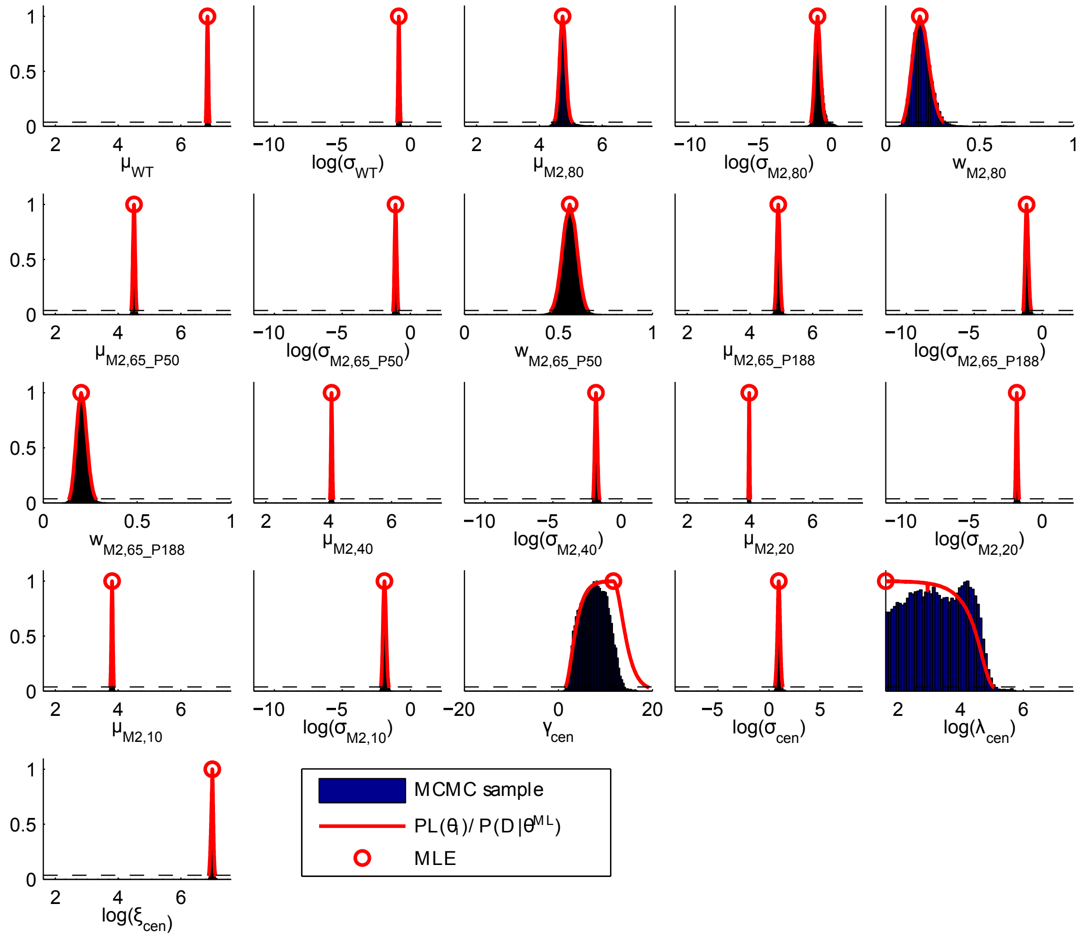

Figure S10: Profile likelihoods and marginal distributions of MCMC sampling for the parameters of the overall best model of the analysis of the Mad2 datasets (model in leftmost column in Figure 5B in the manuscript.

parameter name	mean	std	2.5 percentile	97.5 percentile
μ_c	6.851	0.026079	6.803	6.9065
$\log(\sigma_c)$	-0.84057	0.056523	-0.9465	-0.7249
$\mu_{M2,80}$	4.7547	0.16344	4.5165	5.0833
$\log(\sigma_{M2,80})$	-0.92626	0.27188	-1.3766	-0.2782
$w_{M2,80}$	0.1937	0.051134	0.1134	0.2968
$\mu_{M2,65\_P50}$	4.5058	0.036806	4.4343	4.5795
$\log(\sigma_{M2,65\_P50})$	-1.0762	0.080227	-1.227	-0.9139
$w_{M2,65\_P50}$	0.55945	0.038675	0.4846	0.6349
$\mu_{M2,65\_P188}$	4.9048	0.053405	4.8009	5.0119
$\log(\sigma_{M2,65\_P188})$	-1.1144	0.12311	-1.3429	-0.8568
$w_{M2,65\_P188}$	0.20599	0.027587	0.1546	0.2626
$\mu_{M2,40}$	4.0917	0.021374	4.0493	4.1328
$\log(\sigma_{M2,40})$	-1.8254	0.09846	-2.0085	-1.6254
$\mu_{M2,20}$	3.9746	0.014684	3.945	4.0034
$\log(\sigma_{M2,20})$	-1.8593	0.06528	-1.9843	-1.7279
$\mu_{M2,10}$	3.8058	0.02458	3.7579	3.8533
$\log(\sigma_{M2,10})$	-1.8795	0.11815	-2.1023	-1.6406
γ_{cen}	7.6208	2.7881	2.7343	12.7188
$\log(\sigma_{cen})$	0.96818	0.13604	0.7147	1.2546
$\log(\lambda_{cen})$	3.2428	0.91177	1.7047	4.7636
$\log(\xi_{cen})$	7.0023	0.031556	6.9397	7.0711

Table S2: Means, standard deviations and 95% percentile intervals of the parameters resulting from MCMC sampling of the optimal model of the Mad2 datasets.

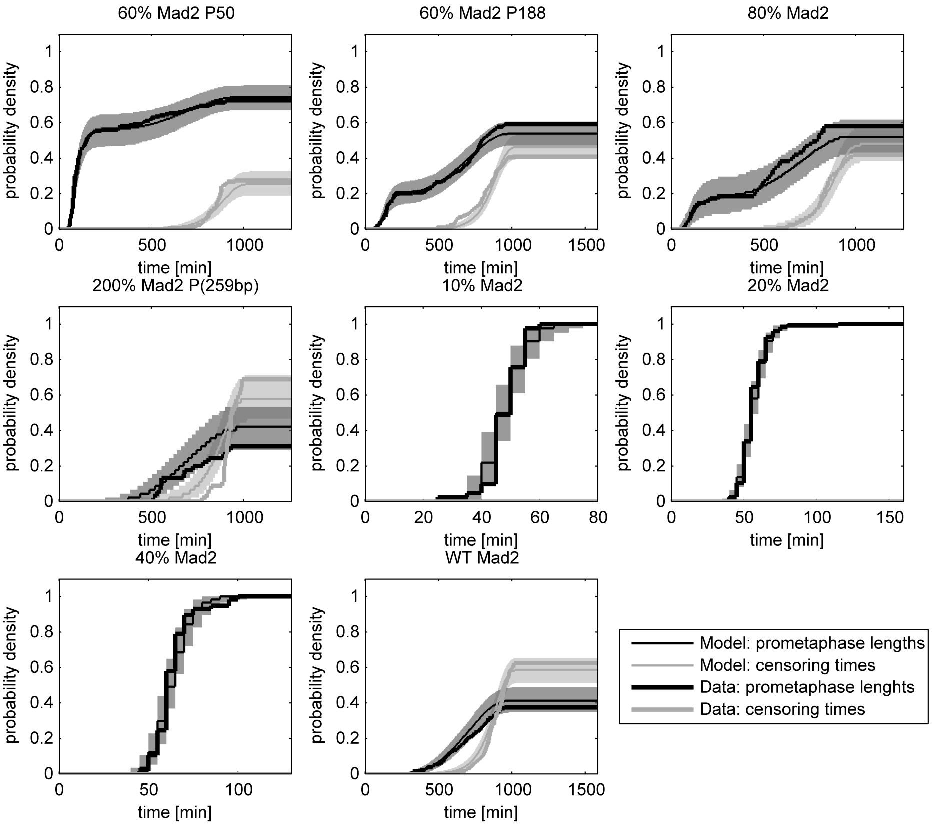

Figure S11: Comparison of measurement data and estimated mixture model for the overall most plausible model of the Mad2 datasets. To facilitate a direct comparison of mixture model and data (without further preprocessing of the data) the cumulative distribution of prometaphase lengths and censoring times are plotted. The distribution of the prometaphase lengths is shown in black, while the distribution of censoring times is shown in gray. The thin line represents the median prediction and the thick line represents the measurement data. Both distributions are scaled with the relative frequency of the event. The light intervals are the 95% confidence intervals of the prediction.

S.4.3 Analysis of microscopy datasets of strains with altered Mad3 abundance

The distribution of prometaphase lengths was modeled by a log-normal distribution or mixtures of those, whereas the distribution of censoring times was modeled by a Johnson SU distribution. The log-normal distribution was chosen since testing against the Johnson SU distribution yielded a smaller BIC for models with the log-normal distribution compared to models with Johnson SU distribution (as shown below in Figure S13).

S.4.3.1 Analysis of *mad3*Δ data

The *mad3*Δ strain lacks the gene encoding for Mad3. The lack of Mad3 results in a dysfunctional SAC (Li & Murray, 1991), hence, subpopulations with different SAC functionality are not expected in this dataset. The dataset is shown in Figure 4 of the manuscript and in Figure S12A together with all datasets with altered Mad3 abundance.The dataset solely consists of cells with observed ends of prometaphase. The data are interval censored as microscopy images are only taken every 5 min. To assess the influence of the interval censoring on the analysis outcome we used two analysis approaches. In the first approach the fact that data is interval censored is neglected. In the second case the observation interval of 5 minutes is considered using MEMO. With both approach we analyzed whether the population structure of the dataset consists of one or of two subpopulations. The optimal models achieved for both approaches and their BICs are depicted in Figure 4 of the manuscript. As already observed for the *mad2*Δ dataset, we find that the naive analysis selects a statistical model with two subpopulations, while MEMO selects a model with a single population. Biologically, no subpopulation structure is expected as all *mad3*Δ cells have a non-functional SAC, therefore this result further strengthens the importance of considering interval censoring in the analysis of population data.

S.4.3.2 Multi-experiment analysis of all Mad3 datasets

MEMO was used to analyze the qualitative and quantitative properties of the population structure for all datasets of strains with altered abundance of protein Mad3 (data shown in Figure S12A).

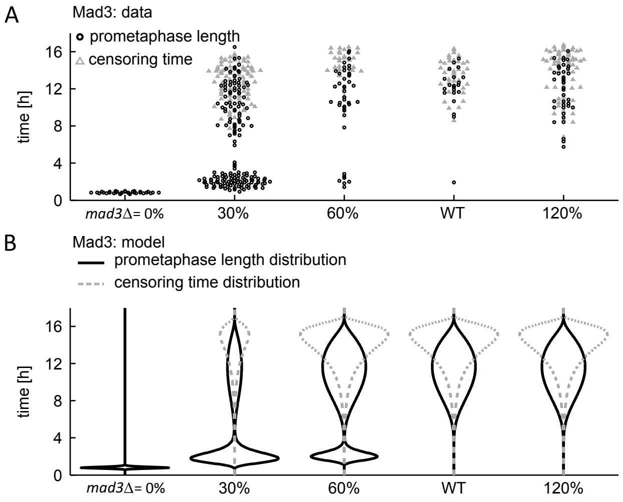

Figure S12: Data and MEMO derived distributions for datasets of strains with altered Mad3 abundance. (A) Measured prometaphase lengths for *S. pombe* strains with different Mad3 abundance. Circles indicate cells in which the entire prometaphase was recorded (prometaphase lengths, interval censored). Triangles indicate cells that were still in prometaphase when recording stopped (censoring times, right censored). Since cells enter prometaphase asynchronously, the times at which data are censored are distributed. (B) Fit of distributions for prometaphase lengths (black lines) and censoring times (dashed gray lines) for the overall most plausible model selected by MEMO. To mimic the bee swarm plots in (A) probability densities are vertically mirrored.

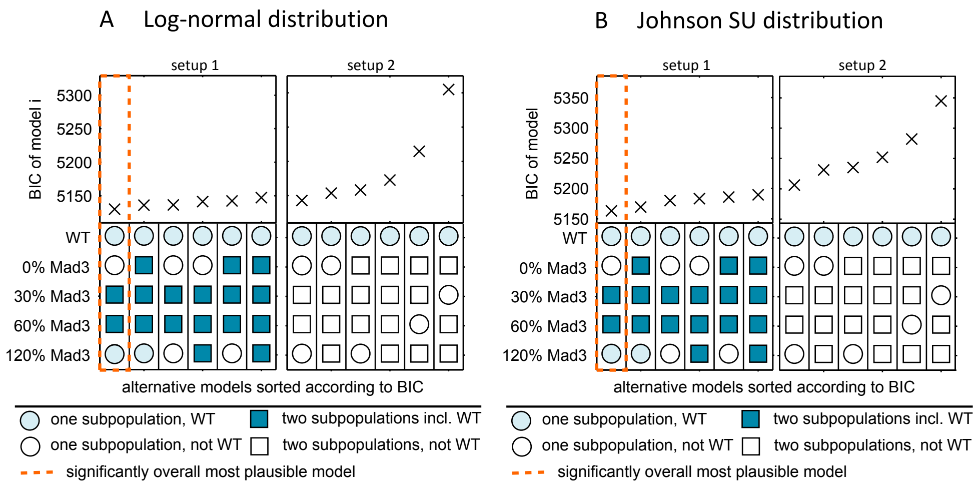

Figure S13: Hypothesis testing via automated backward model selection for strains with altered Mad3 abundance. Comparison of the results of two initial model setups for the prometaphase length distributions: setup 1 – weighted mixture of one wild-type and one strain specific distribution; and setup 2 – weighted mixture of two strain-specific distributions. Each column illustrates an alternative model characterized by its subpopulation structure. For each setup, the structures of the eight most plausible models were recorded, ranked according to their BIC. (A) Prometaphase lengths were modeled by mixtures of log-normal distributions. (B) Prometaphase lengths were modeled by mixtures of Johnson SU distributions. Censoring times were modeled by a single Johnson SU distribution in both setups.

Hypothesis testing reveals the existence and nature of subpopulations. We performed the same analysis for the Mad3 datasets as described in manuscript for the datasets of Mad2 alterations. Therefore two setups were compared by their BICs:

- setup 1: the initial model assumes two mixture components for the distribution of prometaphase lengths of each strain, in which one component, e.g. subpopulation is equal to the wild type distribution while the other component/subpopulation is strain-specific.
- setup 2: the initial model assumes two subpopulations in which both mixture components/subpopulations are strain-specific (and unlike the wild type distribution).

The optimal models of both setups were compared by their BICs. We could confirm the existence of two subpopulations in two strains (30% Mad3 and 60% Mad3), which consist of one strain specific and the wild type subpopulation. The 120% Mad3 strain was identified to be statistically identical to the wild type, and the *mad3*Δ strain was identified to be a single subpopulation different from the wild type (Figure S13A). These findings were confirmed assuming the prometaphase lengths to be Johnson SU distributed (Figure S13B). Since the BIC of the most plausible model assuming log-normal distributions is lower compared to when assuming Johnson SU distributions, we conclude that the log-normal distribution is suitable to describe prometaphase lengths in the Mad3 SAC datasets. Figure S12B shows the estimated distributions for the overall most plausible model. The estimated parameters for this model are shown in Figure S14.

Uncertainty/identifiability analysis of model parameters and model data comparison. To assess the identifiability of the model parameters and the uncertainty of the parameter estimates we performed a profile likelihood analysis and MCMC sampling. For the MCMC sampling we assumed a prior which is constant between lower and upper bounds of the parameters and zero outside. Accordingly MLE and MAP agree. The results of the uncertainty analysis of the optimal model for both approaches are shown in Figure S15. Almost all parameters are identifiable and uncertainties are small. The means, standard deviations and the 95% percentile interval resulting from MCMC sampling are shown in Table S3. Figure S16 shows the comparison of measurement data and model, including the uncertainty of the model predictions. We find that the data are contained in the computed confidence intervals, supporting the validity of the model.

```
Type of mixture: log-normal
 ----------------------------------------------------------
| Dataset      |     component 1      |     component 2      |
|--------------|----------------------|----------------------|
|              |  w   |  mu  | sigma |  w   |  mu  | sigma |
|--------------|----------------------|----------------------|
| Delta Mad3   |      |      |       | 1.00 | 3.86 |  0.11 |
| 30%  Mad3    | 0.63 | 6.79 |  0.38 | 0.37 | 4.77 |  0.30 |
| 60%  Mad3    | 0.90 | 6.79 |  0.38 | 0.10 | 4.83 |  0.22 |
| 120%  Mad3   | 1.00 | 6.79 |  0.38 |      |      |       |
| WT           | 1.00 | 6.79 |  0.38 |      |      |       |
 ----------------------------------------------------------

Number of parameters: 14
log-posterior: -2.5216e+03
AIC: 5.0711e+03
BIC: 5.1300e+03
Likelihood ration: Lambda = 2.4839e-02, p-value = 1.1663e-01


Type of mixture: Johnson SU
 --------------------------------------------------------
| Dataset      |               component 1                |
|--------------|------------------------------------------|
|              |  w   | gamma | sigma | lambda |   xi     |
|--------------|------------------------------------------|
| Delta Mad3   | 1.00 |  7.62 |  1.79 |   5.08 | 1053.12 |
| 30% Mad3 (2) | 1.00 |  7.62 |  1.79 |   5.08 | 1053.12 |
| 60% Mad3     | 1.00 |  7.62 |  1.79 |   5.08 | 1053.12 |
| 120% Mad3    | 1.00 |  7.62 |  1.79 |   5.08 | 1053.12 |
| WT           | 1.00 |  7.62 |  1.79 |   5.08 | 1053.12 |
 --------------------------------------------------------

Number of parameters: 14
log-posterior: -2.5216e+03
AIC: 5.0711e+03
BIC: 5.1300e+03
Likelihood ration: Lambda = 2.4839e-02, p-value = 1.1663e-01
```

Figure S14: Parameters of the overall most plausible population structure from the analysis of the Mad3 datasets. Model for prometaphase lengths (top), model for censoring times (bottom).

parameter name	mean	std	2.5 percentile	97.5 percentile
μ_c	6.7968	0.026231	6.7478	6.8511
$log(\sigma_c)$	-0.96439	0.060801	-1.0797	-0.8405
$\mu_{M3,60}$	4.8384	0.15601	4.6158	5.0903
$log(\sigma_{M3,60})$	-1.3259	0.35757	-1.8738	-0.5183
$w_{M3,60}$	0.11413	0.038459	0.0501	0.1996
$\mu_{M3,30}$	4.7691	0.033269	4.7044	4.8347
$log(\sigma_{M3,30})$	-1.1812	0.08025	-1.3315	-1.0174
$w_{M3,30}$	0.36869	0.031394	0.3089	0.431
$\mu_{M3,0}$	3.8577	0.021173	3.8153	3.899
$log(\sigma_{M3,0})$	-2.1525	0.10535	-2.2954	-1.9046
γ_{cen}	5.9454	2.0389	2.6855	10.395
$log(\sigma_{cen})$	0.64285	0.14894	0.3908	0.985
$log(\lambda_{cen})$	2.8699	0.76965	1.6731	4.3608
$log(\xi_{cen})$	6.9697	0.029478	6.9276	7.0443

Table S3: Means, standard deviations, and 95% percentile intervals of the parameters resulting from MCMC sampling of the optimal model of the Mad3 datasets.

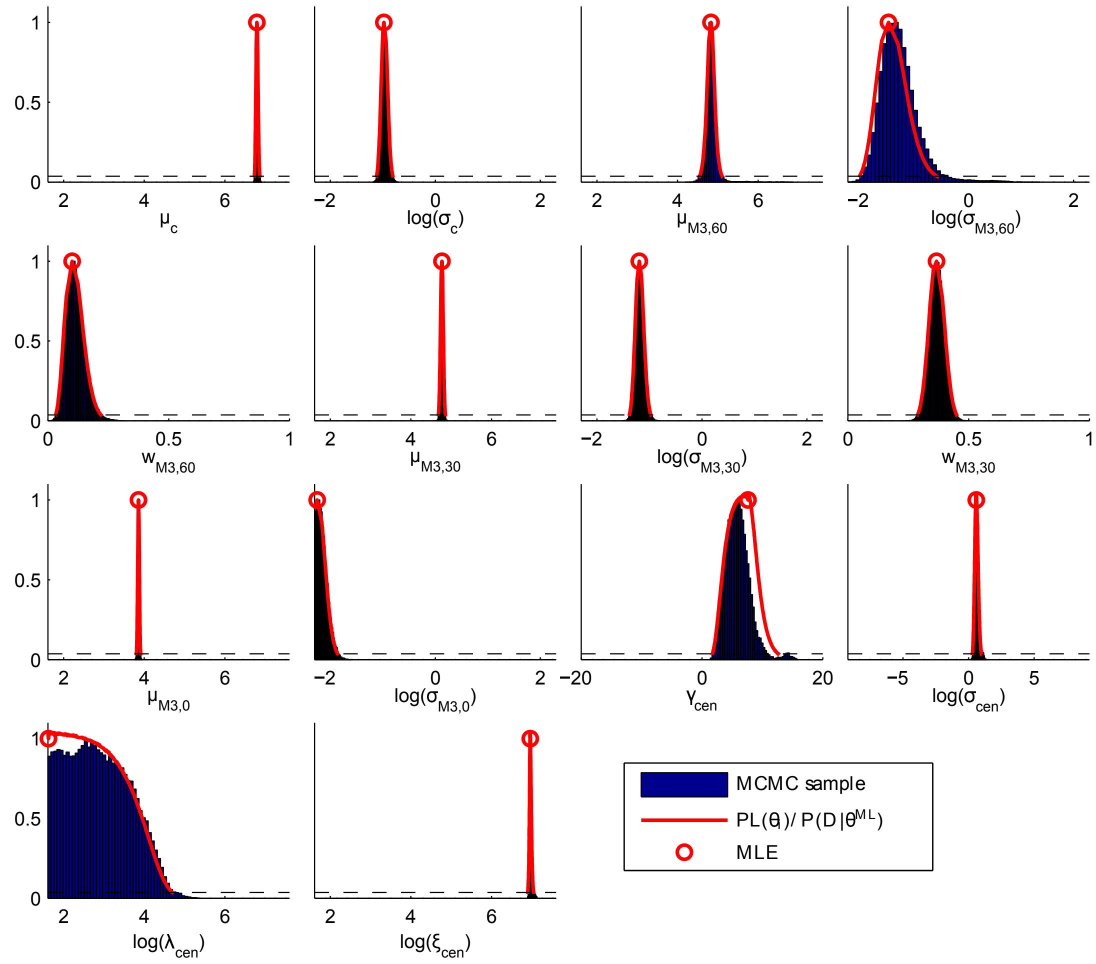

Figure S15: Profile likelihoods and marginal distributions of MCMC sampling for the parameters of the overall best model of the analysis of Mad3 datasets (model in leftmost column in Figure S13).

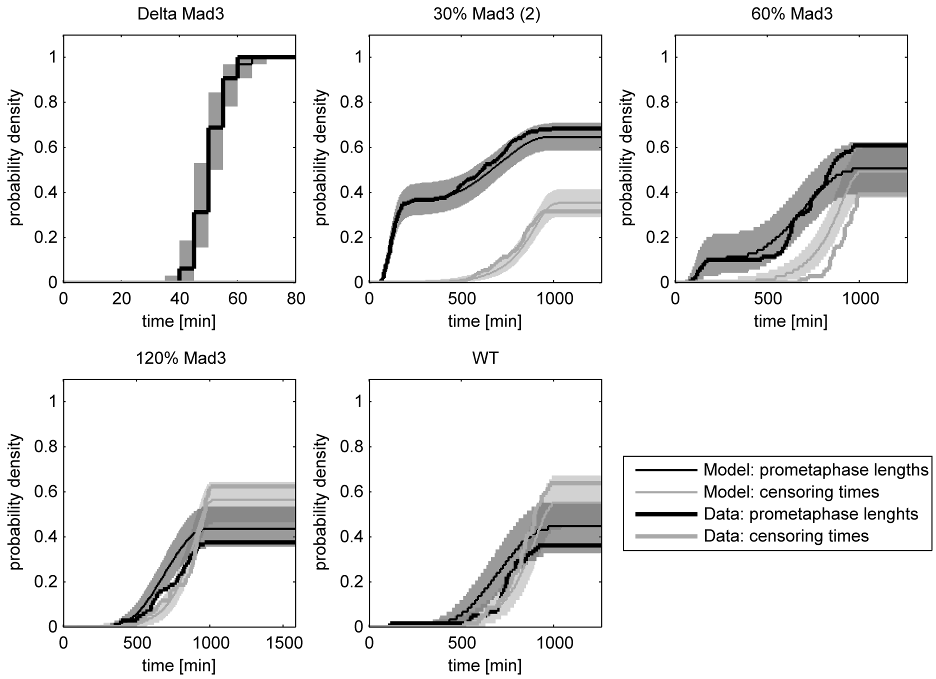

Figure S16: Comparison of measurement data and estimated mixture model of the most plausible model for the Mad3 datasets. To facilitate a direct comparison of mixture model and data (without further preprocessing of the data) the cumulative distribution of prometaphase lengths and censoring times are plottet. The distribution of the prometaphase lengths is shown in black, while the distribution of censoring times is shown in gray. The thin line represents the median prediction and the thick line represents the measurement data. Both distributions are scaled with the relative frequency of the event. The light intervals are the 95% confidence intervals of the prediction.

S.4.4 Parametrization of distribution parameters

MEMO can also deal with a further layer of parametrization, namely the parametrization of the parameters of the mixture models. This means that distribution parameters can be functions of the input and so called meta-parameters (see D.2.2 in the Documentation). This feature enables us to test/include different hypotheses of input dependency to improve performance.
To analyze SAC datasets independently of the error-prone protein concentrations of different strains, we did not use this parametrization in the first place. In the following we want to show how parameter parametrization can be applied. We will show parametrization of the fraction $w(u)$ of cells with functional (= wild type-like) SAC and for parameter $\mu(u)$ of the log-normal mixture component representing the subpopulation of cells with dysfunctional SAC.
In the SAC dataset the input is given by the protein amount relative to the amount in the wild type strain. To account for the uncertainty in the protein amount, which was determined via immunoblotting, which has technical challenges (described in (Heinrich et al., 2013), we included the measurement error of the input in the optimization problem. We assume the error to be additively normally distributed. For every experimental condition, with the exception of the Δ-strains and the wild type, an additional error parameter has to be defined together with its σ (for example see model definition file `model_Mad2_all_fun_mu_hill.m`). Please note that the automated backward model selection routine (see Section D.2.4.1 in the Documentation) cannot deal with parametrized mixture parameters in the current version of MEMO.

S.4.4.1 Parametrization of $w(u)$ to study SAC regulatory mechanisms

We modeled the fraction of cells with a fully functional (= wild type-like) SAC for different Mad2 and Mad3 abundances with Hill-type functions:

$$WT_{fracM2}(n_2, K_2, u_{Mad2_{rel}}, \epsilon_{Mad2}) = (1 + K_2^{n_2}) \frac{(u_{Mad2_{rel}} + \epsilon_{Mad2})^{n_2}}{(u_{Mad2_{rel}} + \epsilon_{Mad2})^{n_2} + K_2^{n_2}}, \quad (1)$$

$$WT_{fracM3}(n_3, K_3, u_{Mad3_{rel}}, \epsilon_{Mad3}) = (1 + K_3^{n_3}) \frac{(u_{Mad3_{rel}} + \epsilon_{Mad3})^{n_3}}{(u_{Mad3_{rel}} + \epsilon_{Mad3})^{n_3} + K_3^{n_3}} \quad (2)$$

with measurement errors ϵ_{Mad2} and ϵ_{Mad3} respectively, which are different for the different conditions. Input and wild type-like fraction are correlated positively. The functions were normalized, so that they take the value 1 for input values ≥ 1. The model with the parametrized fraction was fitted to all Mad2 and Mad3 datasets simultaneously.
The integration of the parametrization into the model definition file is simple. Parameters used for parametrization, from here on called meta-parameters, have to be defined in the same way as all other parameters, and the parameter to be parametrized is just exchanged by the respective function of the meta-parameters and the input. The meta-parameters are estimated together with the mixture parameters and the error parameters.
The parameter values for the MLE are shown in Figure S17. Figure 6A in the manuscript shows the functional dependency of w over u for Mad2 and Mad3 resulting from the best fit of the model together with the respective data. The fraction of wild type-like cells for Mad2 or Mad3 concentrations lying between the ones that have been experimentally tested can be calculated from (1) and (2), and assessed visually from the figures. For the Mad2 datasets the wild type fraction shows a switch like behavior, indicating an ultra-sensitive dependency of the fraction of the wild type subpopulation on the amount of Mad2. Changes in Mad3 have a more graded effect on the wild type fraction.

Type of mixture: log-normal

Dataset	component 1			component 2			input dependency						
	w	mu	sigma	w	mu	sigma	u	est. error	sigma error	K2	K3	n2	n3
65% Mad2 P50	0.44	6.82	0.41	0.56	4.51	0.34	0.65	-0.01	0.02	0.65		12.29	
65% Mad2 P188	0.77	6.82	0.41	0.23	4.90	0.32	0.65	0.06	0.02	0.65		12.29	
80% Mad2	0.87	6.82	0.41	0.13	4.74	0.35	0.80	-0.05	0.02	0.65		12.29	
200% Mad2 P(259bp)	1.00	6.82	0.41				2.00	0.00	0.02	0.65		12.29	
Delta Mad2	0.00	6.82	0.41	1.00	3.77	0.15	0.00	0.00	0.02				
10% Mad2	0.00	6.82	0.41	1.00	3.81	0.15	0.10	0.00	0.02	0.65		12.29	
20% Mad2	0.00	6.82	0.41	1.00	3.97	0.15	0.20	0.00	0.02	0.65		12.29	
40% Mad2	0.00	6.82	0.41	1.00	4.09	0.16	0.40	-0.00	0.02	0.65		12.29	
Delta Mad3	0.00	6.82	0.41	1.00	3.86	0.11	0.00	0.00	0.02				
30% Mad3 (2)	0.63	6.82	0.41	0.37	4.77	0.30	0.30	0.00	0.02		0.25		2.11
60% Mad3	0.91	6.82	0.41	0.09	4.83	0.22	0.60	-0.00	0.02		0.25		2.11
120% Mad3	1.00	6.82	0.41				1.20	0.00	0.02		0.25		2.11
WT fusion	1.00	6.82	0.41				1.00	0.00	0.02				

Number of parameters: 38
log-posterior: -6.5152e+03

Figure S17: Maximum likelihood estimate for the parametrization of the fraction w of the subpopulation with wild type like SAC.

As described in the manuscript we used MEMO to study whether the fraction of wild type-like cells is independently affected by Mad2 and Mad3 perturbations (Model 1) or whether these two proteins act synergistically on this fraction (Model 2). To assess these competing hypotheses, we modeled the WT fraction of cells with a functional (= wild type-like) SAC for different Mad2 and Mad3 abundances using a product of the two Hill-type functions (1) and (2).

Model 1:

$$WT_{fracM2\&M3} = WT_{fracM2}(n_2, K_2, u_{Mad2_{rel}}, \epsilon_{Mad2}) \cdot WT_{fracM3}(n_3, K_3, u_{Mad3_{rel}}, \epsilon_{Mad3})$$

To take synergistic effects into account, in a second model the threshold parameters K_{ij} of these functions were described to be inversely proportional to the amount of the other protein (Model 2). For inputs $u \geq 1$

The output of this model equals the output of model 1, while for lower inputs the parameters K_i are increased, which results in lower model outputs.

Model 2:

$$WT_{fracM2\&M3} = WT_{fracM2}(n_2, K_{23}, u_{Mad2_{rel}}, \epsilon_{Mad2}) \cdot WT_{fracM3}(n_3, K_{32}, u_{Mad3_{rel}}, \epsilon_{Mad3})$$

with

$$K_{23} = K_2 \frac{a}{(a-1) + u_{Mad3_{rel}} + \epsilon_{Mad3}}$$

and

$$K_{32} = K_3 \frac{a}{(a-1) + u_{Mad2_{rel}} + \epsilon_{Mad2}}.$$

These models were parametrized by using the MLE of the fit of (1) and (2). The additional parameter a in Model 2 was determined independently by maximizing the likelihood of the three double perturbation experiments (65% Mad2 & 120% Mad3, 65% Mad2 & 60% Mad3, and 65% Mad2 & 30% Mad3, data in Figure S18) given the MLE for all other parameters. It was found to be 7.55. The BICs shown in Figure 6B and 6C in the manuscript were calculated from the likelihood of the three double perturbation experiments by taking into account that Model 2 has one parameter more than Model 1.

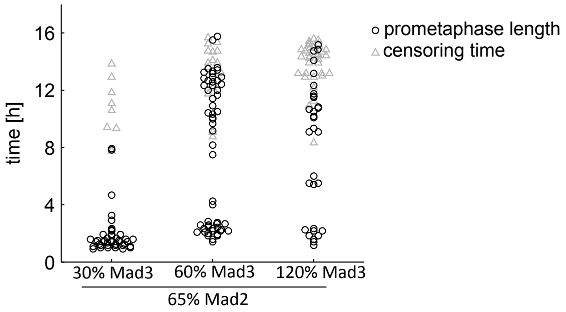

Figure S18: Datasets of double perturbation experiments. 65% Mad2 in combination with 30%, 60% and 120% Mad3.

S.4.4.2 Parametrization of $\mu(u)$ of a log-normally distributed subpopulation with dysfunctional SAC

Besides the weights of the mixture distributions also the parameters of the distributions can be parametrized. In the following this is shown exemplarily for the parameter μ of the log-normal distribution representing the subpopulation with dysfunctional SAC in datasets of Mad2. In case of Mad2 an increasing amount of protein positively correlates with SAC functionality and therefore with the length of the prometaphase time. Thus, we modeled the parameter μ to positively depend on the input u via a hill type function with an offset,

$$\mu_{\mathrm{Mad2}}(u) = \mu_{\mathrm{null}} + v_{\max} \frac{(u_{\mathrm{Mad2}_{\mathrm{rel}}} + \epsilon)^n}{K_{M2}^n + (u_{\mathrm{Mad2}_{\mathrm{rel}}} + \epsilon)^n}. \tag{3}$$

According to this function the time spend in prometaphase also increases with increasing protein amount. Figure S19 depicts the functional dependency of $\mu(u)$ resulting from the MLE (Figure S20). The parametrization of μ enables the prediction of μ for yet unobserved inputs u by inserting the relative amount of protein in the equation of the parametrization.

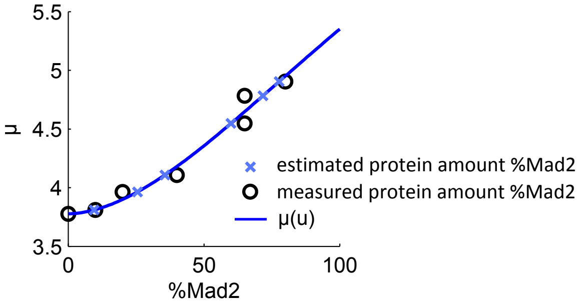

Figure S19: Functional dependency of the log-normal parameter μ on the input u for u = relative abundance of Mad2. Circles indicate the data for measured abundances, crosses for the protein abundances that result from estimation.

Dataset	component 1			component 2			input dependency						
	w	mu	sigma	w	mu	sigma	u	est. error	sigma error	KM2	mu_null	nh	vmax
Delta Mad2	0.00	6.83	0.42	1.00	3.78	0.15	0.00	0.00	0.02	1.53	3.78	1.81	4.99
10% Mad2	0.00	6.83	0.42	1.00	3.81	0.15	0.10	-0.00	0.02	1.53	3.78	1.81	4.99
20% Mad2	0.00	6.83	0.42	1.00	3.96	0.15	0.20	0.05	0.02	1.53	3.78	1.81	4.99
40% Mad2	0.00	6.83	0.42	1.00	4.11	0.16	0.40	-0.04	0.02	1.53	3.78	1.81	4.99
65% Mad2 P50	0.44	6.83	0.42	0.56	4.55	0.34	0.65	-0.05	0.02	1.53	3.78	1.81	4.99
65% Mad2 P188	0.80	6.83	0.42	0.20	4.78	0.34	0.65	0.07	0.02	1.53	3.78	1.81	4.99
80% Mad2	0.82	6.83	0.42	0.18	4.90	0.40	0.80	-0.02	0.02	1.53	3.78	1.81	4.99
WT Mad2	1.00	6.83	0.42				1.00	0.00	0.02				

Number of parameters: 30
log-posterior: -3.7918e+03
BIC: 7.7872e+03

Figure S20: Maximum likelihood estimate for the parametrization of the parameter μ of the log-normally distributed subpopulation with dysfunctional SAC.

S.5 Analysis of NGF-induced Erk1/2 phosphorylation data

The parametrization of subpopulation sizes and subpopulation mixture parameters can also be related to mechanistic models. To illustrate that, MEMO has been used to analyze data presented by Hasenauer et al. (2014). In this paper a mechanistic population model for NFG-induced Erk phosphorylation in primary sensory neurons from rat has been derived. Hasenauer et al. (2014) used this model to study single cell data collected using quantitative automated microscopy. In the following we reexamine their best model by providing MEMO with the dynamics of Erk phosphorylation and the single cell data on phosphorylated Erk from kinetic and dose response experiments. Furthermore, we study the effect of interval censoring by introducing censoring intervals to the originally uncensored data.

S.5.1 Mechanistic model of cell population

We consider model $\mathcal{M}_{\mathrm{H}3,3}$ developed in (Hasenauer et al., 2014). This model accounts for two subpopulations differing in the total concentration of the receptor $[\mathrm{TrkA}]_0$. The signaling in each subpopulation upon NFG stimulation is governed by

$$\begin{aligned}\frac{dx_1}{dt} &= k_1[\mathrm{NGF}]_0(k_3[\mathrm{TrkA}]_0 - x_1) - k_2 x_1\\ \frac{dx_2}{dt} &= (x_1 + k_4)(\mathrm{s}[\mathrm{Erk}]_0 - x_2) - k_5 x_2\\ y &= x_2,\end{aligned}$$

in which $x_1 = k_3[\mathrm{TrkA}:\mathrm{NGF}]$ is the effective activity of the receptor and $x_2 = s[\mathrm{pErk}]$ is the scaled abundance of phosphorylated Erk. We derived the analytical solution of the model output $y = x_2(t)$ and used it as parametric description of the medians of two log-normally distributed subpopulations differing in the amounts of the receptor $[\mathrm{TrkA}]_0$, as proposed by Hasenauer et al. (2014). The input u is given by $[\mathrm{NGF}]_0$, the concentration of NGF used for stimulation. As the receptor dynamics is fast, we assumed x_1 to be in quasi steady state, $\dot{x}_1 = 0$. Under this assumption it follows that

$$\begin{aligned}x_1(t) &= \frac{k_1[\mathrm{NGF}]_0 k_3[\mathrm{TrkA}]_0}{k_1[\mathrm{NGF}]_0 + k_2} = \mathrm{const.},\\ x_2(t) &= \frac{\mathrm{s}[\mathrm{Erk}]_0\left(x_1\left(1 - k_5\frac{\exp\left(-t\left(x_1+k_4+k_5\right)\right)}{k_4+k_5}\right) + k_4\right)}{(k_4+k_5+x_1)}.\end{aligned}$$

```
Type of mixture: log-normal
 -------------------------------------------------------
| Dataset   |      component 1      |      component 2      |
|----------|----------------------|----------------------|
|          |  w   |   mu   | sigma |  w   |   mu   | sigma |
|----------|----------------------|----------------------|
| 0 min    | 0.31 | -0.04 |  0.45 | 0.69 | -0.04 |  0.27 |
| 5 min    | 0.31 |  1.11 |  0.45 | 0.69 |  0.06 |  0.37 |
| 15 min   | 0.31 |  1.34 |  0.57 | 0.69 |  0.10 |  0.38 |
| 30 min   | 0.31 |  1.36 |  0.46 | 0.69 |  0.11 |  0.41 |
| 60 min   | 0.31 |  1.36 |  0.52 | 0.69 |  0.11 |  0.43 |
| 0 nM     | 0.31 | -0.04 |  0.54 | 0.69 | -0.04 |  0.41 |
| 0.001 nM | 0.31 | -0.01 |  0.71 | 0.69 | -0.04 |  0.45 |
| 0.01 nM  | 0.31 |  0.21 |  0.67 | 0.69 | -0.03 |  0.40 |
| 0.1 nM   | 0.31 |  0.93 |  0.59 | 0.69 |  0.03 |  0.43 |
| 1 nM     | 0.31 |  1.36 |  0.51 | 0.69 |  0.11 |  0.40 |
| 10 nM    | 0.31 |  1.43 |  0.48 | 0.69 |  0.12 |  0.36 |
 -------------------------------------------------------

Number of parameters: 30
log-posterior: -3.5852e+04
```

Figure S21: Maximum likelihood estimate for the parametrization of the parameter μ via the analytic solution of the population median over time.

S.5.2 Parameter estimation and uncertainty analysis

The unknown parameters of the population model are k_1, k_2, k_4, k_5 and $s[\mathrm{Erk}]_0$, as well as the effective subpopulation specific receptor concentrations, $k_3[\mathrm{TrkA}]_{0,1}$and $k_3[\mathrm{TrkA}]_{0,2}$, together with the parameters $\sigma_1(t,u)$ and $\sigma_2(t,u)$ of the log-normal distributions describing the two subpopulations in every experimental condition and the weighting factor w. To improve optimization performance, k_1 was rewritten as $k_2 \cdot K_D$ and $k_3[\mathrm{TrkA}]_{0,2}$ was replaced by $k_3[\mathrm{TrkA}]_{0,1} \cdot B$. K_D denotes the dissociation constant of the NGF-TrkA interaction while B denotes the relative difference of TrkA expression between the subpopulations. Furthermore, we used the base 10 logarithm of the data and therefore fitted normal distributions instead of log-normal distributions. We estimated the logarithm of the unknown parameters using multi-start local optimization using lower and upper bounds provided in (Hasenauer et al., 2014).

The MLE of the distribution parameters is shown in Figure S21. Model and data are in good agreement (manuscript, Figures 7B and 7C, and Figure S22), illustrating the applicability of MEMO in combination with mechanistic models for which an analytical solution of the model output can be calculated. The profile likelihoods of the parameters indicate that most of the parameters are well determined (Figure S23).
To evaluate the effect of interval censoring, we repeated the parameter estimation after binning the data in different numbers of bins. For the accurate handling of interval censoring, we find that the parameter estimates are consistent over a wide range of censoring levels (manuscript, Figure 7D).

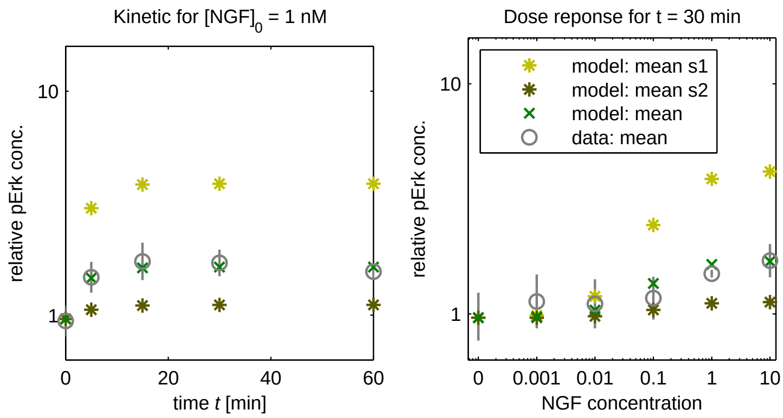

Figure S22: Mean and standard deviation of measured pErk levels (black) as well as the means calculated for the model (green: mean of subpopulation 1, blue: mean of subpopulation 2, red: mean of overall population).

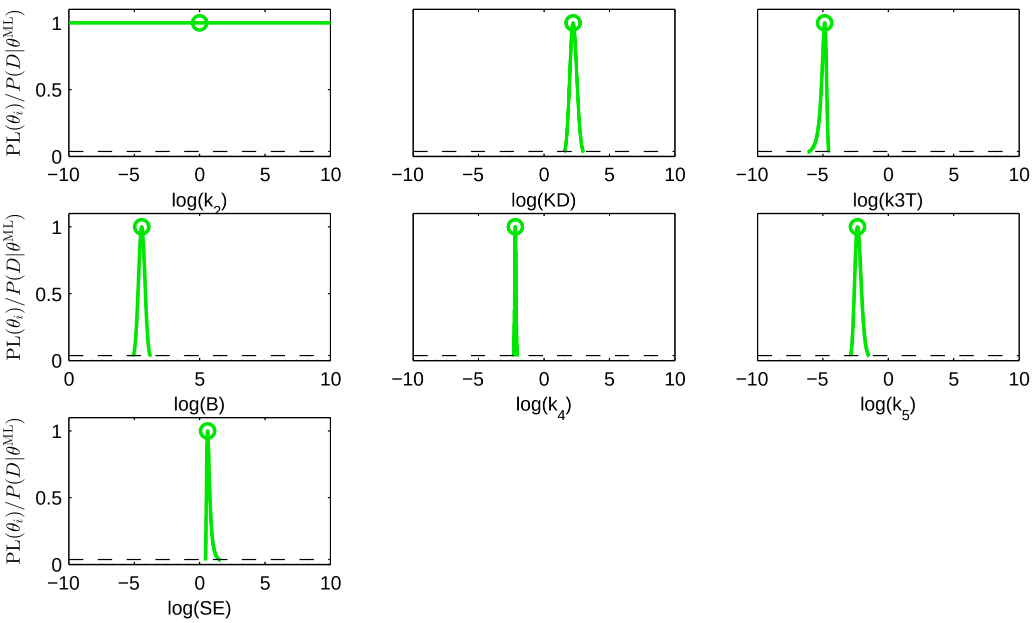

Figure S23: Profile likelihoods of the parameters parametrizing the medians of the two subpopulations.

S.6 Case study for right censored and right truncated data with algorithms published in Lee & Scott (2012)

For a comparison of performance on censored data we applied the algorithms provided in Lee & Scott (2012) to our setup of right censored artificial data. We did not make the comparison for interval censored data, since the algorithms of Lee & Scott (2012) do not consider interval censoring. Since the algorithms of Lee & Scott (2012) can only deal with mixtures of Gaussians we log-transformed the censored and uncensored datapoints, yielding exactly the same setup as we used to evaluate MEMO. The setup in Figure S24A is the setup used for Figure 2B in the manuscript. Since data beyond the censoring value were omitted, this setup corresponds to right truncation. Figure S24B corresponds to Figure 2D in the manuscript. For the right truncated data we found that the number of subpopulations was correctly inferred only when the censoring value is much greater than the distribution mean (Figure S24A). This is very similar to the results obtained with MEMO (manuscript, Figure 2B). In contrast, for right censored data, the EM algorithms developed by Lee & Scott (2012) robustly detects that the population consists merely of the log-normal distribution (Figure S24B). This is also very similar to the results obtained with MEMO (manuscript, Figure 2D).

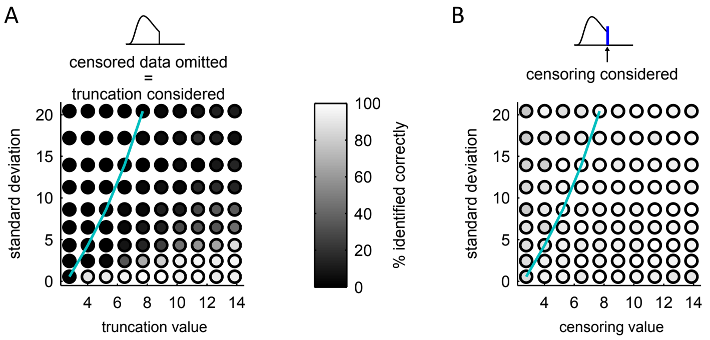

Figure S24: Inference of a log-normal distribution using (A) right truncated or (B) right censored data. The color of the circles encodes the frequency with which the correct number of subpopulations, here one, is selected. As a reference, the light blue curve indicates points for which the censoring value equals the mean of the log-normal distribution with $\mu = 1$ and the standard deviation indicated on the y-axis.

References

Buettner, F., Moignard, V., Göttgens, B., & Theis, F. J. (2014). Probabilistic pca of censored data: accounting for uncertainties in the visualization of high-throughput single-cell qpcr data. Bioinformatics, 30(13), 1867–1875.

Hasenauer, J., Hasenauer, C., Hucho, T., & Theis, F. J. (2014). ODE constrained mixture modelling: A method for unraveling subpopulation structures and dynamics. PLoS Comput. Biol., 10(7), e1003686.

Heinrich, S., Geissen, E., Kamenz, J., Trautmann, S., Widmer, C., Drewe, P., Knop, M., Radde, N., Hasenauer, J., & Hauf, S. (2013). Determinants for robustness in spindle assembly checkpoint signalling. Nature Cell Biology, 15(11), 1328–1339.

Johnsson, K., Wallin, J., & Fontes, M. (2016). Bayesflow: latent modeling of flow cytometry cell populations. BMC Bioinformatics, 17(1), 25.

Lee, G. & Scott, C. (2012). Em algorithms for multivariate gaussian mixture models with truncated and censored data. Computational Statistics & Data Analysis, 56(9), 2816–2829.

Li, R. & Murray, A. W. (1991). Feedback control of mitosis in budding yeast. Cell, 66(3), 519–531.

Nanita, S. C. (2013). Quantitative mass spectrometry independence from matrix effects and detector saturation achieved by flow injection analysis with real-time infinite dilution. Anal Chem, 85(24), 11866–11875.

Pyne, S., Hu, X., Wang, K., Rossin, E., Lin, T., Maier, L., Baecher-Allan, C., McLachlan, G., Tamayo, P., Hafler, D., De Jager, P., & Mesirov, J. (2009). Automated high-dimensional flow cytometric data analysis. Proc. Natl. Acad. Sci. U S A, 106(21), 8519–8124.

Pyne, S., Lee, S. X., Wang, K., Irish, J., Tamayo, P., Nazaire, M.-D., Duong, T., Ng, S.-K., Hafler, D., Levy, R., Nolan, G. P., Mesirov, J., & McLachlan, G. J. (2014). Joint modeling and registration of cell populations in cohorts of high-dimensional flow cytometric data. PLoS One, 9(7), e100334.

Shalek, A. K., Satija, R., Shuga, J., Trombetta, J. J., Gennert, D., Lu, D., Chen, P., Gertner, R. S., Gaublomme, J. T., Yosef, N., Schwartz, S., Fowler, B., Weaver, S., Wang, J., Wang, X., Ding, R., Raychowdhury, R., Friedman, N., Hacohen, N., Park, H., May, A. P., & Regev, A. (2014). Single-cell rna-seq reveals dynamic paracrine control of cellular variation. Nature, 510(7505), 363–369.

Visscher, K., Brakenhoff, G., & Visser, T. (1994). Fluorescence saturation in confocal microscopy. Journal of Microscopy, 175(2), 162–165.
